# The 2023 WSES guidelines on the management of trauma in elderly and frail patients

**DOI:** 10.1186/s13017-024-00537-8

**Published:** 2024-05-31

**Authors:** Belinda De Simone, Elie Chouillard, Mauro Podda, Nikolaos Pararas, Gustavo de Carvalho Duarte, Paola Fugazzola, Arianna Birindelli, Federico Coccolini, Andrea Polistena, Maria Grazia Sibilla, Vitor Kruger, Gustavo P. Fraga, Giulia Montori, Emanuele Russo, Tadeja Pintar, Luca Ansaloni, Nicola Avenia, Salomone Di Saverio, Ari Leppäniemi, Andrea Lauretta, Massimo Sartelli, Alessandro Puzziello, Paolo Carcoforo, Vanni Agnoletti, Luca Bissoni, Arda Isik, Yoram Kluger, Ernest E. Moore, Oreste Marco Romeo, Fikri M. Abu-Zidan, Solomon Gurmu Beka, Dieter G. Weber, Edward C. T. H. Tan, Ciro Paolillo, Yunfeng Cui, Fernando Kim, Edoardo Picetti, Isidoro Di Carlo, Adriana Toro, Gabriele Sganga, Federica Sganga, Mario Testini, Giovanna Di Meo, Andrew W. Kirkpatrick, Ingo Marzi, Nicola déAngelis, Michael Denis Kelly, Imtiaz Wani, Boris Sakakushev, Miklosh Bala, Luigi Bonavina, Joseph M. Galante, Vishal G. Shelat, Lorenzo Cobianchi, Francesca Dal Mas, Manos Pikoulis, Dimitrios Damaskos, Raul Coimbra, Jugdeep Dhesi, Melissa Red Hoffman, Philip F. Stahel, Ronald V. Maier, Andrey Litvin, Rifat Latifi, Walter L. Biffl, Fausto Catena

**Affiliations:** 1 Department of Emergency Minimally Invasive Surgery, Academic Hospital of Villeneuve St Georges, Villeneuve St Georges, France; 2 Department of General Minimally Invasive Surgery, Infermi Hospital, AUSL Romagna, Rimini, Italy; 3 General Surgery Department, American Hospital of Paris, Paris, France; 4 Department of Surgical Science, Unit of Emergency Surgery, University of Cagliari, Cagliari, Italy; 5 3rd Department of Surgery, Attikon General Hospital, National and Kapodistrian University of Athens (NKUA), Athens, Greece; 6 New Zealand Blood Service, Christchurch, New Zealand; 7 Unit of General Surgery I, IRCCS San Matteo Hospital of Pavia, University of Pavia, Pavia, Italy; 8 Unit of General Surgery, Esine Hospital, ASST Valcamonica, Esine, Italy; 9 Department of General Surgery, University Hospital of Pisa, Pisa, Italy; 10 Department of Surgery, Policlinico Umberto I Roma, Sapienza University, Rome, Italy; 11 Department of Surgery, Unit of General Surgery, University Hospital of Ferrara and University of Ferrara, Ferrara, Italy; 12 Division of Trauma Surgery, School of Medical Sciences, University of Campinas, Campinas, Brazil; 13 Unit of General and Emergency Surgery, Vittorio Veneto Hospital, Via C. Forlanini 71, 31029 Vittorio Veneto, TV Italy; 14 Department of Anesthesia, Level I, Trauma Center, Bufalini Hospital, Cesena, Italy; 15 UMC Ljubljana and Medical Faculty Ljubljana, Ljubljana, Slovenia; 16 Endocrine Surgical Unit - University of Perugia, Terni, Italy; 17 General Surgery Unit, Madonna del Soccorso Hospital, AST Ascoli Piceno, San Benedetto del Tronto, Italy; 18 Division of Emergency Surgery, Helsinki University Hospital and University of Helsinki, Helsinki, Finland; 19 Department of Surgical Oncology, Centro Di Riferimento Oncologico Di Aviano IRCCS, Aviano, Italy; 20 Department of General Surgery, Macerata Hospital, Macerata, Italy; 21 Dipartimento di Medicina, Chirurgia e Odontoiatria, Campus Universitario di Baronissi (SA) - Università di Salerno, AOU San Giovanni di Dio e Ruggi di Aragona, Salerno, Italy; 22 Istanbul Medeniyet University, Istanbul, Turkey; 23 Department of General Surgery, Rambam Health Care Campus, Haifa, Israel; 24 Ernest E Moore Shock Trauma Center at Denver Health, University of Colorado, Denver, CO USA; 25 Bronson Methodist Hospital/Western Michigan University, Kalamazoo, MI USA; 26 Department of Surgery, College of Medicine and Health Sciences, United Arab Emirates University, Al‑Ain, United Arab Emirates; 27 Ethiopian Air Force Hospital, Bishoftu, Oromia Ethiopia; 28 Department of General Surgery, Royal Perth Hospital and The University of Western Australia, Perth, Australia; 29 Department of Surgery, Radboud University Medical Center, Nijmegen, The Netherlands; 30 Emergency Department, Ospedale Civile Maggiore, Verona, Italy; 31 Department of Surgery, Tianjin Nankai Hospital, Nankai Clinical School of Medicine, Tianjin Medical University, Tianjin, China; 32 University of Colorado Anschutz Medical Campus, Denver, CO 80246 USA; 33 Department of Anesthesia and Intensive Care, Parma University Hospital, Parma, Italy; 34 Department of Surgical Sciences and Advanced Technologies, General Surgery Cannizzaro Hospital, University of Catania, Catania, Italy; 35 Fondazione Policlinico Universitario A. Gemelli IRCCS, Catholic University, Rome, Italy; 36 Department of Geriatrics, Ospedale Sant’Anna, Ferrara, Italy; 37 Department of Precision and Regenerative Medicine and Ionian Area, Unit of Academic General Surgery, University of Bari “A. Moro”, Bari, Italy; 38 Departments of Surgery and Critical Care Medicine, University of Calgary, Foothills Medical Centre, Calgary, AB Canada; 39 Department of Trauma, Hand and Reconstructive Surgery, University Hospital Frankfurt, Frankfurt, Germany; 40 Unit of Colorectal and Digestive Surgery, DIGEST Department, Beaujon University Hospital, AP-HP, University of Paris Cité, Clichy, France; 41 Department of General Surgery, MedAlliance, Albury, NSW Australia; 42 Department of Surgery, Government Gousia Hospital, DHS, Srinagar, India; 43 General Surgery Department, Medical University, University Hospital St George, Plovdiv, Bulgaria; 44 Hadassah Medical Center and Faculty of Medicine, Hebrew University of Jerusalem, Jerusalem, Israel; 45 Division of General Surgery, IRCCS Policlinico San Donato, University of Milan, Milan, Italy; 46 Division of Trauma and Acute Care Surgery, Department of Surgery, University of California Davis, Sacramento, CA USA; 47 Department of General Surgery, Tan Tock Seng Hospital, Novena, Singapore; 48 Department of Management, Ca’ Foscari University of Venice, Venice, Italy; 49 Collegium Medicum, University of Social Sciences, Łodz, Poland; 50 Department of Surgery, Royal Infirmary of Edinburgh, Edinburgh, UK; 51 Riverside University Health System Medical Center, Riverside, CA USA; 52 Guy’s and St Thomas’ NHS Foundation Trust, London, UK; 53 Department of Surgery, University of North Carolina, Surgical Palliative Care Society, Asheville, NC USA; 54 Department of Surgery, Brody School of Medicine, East Carolina University, Greenville, NC USA; 55 Harborview Medical Center, University of Washington, Seattle, WA USA; 56 Department of Surgical Diseases No. 3, Gomel State Medical University, University Clinic, Gomel, Belarus; 57 University of Arizona, Tucson, AZ USA; 58 Abrazo Health West Campus, Goodyear, Tucson, AZ USA; 59 Division of Trauma/Acute Care Surgery, Scripps Clinic Medical Group, La Jolla, CA USA; 60 Department of General and Emergency Surgery, Bufalini Hospital‐Level 1 Trauma Center, AUSL Romagna, Cesena, Italy

**Keywords:** Elderly, Geriatric patient, Trauma management, Imaging, Laboratory test, Trauma score, Resuscitation, Delirium, Pain control, Antibiotics, Thrombo-prophylaxis, Direct oral anticoagulants management, Vitamin K antagonists anticoagulants management, Palliative care, End of life, Frailty, Ageing

## Abstract

**Background:**

The trauma mortality rate is higher in the elderly compared with younger patients. Ageing is associated with physiological changes in multiple systems and correlated with frailty. Frailty is a risk factor for mortality in elderly trauma patients. We aim to provide evidence-based guidelines for the management of geriatric trauma patients to improve it and reduce futile procedures.

**Methods:**

Six working groups of expert acute care and trauma surgeons reviewed extensively the literature according to the topic and the PICO question assigned. Statements and recommendations were assessed according to the GRADE methodology and approved by a consensus of experts in the field at the 10th international congress of the WSES in 2023.

**Results:**

The management of elderly trauma patients requires knowledge of ageing physiology, a focused triage, including drug history, frailty assessment, nutritional status, and early activation of trauma protocol to improve outcomes. Acute trauma pain in the elderly has to be managed in a multimodal analgesic approach, to avoid side effects of opioid use. Antibiotic prophylaxis is recommended in penetrating (abdominal, thoracic) trauma, in severely burned and in open fractures elderly patients to decrease septic complications. Antibiotics are not recommended in blunt trauma in the absence of signs of sepsis and septic shock. Venous thromboembolism prophylaxis with LMWH or UFH should be administrated as soon as possible in high and moderate-risk elderly trauma patients according to the renal function, weight of the patient and bleeding risk. A palliative care team should be involved as soon as possible to discuss the end of life in a multidisciplinary approach considering the patient’s directives, family feelings and representatives' desires, and all decisions should be shared.

**Conclusions:**

The management of elderly trauma patients requires knowledge of ageing physiology, a focused triage based on assessing frailty and early activation of trauma protocol to improve outcomes. Geriatric Intensive Care Units are needed to care for elderly and frail trauma patients in a multidisciplinary approach to decrease mortality and improve outcomes.

**Graphical abstract:**

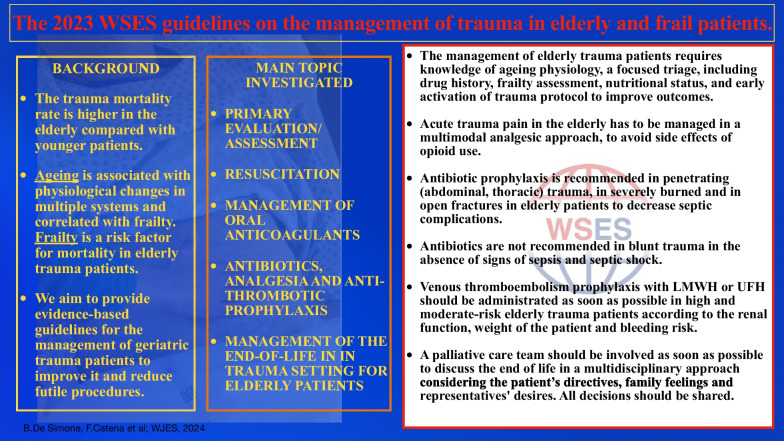

**Supplementary Information:**

The online version contains supplementary material available at 10.1186/s13017-024-00537-8.

## Background

With improvements in health and social care in the last century, the over-65-year-old patient cohort makes up a quarter of the population in the developed world. In the year 2000, the number of persons aged 65 years and older represented just more than 12% of the US population; by 2050, they are expected to make up more than 21% of the total population and almost 39% of trauma admissions, with an increasing of more than 20%. Patients aged 80 years and older that represent the group of “*oldest old”* patients, will increase to nearly 20 million persons by the year 2030 [1, 2].

In 2020, more than one-fifth (20.6%) of the EU population was aged 65 and over [2].

The longer life expectancy of the world population, who adopt an active lifestyle, and the effect of aging on patients’ physiology sustain trauma and mortality. Age-related anatomical modifications such as decreased muscle mass and strength, bone density, and joint flexibility and physiological changes, including decreased vision and hearing, slower reflexes, poorer balance, impaired motor and cognitive function associated with unrecognised frailty, make caring for geriatric patients challenging [3, 4].

Trauma is the fifth leading cause of death when all age groups are considered, the fourth leading cause of death in those aged 55–64 years and the ninth leading cause of mortality in patients aged 65 years and older [4–6].

The most common mechanism of injury in patients aged ≥ 65 is the ground-level fall. Six percent of ground-level falls patients will sustain a fracture, and 10–30% of these patients will have polytrauma, being elderly people more likely to sustain fractures of the cervical spine, ribs, hip, and extremities. Mortality rate in this age-group is reported to be as high as 7% [7–12]. Prevention strategies, endorsed recently by several western countries, to reduce falls in elderly such as home-based exercise programs and home safety interventions are effective to reduce the risk of falling but they have limited applications in active and independent people in the immediate future because of their high costs for healthcare systems [10].

Motor vehicle crashes are the second most common mechanism of injury among older patients, and the most common cause of traumatic mortality [4–7]. About one-quarter of all older adult victims of motor vehicle crashes sustain a chest injury which can exacerbate preexisting cardiopulmonary disease and increases the risk of significant complications, including pneumonia and respiratory failure [4–6].

Older adults are second only to children as victims of pedestrian injuries, but account for the largest percentage of the auto-pedestrian fatalities. The highest mortality rate in geriatric trauma is among pedestrians struck by a vehicle [7, 11–13].

Elderly women are also at high risk of burn injury, mainly due to home accidents, caused mostly by fire and scalding [3, 14]. Burns can have a devastating effect on geriatric patients, in whom mortality is significantly higher than in younger adults for any size and localization burn [15].

Geriatric patients are especially vulnerable to assault (the fourth most common mechanism of injury), resulting in 10% of geriatric trauma admissions. Geriatric victims of violence are 5 times more likely to die compared with younger victims [7, 11, 12].

Geriatric trauma mortality is high because of preexisting medical conditions, frailty and poor physiological reserve in elderly victims [16–19]. Eighty percent of geriatric trauma patients have at least one chronic disease, such as hypertension, arthritis, heart disease, pulmonary disease, cancer, diabetes, or history of stroke [4 Grabo]. These comorbidities, when combined with frailty result in more vulnerability to stress.

This is the raison why elderly trauma patients cannot be managed like adult younger trauma victims [18]. Deep understanding of their physiology is essential to provide them with proper treatment [20]. Important issues in improving the management and clinical outcomes of geriatric trauma include: (1) avoiding under-triage; (2) early, targeted, and aggressive care; and (3) early admission to an Intensive Care Unit (ICU). To accomplish these points, we need to early assess and manage “frail” patients. We aim to provide evidence-based guidelines for the management of geriatric trauma patients so as to improve it and reduce futile procedures.

## Methodology

According to PICO [21] criteria, the coordinator of the project identified research areas, main topics and questions correlated to geriatric trauma management to investigate. The main topics and PICO questions are summarized in the Table 1.

**Table 1:** Topics and PICO questions

MAIN TOPIC	PICO QUESTIONS
DEFINITIONS	Q.1.1: Which trauma patient is defined as “old” at initial evaluation?
	Q 1.2: When a patient is considered “physiologically old” and does he/she deserve different management after (blunt and penetrating) trauma?
PRIMARY EVALUATION/ASSESSMENT	Q 2.1: Which injury (physiological and anatomical) scores are higher predictive of outcome in evaluating elderly patients for trauma?
	Q 2.2: Which clinical features do better define the hemodynamic instability in geriatric trauma patients?
	Q 2.3: Which laboratory tests and biological markers are useful to evaluate the elderly trauma patient before resuscitation?
	Q 2.4: Which imaging studies are useful to better evaluate trauma elderly patients?
RESUSCITATION	Q 3.1: What early resuscitative protocol including intravenous fluids, blood transfusions or vasopressors should be used to manage geriatric trauma patients at primary evaluation?
	Q 3.2: Which are the resuscitation endpoints in elderly trauma patients?
	Q 3.3: Which vasopressors are indicated in comorbid elderly injured patients?
	Q 3.4: Vasopressors treatments versus permissive hypotension in geriatric trauma patients: which are the clinical parameters and laboratory tests to consider in the choice?
	Q 3.5: How intraoperative hypotension status is correlated with delirium in geriatric patients?
MANAGEMENT OF ORAL ANTICOAGULANTS	Q 4.1: Which blood tests are useful to evaluate geriatric patients with anticoagulant drugs in trauma setting?
	Q 4.2: Which reversal protocol is indicated in patients in treatment with vitamin K antagonists?
	Q 4.3: Which reversal protocol is indicated in patients in treatment with direct oral anticoagulants (DOACs) ?
ANTIBIOTICS, ANALGESIA AND ANTI-THROMBOTIC PROPHYLAXIS	Q 5.1: When is it indicated to administer antibiotics in elderly trauma patients?
	Q 5.2: How to control pain in elderly patients admitted for trauma?
	Q 5.3: When and how is indicated to perform thrombo-prophylaxis in elderly trauma patients?
MANAGEMENT OF THE END-OF-LIFE IN IN TRAUMA SETTING FOR ELDERLY PATIENTS	Q 6.1: Which are the clinical features and vital parameters to define the elderly patient at end of life after trauma?
	Q 6.2: Could palliative management be useful in the management of an elderly patient at the end of life?

Six working groups of experienced acute care and emergency surgeons were constituted to carry out a focused systematic review about the topic assigned, using PubMed, EMBASE, Google Scholar, and the Cochrane Central Register of Controlled Trials databases. according to PRISMA methodology [22]. Literature search was concluded in May 2023, limited to articles in English language and focused on the analysis of previously published systematic reviews with/without meta-analysis, randomized controlled trials, and observational studies (retrospective, prospective, and registry studies). The coordinator supervised each step of literature searching, study selection, the final presentation of evidence and wrote the manuscript.

Each working group provided a focused draft and a variable number of statements and recommendations according to the Grading of Recommendations, Assessment, Development and Evaluation (GRADE) [23]. The provisional statements and the supporting literature were reviewed and discussed by email/call conferences and modified if necessary. Controversies statements and recommendations were validated with a Delphi consensus of WSES experts [24].

The final manuscript was discussed during the WSES Congress held in Pisa in June, 2023. Comments and suggestions were implemented to improve the recommendations in the geriatric trauma management.

The recommendations are summarised in Table 2.

**Table 2:** List of recommendations

PICO QUESTIONS	RECOMMENDATIONS
Q.1.1: Which trauma patient is defined as “old” at initial evaluation?	We suggest early trauma protocol activation in patients aged ≥ 55 years old [Weak recommendation based on a low level of evidence 2C] We recommend to carefully evaluate injured patients aged ≥ 55-year-old for potential high risk of mortality and to avoid under-triage [Strong recommendation based on a low level of evidence 1C]
Q 1.2: When a patient is considered “physiologically old” and does he/she deserve different management after (blunt and penetrating) trauma?	We suggest an early and rapid assessment of the patient including vital signs on presentation, mechanism of injury, injury severity and frailty including comorbidities and medication history to identify vulnerable trauma patients [Weak recommendation based on low level of evidence 2C] We recommend assessing frailty in all elderly trauma patients [Strong recommendation based on a moderate level of evidence 1B]
Q 2.1: Which injury (physiological and anatomical) scores are higher predictive of outcome in evaluating elderly patients for trauma?	We suggest evaluating elderly patients for trauma through the Geriatric Trauma Outcome Score (GTOS) to predict in-hospital mortality and the Trauma-Specific Frailty Index in order to identify patients at highest risk of poor outcome [Weak Recommendation, based on Moderate Quality of Evidence, 2B]
Q 2.2: Which clinical features do better define the hemodynamic instability in geriatric trauma patients?	We recommend keeping a lower threshold for trauma protocol activation in geriatric patients, with triage set points of heart ratio 90 bpm and systolic blood pressure less than 110 mmHg [Strong Recommendation, based on Moderate Quality of Evidence, 1B]
Q 2.3: Which laboratory tests and biological markers are useful to evaluate the elderly trauma patient before resuscitation?	We recommend performing an early blood gas (arterial or venous) for baseline base-deficit or a lactic acid assessment in geriatric trauma patients [Strong Recommendation, based on Moderate Quality of Evidence, 1B]
Q 2.4: Which imaging studies are useful to better evaluate trauma elderly patients?	We recommend a low threshold for initial imaging with CT scan in geriatric trauma patients. The diagnostic yield of a contrast-enhanced CT outweighs the risk of contrast-induced nephropathy, especially in view of the potential, dramatic effects of under-triage [Strong Recommendation, based on Moderate Quality of Evidence, 1B]
Q 3.1: What early resuscitative protocol including intravenous fluids, blood transfusions or vasopressors should be used to manage geriatric trauma patients at primary evaluation?	We recommend that every trauma center provides meticulous triage criteria to recognize the need to early activate resuscitative protocols for elderly patients. These triaging criteria should include physical examination, vital signs, blood gas analysis, and medical history, emphasizing clinical conditions and drug history that may guide resuscitative therapies, early coagulative support, and the need to correct coagulopathies, and minimise fluids [Strong recommendation based on moderate quality of evidence 1B] We recommend rapid recognition and correction of coagulation disorders related to trauma or chronic medication intake in elderly patients. [Strong recommendation based on moderate quality of evidence 1B] We recommend performing serial base deficit assessment and lactate levels as markers of occult hypoperfusion in addition to close monitoring of vital parameters trend (heart rate, blood pressure, respiratory rate, urinary output), and mental status in elderly patients in a dedicated intensive geriatric care unit [Strong recommendation based on moderate-low level quality of evidence 1B] We suggest considering carefully to administer inotropic agents in selected non-responding elderly patients to target resuscitation [Weak recommendation based on low level of evidence 2C]
Q 3.2: Which are the resuscitation endpoints in elderly trauma patients?	We recommend evaluating the indication for invasive versus non-invasive hemodynamic monitoring on a case-by-case basis in injured elderly patients. Hypoperfusion should be ruled out by serial base deficit assessments and lactate concentration [Strong recommendation based on moderate-low level of evidence 1B] We suggest the implementation of POCUS in monitoring the cardiac function and blood volume in elderly injured patient, if skills are present. Invasive hemodynamic monitoring should be reserved in selected cases, to critically ill elderly trauma patients who have hypotension, significant injuries (as defined by an Abbreviated Injury Score > 3 or a Trauma Score < 15), or uncertain cardiovascular and/or fluid status [Weak recommendation based on moderate and low level of evidence 2B]
Q 3.3: Which vasopressors are indicated in comorbid elderly injured patients?	We recommend against the routine use of vasopressors in elderly injured patients presenting with hypotension caused by hemorrhage [Strong recommendation based on high-moderate level of evidence 1A] We recommend identifying the cause of hypoperfusion and assessing preexisting conditions and pharmacologic history before choosing a vasopressor in managing trauma in an elderly patient [Strong recommendation based on a high-low quality level of evidence 1A] We suggest using norepinephrine in elderly patients suffering from neurogenic shock. The dose to be used must be the lowest to guarantee tissue perfusion. The possible onset of cardiac arrhythmia and possible hypotensive effects should be monitored [Weak recommendation based on a moderate-low quality level of evidence 2B]
Q 3.4: Vasopressors treatments versus permissive hypotension in geriatric trauma patients: which are the clinical parameters and laboratory tests to consider in the choice?	We recommend to carefully evaluate to implement permissive hypotension in managing selected elderly trauma patients. Tissue perfusion has to be constantly monitored by base excess level, arterial lactates dosage, urine output, and when possible, neurologic assessment. [Strong recommendation based on a high-low quality level of evidence 1A]
Q 3.5: How intraoperative hypotension status is correlated with delirium in geriatric patients?	We suggest assessing, as early as possible, the risk factors for the onset of delirium because it is related to unfavourable outcomes in trauma geriatric patients. [Weak recommendation based on a moderate-low quality level of evidence 2B]
Q 4.1: Which blood tests are useful to evaluate geriatric patients with anticoagulant drugs in trauma setting?	We recommend performing routinely the common coagulation assays in elderly patients including the Activated Partial Thromboplastin Time (aPTT), Thromboplastin Time (TT), Prothrombin Time (PT), INR, and anti-Xa levels to assess early anticoagulants exposure in the trauma setting. There is not enough evidence to support the routinely use of TEG or ROTEM in elderly trauma patients. Further studies are necessary to determine their role. [Strong recommendation based on a moderate level quality of evidence 1B]
Q 4.2: Which reversal protocol is indicated in patients in treatment with vitamin K antagonists?	We recommend administrating a reversal agent in elderly trauma patients anticoagulated with oral vitamin K antagonists who present with bleeding, not responding to supportive measures, major life-threatening bleeding, bleeding located in critical organs (central nervous system, abdominal, thoracic), or needing urgent surgical or invasive procedures [Strong recommendation based on a moderate level quality of evidence 1B] We recommend using the reversal protocol including intravenous four factor prothrombin complex concentrates (4F-PCCs) and 5 mg intravenous vitamin K in case of life-threatening bleeding and/or urgent surgical procedures. Further doses should be administered if needed to achieve INR < 1.5 [Strong recommendation based on a high level quality of evidence 1A] We recommend giving Fresh frozen plasma (FFP) as oral vitamin K antagonists (VKAs) agent reversal only if no other treatment is available [Strong recommendation based on a moderate quality level of evidence 1B] We do not recommend the use of recombinant activated coagulation factor VII (rFVIIa) as first-line VKA reversal agent [Strong recommendation based on a low level of quality evidence 1C]
Q 4.3: Which reversal protocol is indicated in patients in treatment with direct oral anticoagulants (DOACs) ?	We recommend an early assessment of laboratory coagulation tests and direct measurements of DOAC levels, if quantitative tests are available, in elderly trauma patients receiving or suspected of having received a DOAC before deciding for reversal due to the thromboembolic risk [Strong recommendation based on a moderate level quality of evidence 1B] We suggest the administration of DOACS reversal agents only in critically ill patients with dosable plasma DOAC levels and presenting with hemorrhagic shock not responding to resuscitation, when level of DOACS can be assessed [Weak recommendation based on a moderate-low quality of evidence 2B] If the trauma patient with uncontrolled life-threatening bleeding, was treated with dabigatran (anti-FIIa activity), the suggested reversal protocol is to administer idarucizumab 5 g IV. If idarucizumab is not available, 50 units/kg IV of activated prothrombin complex concentrates (APCC) may be administrated [Weak recommendation based on a moderate-low quality level of evidence 2B] In patients with rivaroxaban-associated or apixaban-associated (FX inhibitors) life-threatening and uncontrolled bleeding, the suggested reversal protocol is the administration of andexanet alfa as an intravenous bolus of 400 mg over 15 min followed by a continuous infusion of 480 mg over 2 h (low dose) or 800 mg over 30 min followed by a continuous infusion of 960 mg over 2 h (high dose), according to the last dose of DOAC and the size of the dose. If andexanet alfa is not available, 2000 units of four-factor prothrombin complex concentrates (PCC) may be administrated [Weak recommendation based on a moderate-low quality level of evidence 2B]
Q 5.1: When is it indicated to administer antibiotics in elderly trauma patients?	We recommend antibiotic prophylaxis in penetrating (abdominal, thoracic) trauma, in severely burned and in open fractures in elderly patients to decrease septic complications [Strong recommendation based on a high-moderate quality level of evidence 1A] We recommend early empiric antibiotic therapy in patients presenting with signs of sepsis and septic shock and high risk patients (obesity, immunocompromised, high ASA score) in penetrating abdominal trauma, which should be active against common bacteria causing surgical site infections in peritonitis, such as *Escherichia coli* or other *Enterobacteriales* or *Clostridiales* [Strong recommendation based on a moderate quality level of evidence 1B] We recommend against the administration of antibiotics in blunt trauma in absence of signs of sepsis and septic shock [Strong recommendation based on a moderate-low quality level of evidence 1B]
Q 5.2: How to control pain in elderly patients admitted for trauma?	We recommend a regular administration of intravenous acetaminophen every 6 h as first line treatment in managing acute trauma pain in the elderly in a multimodal analgesic approach [Strong recommendation based on high quality level of evidence 1A] We suggest considering to add NSAIDs in elderly patients presenting with severe pain, taking into account potential adverse events and pharmacological interactions [Weak recommendation based on a moderate quality level of evidence 2B] We recommend the implementation of Multi-Modal-Analgesia approach (MMA) in trauma setting for elderly injured patients including acetaminophen, gabapentinoids, NSAIDs, lidocaine patches, and tramadol and opioids only for breakthrough pain for the shortest period of administration at the lowest effective dose [Strong recommendation based on a moderate quality level of evidence 1B] We recommend peripheral nerve blocks placement in elderly patients with acute hip fractures at the time of presentation to reduce preoperative and postoperative opioid use for analgesia [Strong recommendation based on a high quality level of evidence 1A] We suggest the adoption of epidural analgesia and regional anaesthesia to control severe pain in acute hip fractures in selected elderly patients [Weak recommendation based on a moderate quality level of evidence 2B] In elderly patients with ribs fractures, we recommend the association of systemic analgesic treatment with thoracic epidural and paravertebral blocks to offer an adequate pain control with limited contraindications and improvement in respiratory function, reducing opioid consumption, infections and delirium, if skills are available [Strong recommendation based on a high quality level of evidence 1A] We recommend to routinely consider the use of epidural or spinal analgesia for management of postoperative pain in elderly patients who undergo major thoracic and abdominal procedures for trauma, if skills are available [Strong recommendation based on a high-quality level of evidence 1A] We recommend carefully evaluating the use of neuraxial and plexus blocks for patients receiving anticoagulants to avoid bleeding and complications [Strong recommendation based on a high-quality level of evidence 1A] We suggest the implementation of non-pharmacological measures such as immobilizing limbs and applying dressings or ice packs in conjunction with drug therapy*,* in control acute pain in elderly patients in the trauma setting [Weak recommendation based on a very low level of evidence 2D]
Q 5.3: When and how is indicated to perform thrombo-prophylaxis in elderly trauma patients?	We recommend administering venous thromboembolism prophylaxis with LMWH or UFH as soon as possible in high and moderate risk elderly patients in the trauma setting according to the renal function, weight of the patient and bleeding risk [Strong recommendation based on a low quality level of evidence 1C] If pharmacological prophylaxis of venous thromboembolism is contraindicated, we recommend mechanical prophylaxis [Strong recommendation based on a low quality level of evidence 1C]
Q 6.1: Which are the clinical features and vital parameters to define the elderly patient at end of life after trauma?	We recommend discussing in a multidisciplinary approach the end of life in an elderly patient in the trauma setting. The decision should be considering the patient’s directives, family feelings and representatives desires and should be shared [Strong recommendation based on a low-very low quality of evidence 1D]
Q 6.2: Could palliative management be useful in the management of an elderly patient at the end of life?	We recommend involving as soon as possible the palliative care team in managing an elderly severely injured patient at the end-of-life status [Strong recommendation based on a low-very low quality level of evidence 1C]

### Notes on the use of these guidelines

The 2023 WSES geriatric trauma guidelines are the result of an extensive review of the literature and a validation by a consensus of experts in the field. The statements and recommendations provided in this work do not represent a standard of practice but a suggested plan of care, based on the best available evidence and the consensus of experts, but they do not exclude other approaches as being within the standard of practice. These guidelines should be used and tailored by the treating surgeons and individualized for each patient depending on the setting and should not be followed blindly.

## Results

### Definitions

**Key Question 1.1**

**Which trauma patient is defined as “old” at initial evaluation?**

**Statement 1.1.1**

The chronological age does not correspond to the biological age. Aging is correlated with para-physiological changes in organ systems with altered response to trauma, compared with younger injured patients [QoE MODERATE B].

**Statement 1.1.2**

Patients aged ≥ 55 may require dedicated trauma care, because they may have high mortality rates after trauma [QoE LOW C].

**Statement 1.1.3**

The age of 65 is most often used referring to “old”, “elderly” or “geriatric” patients [QoE HIGH A].

**Recommendation 1.1**

We suggest early trauma protocol activation in patients aged ≥ 55 years old **[Weak recommendation based on a low level of evidence 2C].**

We recommend to carefully evaluate injured patients aged ≥ 55-year-old for potential high risk of mortality and to avoid under-triage **[Strong recommendation based on a low level of evidence 1C].**

### Summary of evidence and discussion

There are different ways of defining elderly people. Statistics on ageing generally categorize older people as being above a certain age threshold. Despite that, different cut-off levels of age have been suggested, generally a patient is defined as “geriatric” when aged 65 years old. The United Nations (UN) noted in World Population Ageing 2019 that older people are commonly defined as those aged from 60 or 65 years or more, while the World Health Organisation (WHO) states that older people in developed world economies are commonly defined as those aged 65 years or more. The WHO uses an alternative definition, whereby an older person is defined as someone who has already passed the median life expectancy at birth [25].

In trauma management, recent data suggest that mortality as adjusted for injury severity scale (ISS) increases at the age of 70 years, making the age of 70 the cutoff at which to consider a patient with trauma elderly or geriatric [26]. This notion is distinct from Advanced Trauma Life Support (ATLS) teaching, which recommends transportation to a trauma center for any patient older than 55 years. The Eastern Association for the Surgery of Trauma (EAST) guidelines which defines patients older than 65 years as elderly [27, 28].

Recently, a large multicenter analysis of 255,099 patients reported a significant increase in mortality at ages of 55, 77, and 82 years suggesting that trauma patients older than 55 years have to be considered for inclusion in geriatric trauma protocols. Furthermore, patients aged above 77 and at 82 years may need additional specialized care considerations. As age increased, patients were more female, have more dementia, sustain a ground level fall, and are more likely to be discharged to a skilled nursing facility after admission for trauma [29]. Although there is no consensus on an age cutoff for a patient with trauma to be considered elderly, the age of 65 is most often used in the trauma literature. Nevertheless, patients aged 55 and older are at high risk for mortality after trauma.

**Key Question 1.2**

**When is a patient considered** “**physiologically old” and does he/she deserve different management after (blunt or penetrating) trauma?**

**Statement 1.2.**

Frailty, hearth diseases, hepatic diseases, renal diseases, and cancer according to their stage and severity are risk factors for mortality in trauma patients [QoE low C].

**Recommendation 1.2**

We suggest an early and rapid assessment of the patient including vital signs on presentation, mechanism of injury, injury severity and frailty including comorbidities and medication history to identify vulnerable trauma patients **[Weak recommendation based on low level of evidence 2C].**

We recommend assessing frailty in all elderly trauma patients **[Strong recommendation based on a moderate level of evidence 1B].**

### Summary of evidence and discussion

Older adults are becoming increasingly involved in major trauma, which is often defined as an Injury Severity Score greater than 15 [30]. One-third of all injury-related deaths among males and two-thirds of such deaths among females occur in those aged 65 years or older. The care of major trauma in this growing age group remains challenging [27, 30–33]. Older patients with trauma are at risk for increased morbidity and mortality and prolonged hospital stay [26, 34–36]. Older patients experience major trauma from low-velocity mechanisms, such as falls from 1 m or less [37]. This may partially explain an under-triage of older patients, which delays activating the trauma team and transfer to a trauma center [38–45]. Chronological age is not a physiological age. Trauma outcomes in older patients are worse for those with comorbidity. A population-based study focused on assessing the impact of pre-existing conditions on mortality and morbidity in trauma patients older than 65 years. It enrolled 33,781 patients and showed an overall mortality of 7.6%. For each 1-year increase in age beyond age 65, odds of dying after geriatric trauma increased by 6.8%. When presenting vital signs, Glasgow Coma Scale (GCS) score, and ISS were adjusted for, hepatic disease, renal disease and cancer were risk factors for mortality. Furthermore, chronic steroid use increased the odds of death after geriatric trauma, whereas Coumadin therapy did not [16].

A prospective cohort study of 250 (median age of 80 years old) patients at a level I trauma center reported the frailty was present in 44% and was correlated with increased in-hospital complications such as cardiac, pulmonary, infectious, hematologic, renal, reoperation, and worse discharge disposition. Patients who died had more frailty [19]. Frailty is a syndrome of decreased physiological reserve and resistance to stressors, which results in worsening mobility and disability, hospitalizations, complications, and death [19].

Primary evaluation and triage of older people victims of a trauma, which includes clinical exam and objective assessment is challenging because of the physiologic differences between older and younger patients. Kehoe and colleagues [46] reported that older patients with a traumatic brain injury are often evaluated with a higher GCS score compared with younger patients. Heffernan and colleagues [47] reported also an increased mortality in patients aged 65 or older with trauma admitted with a systolic blood pressure less than 110 mm Hg (vs. > 95 mm Hg in younger patients) and heart rate greater than 90 beats/min (vs. > 130 beats/min in younger patients). In fact, older patients with trauma may have chronic occult hypoperfusion, which makes the presence of “normal” initial vital signs unreliable. Elderly patients frequently have higher blood pressure, therefore, a “normal” blood pressure may be hypotension in the elderly. Other examples include modification of conventional GCS cut-off values [48] and initial vital signs [47, 49] for older patients. Other authors have recommended using markers such as serum lactate level and base deficit [50–54] as alternative predictors of mortality. There is a need to modify trauma care of the elderly to improve the clinical outcome [55].

## Primary evaluation/assessment

**Key Question 2.1**

**Which injury (physiological and anatomical) scores are stronger predictors of outcome in evaluating elderly patients for trauma?**

**Statement 2.1.1**

Geriatric trauma patients are usually under-triaged to trauma centers due to low energy mechanisms of injury, unreliability of vital signs, and the use of medications that can obscure the physiologic response to trauma. Specific triaging scores can be used to predict outcomes in geriatric trauma patients and guide the triage decision-making process towards transfer to a Level I trauma centers and aggressive treatment (QoE moderate B).

**Recommendation 2.1**

We suggest evaluating elderly patients for trauma through the Geriatric Trauma Outcome Score (GTOS) to predict in-hospital mortality and the Trauma-Specific Frailty Index to identify patients at highest risk of poor outcome **[Weak Recommendation, based on Moderate Quality of Evidence, 2B]**.

### Summary of evidence and discussion

**Fig. 1:**
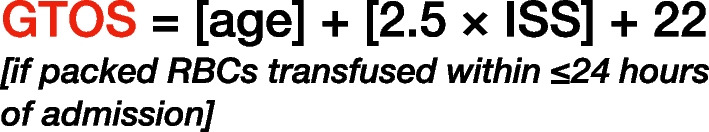
The Geriatric Trauma Outcome Score (GTOS)

Several scoring systems, with the purpose of supporting decision making, have been proposed to accurately predict outcomes for geriatric trauma patients. Age ≥ 65 years has shown to be an independent risk factor for increased mortality in trauma, controlled for the same Injury Severity Score (ISS), with a 2.4–5.6 greater risk of death [16, 26, 56, 57]. However, the risk of death from trauma seems to increase earlier, at the age of 56 [58]. With the purpose of predicting in-hospital mortality in patients over the age of 65 years, in 2015 Zhao et al. developed an objective tool based on the covariates of age, ISS and transfusion requirements during the first 24 h of care. The Geriatric Trauma Outcome Score (GTOS) **(**Fig. 1**)** uses a formula that is [age] + [2.5 × ISS] + 22 (if packed red blood cells transfused ≤ 24 h of admission). In practice it showed to accurately predict continuous odds of mortality across a spectrum of injury severity. In the original publication by Zhao et al*.*, the area under the receiver operating characteristic curve for the GTOS model was 0.82 [59]. Afterwards, the Prognostic Assessment of Life and Limitations After Trauma in the Elderly (PALLIATE) consortium [60] confirmed that the GTOS accurately predicts an elderly trauma patient's probability of dying during the index admission after injury, with an area under the curve applied to the validation sample of 0.86. Conversely, the GTOS does not seem to be a reliable prediction of 1-year mortality [61].

Recently, Ravindranath et al. evaluated retrospectively all elderly trauma patients admitted to the State Trauma Unit (Western Australia) between 2009 and 2019. Of the 57.473 trauma admissions during the study period, 15.034 (26.2%) were ≥ 65-year old. The ability of the GTOS to predict mortality was good (area under the curve 0.838, 95% CI 0.821–0.855), and better than either age (area under the curve 0.603, 95% CI 0.581–0.624) or ISS alone (area under the curve 0.799, 95% CI 0.779–0.819) alone. Noteworthy, the GTOS score (area under the curve 0.683, 95% CI 0.591–0.775) was inferior to the APACHE III (area under the curve 0.783, 95% CI 0.699–0.867) in predicting mortality for patients requiring intensive care. The calibration of the GTOS was reasonable when the predicted risk of death was < 50%, whereas when the predicted risk of death was > 50%, the model tended to be over pessimistic by overestimating the risks of death [62].

Both the ISS and GTOS trauma scoring systems were confirmed to be predictive of mortality in the study by Egglestone et al*.*, with an area under the curve of 0.66 (95% CI 0.59–0.74) for the ISS, and 0.68 (95% CI 0.61–0.76) for the GTOS. The optimal cut-off points were ≥ 28 and ≥ 142, for ISS and GTOS, respectively [63]. In the study by Jiang et al*.*, compared with APACHE II and SAPS II (Simplified acute physiology score II), the ISS, NISS (New Injury Severity Score), and TRISS (Trauma and Injury Severity Score) appeared to be better predictors of in-hospital mortality in elderly trauma patients. The area under the curve for the ISS was 0.807, 0.850 for the NISS, 0.828 for the TRISS, 0.715 for the APACHE II, and 0.725 for SAPS II (Simplified acute physiology score II) [64].

Although the GTOS seems to predict mortality in elderly trauma patients quite accurately, this score highly relies on ISS judgments, which are known for their subjectivity and suboptimal inter-observer reliability [65].

By conducting a receiver operating characteristic analysis, Scherer et al. performed a comparison with GTOS and the Revised Injury Severity Classification II (RISC-II) Score on a total of 58.055 geriatric trauma patients (mean age 77 years). Univariable models led to the following variables: age 80 years, need for packed red blood cells (PRBC) transfusion prior to intensive care unit (ICU), American Society of Anesthesiologists (ASA) score 3, Glasgow Coma Scale (GCS) 13, Abbreviated Injury Scale (AIS) in any body region 4. The maximum GERtality constructed on these five-variable score was 5 points. A mortality rate of 72.4% was calculated in patients with the maximum GERtality score. Mortality rates of 65.1 and 47.5% were encountered in patients with GERtality scores of 4 and 3 points, respectively. The area under the curve for the accuracy of mortality prediction was 0.784 and 0.879 for the GTOS and the RISC-II, respectively, whereas the novel GERtality score yielded an accuracy of 0.803. The new GERtality score seems to be an user-friendly and adequate in-hospital mortality prediction model for severely injured geriatric trauma patients, as it includes only five easily assessable patient variables, which makes it practical and simple to calculate. However, further studies should validate the novel GERtality score on different datasets [66].

**Fig. 2:**
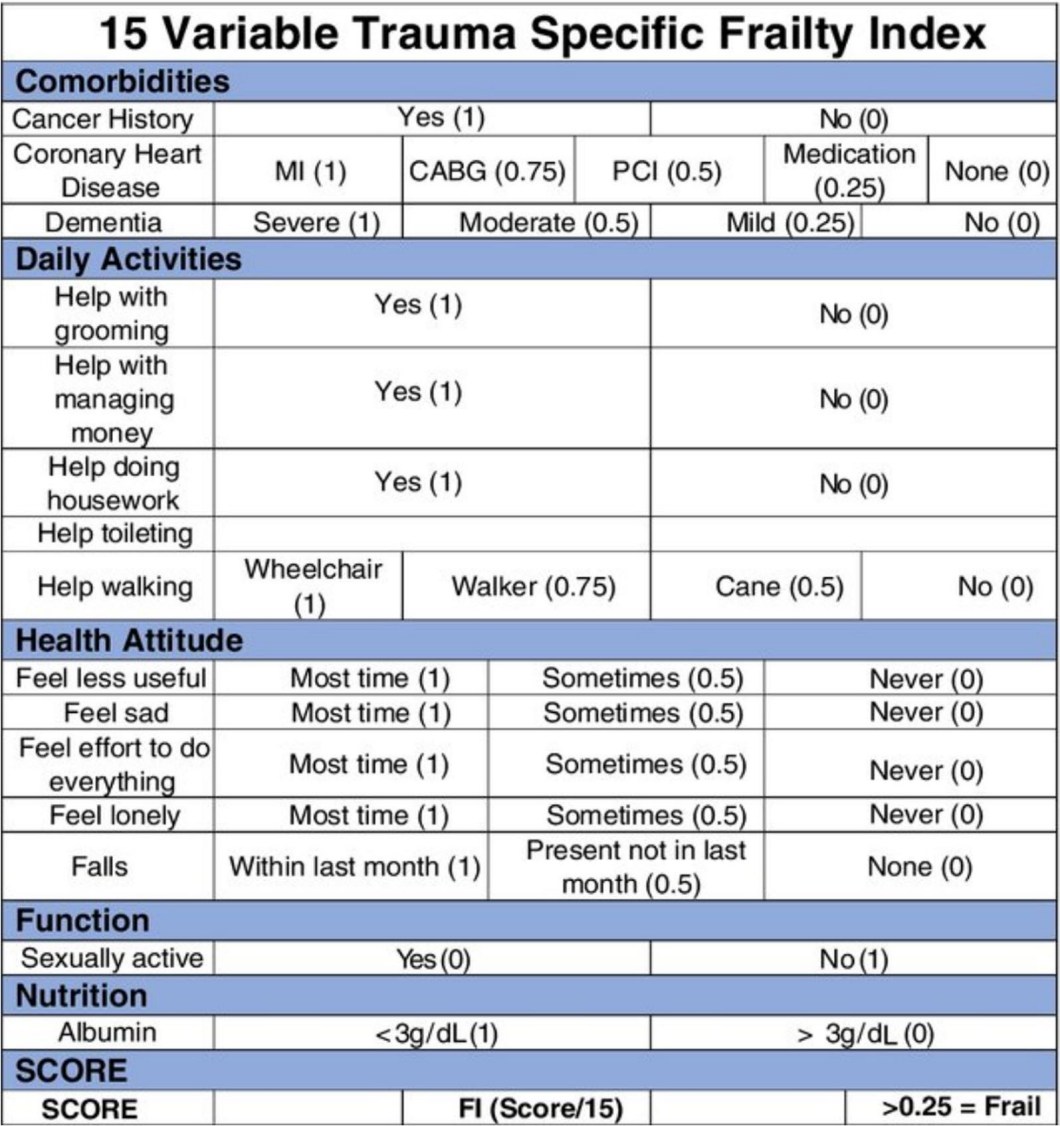
The Trauma-specific Frailty Index (TSFI)

The Trauma-specific Frailty Index (TSFI) **(**Fig. 2**)**, including frailty, is a modified 15-component scale validated in 200 patients; it has shown to be useful in planning discharge disposition of elderly trauma patients [67]. In a prospective cohort follow-up study conducted on 250 geriatric trauma patients at a Level I trauma center at the University of Arizona (the 44% of whom were classified as frail according to the TSFI), patients with frailty were more likely to have in-hospital complications (odds ratio, 2.5; 95% CI 1.5–6.0) and adverse discharge disposition (odds ratio, 1.6; 95% CI 1.1–2.4). The mortality rate was 2.0%, and all patients who died were frail [19]. Similarly, the Clinical Frailty Score (CFS) was found able to discriminate older patients at risk of higher mortality, delirium and increased care requirements at discharge. A large prospective study looking at frailty and trauma in older people in the UK have shown the CFS to be a useful tool to identify adverse post-injury outcomes in geriatric (≥ 65 years) trauma patients. This study showed that effect of frailty on mortality persists in less severe injury patterns with ISS ≤ 15. Frail patients had lower ISS (median 9 vs. 16) but greater 30-day mortality [68]. In keeping with these results, Cheung et al. performed a 4-year retrospective cohort study with 266 patients 65 years and older admitted to a level I trauma center, and found that pre-admission frailty as per the CFS (CFS 6 or 7) was independently associated with adverse discharge destination (odds ratio 5.1; 95% CI 2.0 to 13.2) [69].

The study by Hamidi et al. compared the predictive ability of different frailty scores to predict complications, mortality, discharge disposition, and 30-day readmission in trauma patients. The TSFI and the Rockwood Frailty Score (RFS) were found better predictors of outcomes compared with the modified Frailty Index (mFI) and the International Association of Nutrition and Aging 5-item a frailty scale (FS) [70].

Available data support the inclusion of a frailty assessment through the Trauma-Specific Frailty Index in the trauma evaluation for the geriatric population, to identify patients at highest risk of poor outcome.

**Key Question 2.2**

**Which clinical features do better define the hemodynamic instability in geriatric trauma patients?**

**Statement 2.2.1**

Most geriatric patients have hypertension, cardiovascular disease, and impaired sensitivity to catecholamines. They can be on chronic medications such as beta-blocker therapy that can affect heart rate and blood pressure, blunting the systemic response to injury and significant blood loss with the absence of early tachycardia (QoE B moderate).

**Statement 2.2.2**

Geriatric patients should have appropriate assessment of their poly-pharmacologic profile as soon as possible after admission. They should be screened for beta-blockers, steroids, antiplatelet and anticoagulant medications. The frequent use of anticoagulant (warfarin, coumadin, dabigatran, rivaroxaban) and antiplatelet (clopidogrel, aspirin) medications in the geriatric population, puts these patients at high risk for significant bleeding events, even after minor trauma (QoE B moderate).

**Recommendation 2.2**

We recommend keeping a lower threshold for trauma protocol activation in geriatric patients, with triage set points of heart ratio 90 bpm and systolic blood pressure less than 110 mmHg **[Strong Recommendation, based on Moderate Quality of Evidence, 1B].**

### Summary of evidence and discussion

Falls are the main cause of trauma in the geriatric population, accounting for 75% of cases, and are often low-level from standing or sitting height [16, 71–73].

Hashmi et al*.* investigated mortality rates in severe injured geriatric subjects aged ≥ 65 years and found that trauma patients aged ≥ 74 years were at a higher risk for mortality (overall mortality rate: 14.8%, 95% CI 9.8%-21.7%) than the younger geriatric group. Severe, extremely severe injuries, increasing age, and low systolic blood pressure at the presentation among geriatric trauma patients were found significant risk factors for mortality. Combined odds of dying in trauma patients older than 74 years was 1.67 (95% CI 1.34–2.08) compared with the elderly population aged 65 years to 74 years, but the odds of dying in patients 85 years and older compared with those of 75 years to 84 years was not different (odds ratio 1.23; 95% CI 0.99–1.52). A pooled mortality rate of 26.5% (95% CI 23.4–29.8%) was observed in the severely injured (ISS ≥ 16) geriatric trauma patients. Compared with those with mild or moderate injury, the odds of mortality in severe and extremely severe injuries were 9.5 (95% CI 6.3–14.5) and 52.3 (95% CI 32.0–85.5), respectively. Low systolic blood pressure had a pooled odd of 2.16 (95% CI 1.59–2.94) for mortality [74].

A systematic review and meta-analysis conducted by Sammy et al. in 2016 showed that trauma patients aged ≥ 75 had higher mortality rates than younger patients aged 65–74 years. Men had a significantly higher mortality rate than women (cumulative odds ratio 1.51, 95% CI 1.37–1.66), and patients with pre-existing comorbidity reported a higher risk of death. In particular, two studies that were evaluated in the systematic review reported increased mortality in patients on warfarin (cumulative odds ratio 1.32, 95% CI 1.05– 1.66). Higher mortality was found in patients with lower Glasgow Coma Scores and systolic blood pressures. Mortality increased with increased injury severity and number of injuries sustained. Low level falls were associated with higher mortality than motor vehicle collisions (cumulative odds ratio 2.88, 95% CI 1.26–6.60) [75].

Polypharmacy, defined as simultaneous co-administration of more than five medications, is often found in elderly people [76]. Fifty percent of geriatric patients have hypertension, 30% have heart disease, and 10% have diabetes, dementia, stroke, chronic pulmonary obstructive pulmonary disease, arrhythmias, or endocrine dysfunction [17].

The comorbidity–polypharmacy score (CPS) is able to quantify the magnitude of comorbid conditions using the number of co-administered medications as a measure of the “intensity” of therapy required for associated comorbidities [77]. Several studies have shown a negative association between polypharmacy and trauma outcomes, noting that higher CPS was associated with greater mortality, complications, longer hospital and intensive care unit stay, and need for discharge to a facility [77, 78].

Systolic blood pressure (SBP) and shock index (SI) are solid indicators of hemodynamic instability and the need for transfusion in the general trauma population [79, 80]. SI is also an accurate and specific predictor of morbidity and mortality in geriatric trauma patients. In the large study by Pandit et al*.* (217.190 geriatric trauma patients included), patients with SI greater than or equal to 1 were more likely to require blood products. Moreover, an SI greater than or equal to 1 was associated with the need of an exploratory laparotomy and the occurrences of in-hospital complications. The overall mortality rate was 4.1%, with an SI ≥ 1 being the strongest predictor for mortality (odds ratio, 3.1; 95% CI 2.6–3.3). With this in mind, geriatric trauma patients with SI ≥ 1 should be transferred to a Level 1 trauma center [81].

However, several studies have shown that SBP and SI cutoff points vary depending on the cause of trauma, pre-existing patient's illness, age, hypertension, and medication such as beta- or calcium channel blockers [78, 82, 83].

Park et al. retrospectively analyzed 4.681 trauma patients referred to a Level 1 trauma center between 2017 and 2018 with the aim to assess the utility and cutoff points of SBP and SI for predicting massive transfusion according to patients’ age and antihypertensives taking. There were 1.949 patients aged 65 years or older (41.6%), and 1.375 hypertensive patients (29.4%) in this study. Massive transfusion was given to 2.9% of patients, and 30-day mortality rate was 6.3%. In geriatric trauma patients taking antihypertensives, a prehospital SBP less than 110 mmHg was the cutoff value for predicting massive transfusion in multivariate analyses, whereas emergency department SI greater than 1.0 was the cutoff value for predicting massive transfusion in patients who were older than 65 years and were not taking antihypertensives [84].

Hemorrhage and hypoperfusion can be missed in this population because vital signs do not reflect shock response. Medication, such as beta-blockers and comorbidity including hepatic and renal impairment, previous or ongoing malignancy, and chronic steroid use, can further increase the mortality risk in geriatric trauma patients by up to five times [16]. Geriatric blunt trauma patients warrant increased vigilance despite normal vital signs on presentation. Triage set points of heart ratio 90 bpm should be considered in these patients, and lower threshold for trauma protocol activation is recommended, because in cases of under-triage of geriatric trauma patients, discharge disability and mortality rate are increased up to four times greater than younger adult patients [72].

The classic definition for hypotension in adults (90 mmHg) is linked to significantly greater mortality in the geriatric population [85].

The U.S. National Trauma Triage Protocol (NTTP) developed by the American College of Surgeons' Committee on Trauma and the Centers for Disease Control recognized that systolic blood pressure less than 110 mmHg may represent shock in patients older than 65 years [86]. Similarly, in a large retrospective cohort study on 902.852 trauma victims, Oyetunji et al. showed that optimal emergency department systolic blood pressure cutoff values for hypotension were 85 mmHg for patients aged 18–35 years, 96 mmHg for patients aged 36–64 years, and 117 mmHg for elderly patients [85].

Heffernan et al. performed a Level 1 trauma center retrospective chart review of heart rate and blood pressure at presentation in 2.081 young (aged 17–35 years) and 2.194 geriatric (aged 65 years or older) blunt trauma victims. They found that mortality increased considerably in the elderly patients for heart rates 90 bpm, whereas this association was not seen until hearth rate of 130 bpm in the young group. Moreover, mortality significantly increased with systolic blood pressure less than 110 mmHg in the geriatric patients, but not until a systolic blood pressure of 95 mmHg in the young patients [47].

Brown et al. evaluated the impact of substituting a SBP of less than 110 mmHg for the commonly recognized SBP of less than 90 mmHg criterion within the context of the triage protocol on triage performance and mortality in geriatric trauma patients. The study included 1.555.944 patients and demonstrated that a SBP < 110 mmHg had higher sensitivity but lower specificity in the geriatric cohort of patients (13 vs. 5%, 93 vs. 99%). The area under the curve was higher for SBP of less than 110 mmHg individually in both geriatric and adult cohorts. Within the NTTP, the area under the curve was similar for SBP of less than 110 mmHg and SBP of less than 90 mmHg in geriatric patients. Substituting SBP of less than 110 mmHg resulted in an under-triage reduction of 4.4% with an increase of overtriage of 4.3% in the geriatric cohort. In summary, this study demonstrated that implementing the SBP of less than 110 mmHg criterion in geriatric trauma patients results in discrimination as good as the current SBP of less than 90 mmHg criterion, but with superior improvements in under-triage relative to over-triage [87].

The implementation of these recommendations results in more timely care for geriatric patients and leads to faster mobilization of important resources in the emergency department.

**Key Question 2.3**

**Which laboratory tests and biological markers are useful to evaluate the elderly trauma patient before resuscitation?**

**Statement 2.3.1**

Occult hypoperfusion is often under-estimated in the geriatric trauma patient. A prompt assessment of base-deficit and lactates levels should be performed to identify those patients who need resuscitation and admission to an ICU. Elevated lactate and base deficit are definitely strong predictors of mortality within 24 h from hospital admission (QoE moderate).

**Recommendation 2.3**

We recommend performing an early blood gas (arterial or venous) for baseline base-deficit or a lactic acid assessment in geriatric trauma patients **[Strong Recommendation, based on Moderate Quality of Evidence, 1B]**.

### Summary of evidence and discussion

Decreased physiologic reserve results in relative intolerance to hypoperfusion and increased risk of multiorgan failure and death in the geriatric trauma patient [88].

Comorbidity and polypharmacy may mask the hemodynamic responses to hypovolemic shock. The Eastern Association for the Surgery of Trauma guidelines estimated under-triage rates of nearly 50% among geriatric trauma patients [57], this trend being likely because of the presence of occult hypoperfusion, and injuries associated with low-energy mechanisms of trauma. However, with a prompt recognition of the traumatic injuries, and aggressive resuscitation, up to 85% of geriatric trauma patients return to their pre-injury functional levels [89].

In hemodynamically stable elderly trauma patients, the identification and treatment of occult hypoperfusion are particularly challenging. Reliable triage tools for identifying the at-risk geriatric trauma patient are critical, as prolonged occult hypoperfusion in these patients increases mortality from 12% to nearly 35% [88]. In the general trauma population, lactate and base deficit are reliable markers of blood perfusion and have been shown to be highly sensitive in the identification of high-risk trauma patients [90, 91].

Schulman et al. evaluated the effects of prolonged occult hypoperfusion on mortality in 195 younger (mean 56 years) and 69 elderly (mean 55 years) blunt trauma patients. This study found that elevated arterial lactate at admission and prolonged clearance times were proxies for prolonged occult hypoperfusion and predicted increased ICU admission and overall mortality. Specifically, elderly trauma patients with admission arterial lactate greater than 2.4 mmol/L had mortality rates of 34.6% compared with 11.6% for patients with normal lactate. By comparison, patients less than 55 years of age with elevated lactate had mortality rates of 4.6% [88]. In the same line, compared with a hospital survival rate of 85% to 86% for elderly normotensive patients with normal blood base deficit or lactate concentration upon emergency department arrival, the results of the study by Callaway et al*.*, indicated a significantly decreased hospital survival rate of 60% associated with blood base deficit of 6 mEq/L or lactate concentration of 4 mmol/L upon hospital arrival. Mean lactate was significantly higher in non-survivors compared with survivors (2.8 mm/L ± 1.8 mm/L vs. 2.0 mm/L ± 1.0 mm/L). Patients in the severely elevated lactate group had 4.2 increased odds of death compared with the normal lactate group. Similarly, base deficit was more abnormal in non-survivors compared with survivors (2.3 mEq/L ± 5.2 mEq/L vs. 0.28 mEq/L ± 1.0 mEq/L). Normal, moderate, and severe base deficit were associated with mortality rates of 14% (95% CI 10.3–17.1%), 27% (95% CI 20.1–34.2%), and 40% (95% CI 24.9–54.1%), respectively [50]. Patients with a lactate 2.5 mmol or greater were 3.7 times more likely to die than those with a lactate less than 2.5 mmol (95% CI 1.6–8.2) in the study by Neville et al. The odds for mortality was 5.2 (95% CI 2.5–11.2) in patients with a base deficit of − 4 or less [92]. Early identification and treatment of occult hypoperfusion in geriatric patients with trauma using venous lactate-guided assessment and early trauma surgeon involvement is associated with significantly lower mortality [93].

Geriatric trauma patients have lower hemoglobin levels on admission, and persistently lower hemoglobin levels on discharge compared with younger trauma patients, despite they receive more blood transfusions [94], suggesting that aging may have a negative impact on post-injury anemia. Acute and subacute anemia is common among geriatric trauma patients, and they appear to respond less well to blood transfusion compared with young trauma patient. Moreover, as anticoagulant and antiplatelet medication use in trauma patients has been associated with increased risk of bleeding from minor injuries, elevated severity of injury, and increased mortality [6, 95].

In the study by Williams et al*.*, warfarin anticoagulation was associated with increased mortality after trauma in the geriatric patient (mortality for patients with an INR > 1.5 was 22.6%, versus 8.2% for those with an INR < 1.5). The logistic regression gave an age and ISS adjusted odds of death of 30% for a one-unit increase in INR (OR 1.3, 95% CI 1.1–1.5). This correlates to an age and injury score adjusted odds of death of 2.5 for an INR > 1.5 (95% CI 1.2–4.2). As elderly patients are commonly anticoagulated, considering the increasing number of indications for and prevalence of anticoagulation, the low cost of an INR dosage and the potential reduction in costs associated with traumatic brain injury, the assessment of a coagulation profile in elderly trauma patients is recommended to identify earlier those in need of closer monitoring and a more aggressive reversal of their anticoagulation [96].

Major trauma is also associated with a higher incidence of sepsis and multiple organ dysfunction, as a result of tissue damage, hypotension, hypoxia, cytokine release, and inflammation. Early identification of elderly patients at risk of developing post-traumatic complications is important for outcomes.

Al Rawahi et al. reviewed 19 observational studies that showed a strong correlation between initial procalcitonin levels and ISS. Twelve studies demonstrated significant elevation of initial procalcitonin levels in patients who later developed sepsis after trauma. Procalcitonin level was a strong predictor of multiorgan failure in seven studies, making it a promising as a surrogate biomarker for trauma [97]. Initial peak PCT level may be used as an early predictor of sepsis, multiorgan failure, and mortality in trauma patients.

In assessing hemostasis and bleeding disorders, TEG may have a role in managing elderly trauma patients. In the prospective observational study by Williams et al*.*, the correlation between conventional coagulation tests (INR an PTT), platelet function analysis (PFA) and TEG values was examined. INR and PTT correlated positively with TEG Reaction-time. However, TEG had a higher specificity, although non-significant (86.1%) in identifying hemorrhage progression compared with conventional coagulation tests (72.8%) and PFA (59.6%) [98, 99].

**Key Question 2.4**

**Which imaging studies are useful to better evaluate trauma elderly patients?**

**Statement 2.4.1**

Geriatric patients show injury patterns that differ considerably from those seen in the younger population. They are prone to serious injuries after relatively minor trauma because of overall frailty, comorbidity, and medication effects. Early diagnosis and aggressive intervention can decrease mortality and enable geriatric patients to return to independent living (QoE B moderate).

**Recommendation 2.4**

We recommend a low threshold for initial imaging with CT scan in geriatric trauma patients. The diagnostic yield of a contrast-enhanced CT outweighs the risk of contrast-induced nephropathy, especially in view of the potential, dramatic effects of under-triage **[Strong Recommendation, based on Moderate Quality of Evidence, 1B]**.

### Summary of evidence and discussion

CT is the primary imaging modality used in the setting of geriatric trauma [17, 72].

Intravenous contrast medium is used as part of CT standard protocols unless the patient reports documented evidence of a contrast allergy [100]. Although there is a higher prevalence of baseline renal impairment in elderly patients, there is no evidence that age is an independent risk factor for contrast-enhanced nephropathy [101]. Reason for which, the diagnostic yield of a contrast-enhanced study outweighs the risk of contrast-induced nephropathy, especially in view of the potential, dramatic effects of under-triage. In geriatric trauma patients with impaired renal function, it is important to follow age-appropriate guidelines for contrast agent administration in cases of minor trauma. However**,** in the setting of major trauma, intravenous contrast agent can be administered in patients with severe renal insufficiency (GFR < 30 mg/mL) to help diagnose life-threatening injuries at CT or before obtaining the serum creatinine concentration and estimating the GFR [72].

Head trauma in the geriatric patient carries a greater risk of intracranial injury irrespective of ISS, together with an increased mortality and morbidity (two-fold and four-fold, respectively) compared to the younger population. This is due to the increased risk of hemorrhage in all the intracranial compartments, but particularly the subdural compartment [72, 102]. Even in cases of serious intracranial injury, geriatric patients are less likely to manifest neurologic signs of raised intracranial pressure because of brain atrophy. It is estimated that around 3% of geriatric trauma patients have intracranial injury without clinical signs, history of loss of consciousness, focal neurology, or change in GCS [103]. Therefore, consensus documents currently recommend performing head CT scan in all geriatric patients with head injury, included those who report minor trauma [72, 101]. There is greater probability that geriatric patients with head trauma will return to independent living if early detection and treatment of traumatic injuries did occur and if they are treated promptly [103, 104]. Patients who are under anticoagulants, and those with hematologic conditions or liver disease are at increased risk for intracranial hemorrhage after trauma [105]. Aspirin and non-steroidal anti-inflammatory medication also cause coagulopathy. Prompt head CT of geriatric trauma patients who take anticoagulation medication should be carried out, even in those with minor head trauma. If CT images are positive for intracranial injury, coagulopathy should be reversed as soon as possible because the risk from head injury generally outweighs the benefit of anticoagulation therapy. If CT images are negative for hemorrhage the patient's coagulopathy does not require reversal, but the patient should be monitored closely within a protocol of 24-h of observation followed by repeat CT to identify occurrences of delayed bleeding [106]. The threshold for performing follow-up CT should be low in these patients if there is any evidence of clinical deterioration. Protocols suggest that all patients with traumatic intracranial hemorrhage who are treated conservatively undergo follow-up CT at 4–6 h, or earlier if there is clinical deterioration [72].

Poorer osseous mineralization, osteoporosis, and increased spinal rigidity put the elderly at a greater risk of having a spinal injury. Since 50% of geriatric cervical spine fractures are clinically unstable, and delayed diagnosis may result in secondary neurologic deterioration, prompt imaging is required. Geriatric patients are also more likely than younger patients to sustain multiple injuries. In these patients, the diagnostic value of radiographic assessment of cervical spine fracture is limited by reduced bone density and spondylotic changes. Therefore, injuries may be missed in up to 80% of elderly trauma patients at the radiographs [107–109].

Geriatric patients who sustain moderate to high-energy trauma, from both motor vehicle or fall from a height, and patients with focal neurologic signs, head injury, and associated injuries should be evaluated with cervical spine CT. Those who require head CT for trauma evaluation should also have concurrent screening CT of the cervical spine, since patients with an apparently isolated head injury have a 5% risk of additional spinal injury [109]. Screening CT of the cervical spine is also recommended in geriatric patients older than 75 years who sustain minor trauma, because of the high incidence of injury at C2 in this age group [108]. In summary, there should be a low threshold for imaging with CT scan (eventually associated with MRI) in these patients, given the complications that may arise from a missed cervical spinal fracture and the risk of spinal cord injury without radiological evidence of trauma (SCIWORET).

Cervical trauma may be also associated with blunt cerebrovascular injury. In patients with alarming clinical and radiological findings, including neurological deficits, GCS < 6, petrous bone fracture, foramen transverseria fracture, diffuse axonal injury, and a Le Fort II or III fracture, CT angiography is helpful in detecting any cerebrovascular injury [101]. According to the Denver criteria, CT angiography is recommended to screen for blunt cerebrovascular injury in patients with cervical spine fractures from C1 to C3, and traumatic cervical spine subluxations [110].

Osteoporosis is a major risk factor also for vertebral compression fractures in the thoracolumbar spine in the elderly population. The most common site of injury is at the thoracolumbar junction (T12–L2), followed by the mid-thoracic spine. Diagnosis of thoracolumbar osteoporotic compression fractures can be difficult at radiography as radiographs may not reveal an acute fracture line because of decreased bone mineral density [111]. If radiographs show loss of height in a vertebral body and the patient has focal pain, a CT scan should be performed to determine if the collapse is acute or chronic. CT achieve better diagnostic assessment of vertebral body height reduction and spinal canal diameter in cases of burst fractures with retropulsion. A CT scan of the whole spine is indicated in patients with major spinal trauma because 40% of injuries involve multiple, non-contiguous segments [72].

Age > 65 years, together with number of rib fractures (the more ribs that are fractured, the worse the outcome) and the ISS, is a well-recognized prognostic factor associated with morbidity and mortality in older adults with blunt chest trauma [112]. Similar to what happens for spine fractures, bone demineralitazion put the geriatric population at a high risk of rib fractures from lower energy trauma and at worse outcomes than do younger adults [113]. Rib fractures are also a potential sentinel injury associated with more severe trauma, including cardiac and aortic, pneumothorax, pulmonary contusion and laceration, as well as liver and splenic trauma. Similarly, clavicular and first rib fractures are sentinel injuries for severe thoracic and brain injuries and are associated with a higher mortality rate in the elderly [56, 72]. The radiologist can identify patients with rib injuries and flail chest segment that may benefit from open reduction and internal fixation, in order to enable patients to commence physiotherapy earlier, and decreasing the risk of secondary chest infections [114].

Chest radiographs fail to detect approximately 50% of rib fractures visible at CT [115].

Several studies have reported the low sensitivity of chest X-ray for traumatic pneumothorax and hemothorax [116, 117]. The image quality may be also worst in geriatric trauma patients with lack of decubitus capability, and when using portable devices. Failure to detect these injuries at radiographs is a clinically relevant issue, as three or more traumatic rib fractures in patients aged 65 years or older, if associated with a history of chronic obstructive pulmonary disease or congestive heart failure, substantial pain, mental status changes, pulmonary contusion or laceration, hemothorax or pneumothorax, flail chest and abnormal oxygenation or ventilation mandate ICU admission and observation.

The extended Focused Assessment with Sonography in Trauma (eFAST) exam is another accepted part of the trauma evaluation nowadays, and can be implemented to identify pneumothorax, pericardial effusions, and intra-abdominal free fluid. Early detection of these findings can guide the prioritization of the performance of further diagnostic and therapeutic interventions. The systematic review and meta-analysis by Netherton et al. suggested that the e-FAST is a useful tool for ruling in pneumothorax, pericardial effusion, and intra-abdominal free fluid in the trauma setting. Pooled sensitivities and specificities were 69% and 99%, respectively, for the detection of pneumothorax (area under the curve 0.994), 91% and 94% for pericardial effusion (area under the curve 0.975), and 74% and 98% (area under the curve 0.888) for intra-abdominal free fluid [118]. Recently, a Cochrane review of 13 studies (410 traumatic pneumothorax patients out of 1.271 patients) compared the diagnostic accuracy of chest ultrasonography by frontline non-radiologist physicians versus chest X-ray for diagnosis of pneumothorax in trauma patients in the emergency department. The summary sensitivity and specificity of chest ultrasound were 0.91 (95% CI 0.85–0.94) and 0.99 (95% CI 0.97–1.00); and the summary sensitivity and specificity of supine chest X-ray were 0.47 (95% CI 0.31–0.63) and 1.00 (95% CI 0.97–1.00). There was a significant difference in the sensitivity of chest ultrasonography compared to chest X-ray, with an absolute difference in sensitivity of 0.44 (95% CI 0.27–0.61), whereas the two imaging tools had similar specificities. These findings suggested that chest ultrasonography for the diagnosis of traumatic pneumothorax could be incorporated into trauma protocols and algorithms [119]. Similarly, another Cochrane review, published by Stengel et al. in 2018, demonstrated that, in patients with suspected blunt thoracoabdominal trauma, positive point-of-care ultrasound findings are helpful for guiding treatment decisions in chest injuries, whereas, with regard to abdominal trauma, a negative point-of-care ultrasound exam does not rule out injuries and must be verified by CT scanning. The review included 34 studies with 8.635 participants. Summary estimates of sensitivity and specificity were 0.74 (95% CI 0.65–0.81) and 0.96 (95% CI 0.94–0.98). Pooled positive and negative likelihood ratios were estimated at 18.5 (95% CI 10.8–40.5) and 0.27 (95% CI 0.19–0.37), respectively. The reported accuracy of point-of-care ultrasonography in the adult population was 0.78 (95% CI 0.69–0.84), and associated specificity was 0.97 (95% CI 0.96–0.99). For abdominal trauma, ultrasonography had a sensitivity of 0.68 (95% CI 0.59–0.75) and a specificity of 0.95 (95% CI 0.92–0.97). For chest injuries, sensitivity and specificity were calculated at 0.96 (95% CI 0.88–0.99) and 0.99 (95% CI 0.97–1.00) [120]. Similarly, the systematic review and meta-analysis by Staub et al. suggested that chest ultrasonography is an accurate tool for the diagnostic evaluation of traumatic pneumothorax and hemothorax in adults. Nineteen studies were included in the review, 17 assessing pneumothorax and 5 assessing hemothorax. The reference standard was chest CT scanning alone, or in parallel with chest radiography and observation of the chest tube. The diagnostic accuracy of chest ultrasonography showed an area under the curve of 0.979 for pneumothorax. The absence of lung sliding and comet-tail artifacts were the most reported sonographic sign of pneumothorax, with a sensitivity of 0.81 (95% CI 0.71–0.88), and specificity of 0.98 (95% CI 0.97–0.99). An echo-poor or anechoic area in the pleural space was the only sonographic sign for hemothorax, with a sensitivity of 0.60 (95% CI 0.31–0.86), specificity of 0.98 (95% CI 0.94–0.99), and area under the curve of 0.953 [121].

Blunt abdominal trauma is uncommon in geriatric patients after a ground-level fall, unless the patient has a preexisting condition such as coagulopathy. However, when abdominal trauma does occur, this is related with a five-fold increase in the mortality rate when compared to younger patients [72]. As clinical diagnosis of abdominal injuries is more challenging in the elderly than in the younger population, it is important to have a lower threshold for CT to diagnose intra-abdominal injuries in geriatric patients [101]. This decreases the duration of hospital stay, ICU admission rates, mortality, and morbidity even in cases of high ISS [122]. Moreover, Arruzza et al. demonstrated that whole-body CT as part of the trauma primary survey, in comparison to other conventional radiologic procedures, shortens time spent in the emergency department [123]. These findings have relevant implications, entailing faster diagnosis time for definitive treatment and lessening the impact of emergency department overcrowding.

Approximately one in ten of admitted blunt trauma patients in trauma referral centers sustain pelvic fractures [124]. Bleeding pelvic fractures are an immediate life-threatening injury, but early invasive monitoring, intervention with angiography, and prompt hemorrhage control are associated with improved survival [125, 126]. Contrast enhanced CT is the mainstay screening imaging for evidence of arterial bleeding in patients with pelvic fractures, and contrast extravasation is the most reliable predictor of the need for pelvic angiography and Trans-Arterial Embolization (TAE), regardless of hemodynamic status [127].

Surgical decision making remains challenging due to difficulty of determining the bleeding source. TAE and external fixation are the most common treatment strategies for hemorrhage associated with pelvic fractures, with early TAE aimed at establishing an effective means of reducing transfusion requirement, complications, and mortality from arterial hemorrhage [127, 128], whereas low pressure bleeding from the pelvic venous plexus or fractured bone ends is best controlled through splinting, reduction of pelvic volume, and tamponade using external fixation [125, 129].

## Resuscitation

**Key Question 3.1**

**What early resuscitative protocol including intravenous fluids, blood transfusions or vasopressors should be used to manage geriatric trauma patients at primary evaluation?**

**Statement 3.1.1**

Available data do not recommend a specific early resuscitative protocol over another in geriatric trauma management. (QoE D very low).

**Statement 3.1.2**

Resuscitative protocols for elderly trauma patients aim to early identification of tissue hypoperfusion, and rapid treatment of coagulopathy, hypovolemia, and traumatic injury to improve outcomes and decrease mortality (QoE B moderate).

**Statement 3.1.3**

In the elderly trauma patient, the resuscitative strategy should be individualized and tailored according to clinical history, comorbidities, concomitant medications, clinical and laboratory findings, and treatment response. (QoE B moderate).

**Statements 3.1.4**

In elderly trauma patient, close monitoring and frequent repeated measurements of vital signs trend and gas analysis are likely to be more useful than any individual measurement to guide the resuscitative strategy (QoE C low).

**Recommendation 3.1**

We recommend that every trauma center provides meticulous triage criteria to recognize the need to early activate resuscitative protocols for elderly patients. These triaging criteria should include physical examination, vital signs, blood gas analysis, and medical history, emphasizing clinical conditions and drug history that may guide resuscitative therapies, early coagulative support, and the need to correct coagulopathies, and minimise fluids **[Strong recommendation based on moderate quality of evidence 1B].**

We recommend rapid recognition and correction of coagulation disorders related to trauma or chronic medication intake in elderly patients**. [Strong recommendation based on moderate quality of evidence 1B].**

We recommend performing serial base deficit assessment and lactate levels as markers of occult hypoperfusion in addition to close monitoring of vital parameters trend (heart rate, blood pressure, respiratory rate, urinary output), and mental status in elderly patients in a dedicated intensive geriatric care unit **[Strong recommendation based on moderate-low level quality of evidence 1B]**.

We suggest considering carefully to administer inotropic agents in selected non-responding elderly patients to target resuscitation **[Weak recommendation based on low level of evidence 2C].**

### Summary of evidence and discussion

The reliability of vital signs assessment alone is not sufficient to guide the management of geriatric patients after trauma. Personalized evaluation of hemodynamic stability is crucial to establish a tailored resuscitation [75, 130]. A retrospective study reported that mortality increases among older trauma patients when their heart rate rises above 90 beats per minute and systolic blood pressure falls below 110 mmHg, while the same increase in mortality is not evident in younger patients until heart rates reach 130 beats per minute and systolic blood pressure falls below 95 mmHg [47].

Another study reported evidence of tissue hypoperfusion despite "normal" blood pressure in older adult trauma patients without isolated head injury [54]. Around one-third of elderly trauma patients show chronic signs of tissue hypoperfusion (measured by lactates and base excess) with threshold systolic blood pressure values adopted for other types of patients [95].

Physiological response to shock is different in geriatric patients, and standard alarm vital signs such as tachycardia and hypotension with systolic blood pressure less than 80 mmHg can be absent. In fact more than 50% of the geriatric trauma patients has underlying hypertension, and more than 30% has heart disease treated with medications [131]. Moreover geriatric patients have altered cardiovascular physiology, with cardiac function declining by 50% between 20 and 80 years of age [6].

It is crucial to recognise the effect of medications and polypharmacy which are used to treat hypertension, diabetes, previous cerebrovascular events, chronic obstructive pulmonary disease (COPD), dementia, arrhythmias, endocrine disorders and chronic renal failure which may obscure vital sign parameters. Beta blockers and other antihypertensive medications, eventually associated with a pacemaker in place, can blunt the normal tachycardic compensatory response for improving cardiac output in class II hemorrhagic shock. Furthermore, tachycardia response could be reduced by the decreased sensitivity of aging myocardium to circulating catecholamines limiting increasing cardiac output via stroke volume [6–17]. Because of these mechanisms, geriatric patients compensate by increasing systemic vascular resistance, resulting in a deceptively acceptable blood pressure [6–79]. Geriatric patients are frequently treated with anticoagulants and antiplatelet agents mainly because of cardiovascular diseases and atrial fibrillation (FA); this puts geriatric trauma patients at risk of severe bleeding from apparently mild wounds or after ground-floor fall with missed head trauma leading to poor outcomes [6–104]. The administration of steroids prescribed for COPD in the elderly can reduce wound healing and lead to clinical adrenal insufficiency in critically ill patients. Steroid use can independently increase mortality up to fivefold in the geriatric trauma population [6–104]. Antipsychotics and antidopaminergic agents for Parkinson disease make the neurologic examination unreliable. Glaucoma treatment may alter the pupillary examination and consequently the GCS score [6–48].

This makes the assessment of injury severity and hemodynamic instability in geriatric patients depending only on clinical evaluation so as to timely activate resuscitative protocols very challenging.

An aggressive triage with rapid trauma team activation, early recognition, and treatment of hypoperfusion and coagulopathy may improve outcomes [132, 133].

Bradburn et al. showed that adopting rapidly high-risk geriatric trauma protocols, including early consultation by a geriatrician, measuring lactates and arterial blood gases, and point-of-care ultrasound to assess occult peripheral hypoperfusion, can decrease mortality [132].

Given that vital signs may be unreliable to guide the assessment of hemodynamic status in a geriatric patient, it is important to look for different signs of shock in patients who are normotensive and do not have tachycardia. Signs such as mild confusion, somnolence, or agitation, mild tachypnea, delayed capillary refill, and low urine output may reflect tissue hypoperfusion and early shock [47–54].

The early management of hypotensive geriatric patients is comparable to that of adults and hypotension should be considered hypovolemic until proven otherwise. The early resuscitation includes restrictive volemic replacement with balanced crystalloids. In case of failure to respond, it is indicated to start a volemic restoration with blood products to reduce the possibility of the onset of Trauma Induced Coagulopathy (TIC) aiming at replacing the whole blood [27, 28].

The prompt recognition of the need for massive transfusion (MT) is essential in geriatric trauma patients with different thresholds for systolic blood pressure (SBP) (< 90, < 100 or < 110 mmHg) depending on the cause/mechanism of trauma, comorbidities, age and polypharmacy [133].

The shock index (SI), which is the ratio of heart rate (HR) to SBP, was reported to be an accurate indicator of hemodynamic instability and the need for transfusion in trauma patients [133].

Risk factors to predict the need for MT in elderly patients are related to Focused Assessment for Sonography results, unstable pelvic fracture, and long bone open fracture of the lower limbs, along with pre-injury anticoagulants use, anti-platelet agent use, lactate levels, and shock index [133].

A retrospective study assessing the cutoff for SBP and SI for predicting MT in geriatric trauma patients taking antihypertensives, showed that a pre-hospital SBP less than 110 mmHg was the cutoff value for predicting MT and that packed red blood cell transfusion volume decreased based on prehospital SBP of 110 mmHg. At the ED, SI greater than 1.0 was the cutoff value for predicting MT in patients who were older than 65 years and were not taking antihypertensives [84].

In practice, elderly patients have poor tolerance to multiple injuries due to weak resistance and body function decline. They are significantly more likely to receive a blood transfusion, specifically for red cells and plasma. However, such patients may suffer coagulation disorders due to the release of coagulation factors after blood transfusion, which increases the risk of organ failure, the main cause of late death in trauma patients [134]. In those cases, the use of prothrombin complex concentrates (PCC), with or without fresh frozen plasma (FFP) to correct the initial coagulation disorder has been described. PCC is effective in normalizing prothrombin time, bleeding time, peak thrombin generation and overall control of bleeding [135].

Mador et al. retrospectively studied 142 elderly (aged > 65 yrs) trauma patients compared with young patients were more likely to be female (41% vs. 24%), suffer blunt trauma (96% vs. 80%), have higher ISS scores (mean 25.4 vs. 21.6) and mortality (19% vs. 8%). They were significantly more likely to receive blood transfusion (42% vs. 30%), specifically for red cells and plasma [136].

Simon et al. [137] showed that liberalized transfusions, that is a strategy where transfusions are allowed as soon as hemoglobin (Hb) is ≤ 10g/dL, with a target Hb of ≥ 10g/dL, in the elderly were better than restrictive policies, which provides transfusion when Hb is ≤ 8g/dL, with a target Hb of 8–10 g/dL. Postoperative anemia is poorly tolerated by geriatric patients. The use of “old blood”, transfused more than 14 days after collection, versus “new blood”, transfused within 14 days from collection, is better in geriatric patients due to the presence of storage changing such as enhanced clearance, plasma transferrin saturation, nitric oxide scavenging and immunomodulation with potential harmful effects such as lung injury [138].

Early MT (10 packed red blood cells (PRBCs) units/24h) is the main treatment for patients presenting with severe multiple injuries associated with massive bleeding so as to improve the microcirculation, maintain blood volume, prevent hypotension-induced shock, replenish various coagulation factors and correct acute hypoxia [139].

Li et al. [140] reported that MT protocol for elderly patients with multiple injuries can improve coagulation function and platelet parameters, alleviate organ dysfunction, shorten length of ICU stay, and decrease the incidence of complications.

Initial volemic restoration should be guided by standard laboratory tests and Point of care Viscoelastic testing. Thromboelastography (TEG) monitors the dynamic changes of blood clot formation and lysis, and has been implemented in trauma to diagnose acute trauma coagulopathy, to assess expeditiously the level of coagulation factors, the function of fibrinogen and platelet, and the presence or absence of hyperfibrinolysis. TEG may guide transfusion practices and help identify patients with platelet function abnormalities requiring reversal. Nevertheless, there are concerns about values of reference according to age [141–144]. Scarpelini et al. reported that TEG values in healthy volunteers did not differ between the young and old, but most values were significantly different from those of the manufacturer having only 81% specificity. Healthy women were significantly more hypercoagulable than men. Aging was not associated with hypercoagulability [141].

Roeloffzen et al. showed that baseline TEG values may vary with age, elderly patients were more hypercoagulable [142].

Age-based differences in TEG has been also investigated in the peri-operative period among geriatric patients showing conflicting data about hypocoagulable and hypercoagulable status [143].

Mador et al. showed that trauma induced coagulopathy, as measured by TEG, was less commonly observed in the elderly. This suggests that altered coagulopathic response to traumatic injury is partially influenced by increased anticoagulant and antiplatelet medication use in the geriatric population [136].

In clinical practice, patients who had TEG analysis were more likely to receive platelet reversal agents, regardless of antiplatelet medication usage. Geriatrics seem to be less susceptible to alterations in TEG and therefore trauma-induced coagulopathy [143, 144]. In the lack of standardised TEG values of references and validation studies, the correction of TEG coagulopathy in geriatric trauma patients should be conducted carefully. In case of failure to respond to volemic restoration, initiation of vasopressor therapy can be considered until adequate perfusion is restored. In this setting, it is essential to consider other possible causes of shock (neurogenic, septic, obstructive, cardiogenic) especially in patients with suspected or apparent traumatic brain injury [27, 28]. An inotropic agent (dobutamine or epinephrine) may be considered in patients presenting with cardiac dysfunctions [145].

**Key Question 3.2**

**Which are the resuscitation endpoints in elderly trauma patients?**

**Statement 3.2.1**

In the elderly trauma patient, normotension and the absence of tachycardia and tachypnea do not.

Rule out tissue hypoperfusion. (QoE A-B strong-moderate).

**Statement 3.2.2**

There is no evidence that one type of invasive hemodynamic monitoring is more efficient than another in elderly trauma management; the indication for hemodynamic monitoring should be evaluated according to the patient's clinical features and the team's expertise. (QoE B-C moderate-low).

**Statement 3.2.3**

The adoption of Point-of-Care Ultrasound (POCUS) in the resuscitation of the elderly severely injured patient may be an effective tool in monitoring the hemodynamic status of the patient as it provides information on blood volume and cardiac function in a rapid, cost-effective manner, without the side effects of invasive monitoring systems. (QoE B-C moderate-low).

**Recommendation 3.2**

We recommend evaluating the indication for invasive versus non-invasive hemodynamic monitoring on a case-by-case basis in injured elderly patients. Hypoperfusion should be ruled out by serial base deficit assessments and lactate concentration** [Strong recommendation based on moderate-low level of evidence 1B].**

We suggest the implementation of POCUS in monitoring the cardiac function and blood volume in elderly injured patient, if skills are present. Invasive hemodynamic monitoring should be reserved in selected cases, to critically ill elderly trauma patients who have hypotension, significant injuries (as defined by an Abbreviated Injury Score > 3 or a Trauma Score < 15), or uncertain cardiovascular and/or fluid status **[Weak recommendation based on moderate and low level of evidence 2B].**

### Summary of evidence and discussion

Resuscitation must be started rapidly even in (apparently) stable elderly patients with close monitoring in the ICU. Renal function and urine output are usually considered as a marker of resuscitation. Nevertheless, renal function is decreased, in the elderly because of chronic decreased renal blood flow and declining renal mass. Creatinine clearance value needs to be adjusted to the elderly patient muscle mass which is reduced. Tachypnea could be absent because ventilatory mechanics of the elderly differ significantly from the younger patients. Elderly patients may have a normal respiratory rate although becoming progressively hypoxic and hypercarbic [146].

Invasive hemodynamic monitoring techniques were reported to be useful in high-risk selected geriatric trauma patients with occult hypoperfusion. A prospective randomized study on elderly patients with hip fractures showed that invasive monitoring with pulmonary artery catheters was associated with a significantly reduced mortality rate when compared with only a central venous pressure catheter [147].

Scalea et al. [20] reported the significant difference in cardiac output and peripheral vascular resistance between elderly trauma survivors and nonsurvivors. In their study, pulmonary artery catheters were used to guide resuscitation to a cardiac index of 4 L/min per m^2^ or an oxygen consumption of 170 mL /min per m^2^. The authors noted that the limited compensatory mechanisms of elderly patients might lead to the missed diagnosis of a perfusion deficit due to a decreased cardiac output.

Laboratory data used to estimate the acidemia (base deficit and lactates level) caused by perfusion deficits may help identify high-risk patients who may benefit from invasive monitoring with pulmonary artery catheters. Base deficit and lactates levels and their trend in time are an easily measurable surrogate of the mismatch between oxygen delivery and oxygen consumption, the consequent tissue hypoxia, and the increase of aerobic metabolism. Bar-Or et. showed that a resuscitative protocol based on lactate measurement helps recognize occult hypoperfusion and reduce mortality [93]. Callaway et al. found an association between base deficit, lactates, and mortality in a population of normotensive elderly trauma patients [50]. The presence of an increased base deficit (≤ − 6 mEq/L) on arterial blood gas sampling is associated with an increased mortality [148]. An elevated serum lactate level is a marker of occult hypoperfusion and the rate of clearance directly correlates with mortality [149]. The presence of a lactic acidemia level of more than 22 mg/dL (> 2.4 mmol/L) for longer than 12 h is associated with an increased mortality in geriatric patients [88]. Prompt normalization of the base deficit and serum lactate level are thought to be appropriate end-points in trauma resuscitation [150]. Elderly patients should be resuscitated with fluid and supported with pressor medications, as needed, to maintain a cardiac index of at least 4 L /min per m^2^ or an oxygen consumption of 170 mL /min per m^2^. The use of pulmonary artery catheters in high risk patients presenting with hypotension, significant injuries (as defined by an Abbreviated Injury Score > 3 or a Trauma Score < 15), or have uncertain cardiovascular and/or fluid status is a good tool to guide and monitor resuscitation [151, 152].

Point-of-Care Ultrasound (POCUS) can help evaluate cardiac function and volume status in unstable and traumatized patients [153].

Cleveland et al. showed that the use of POCUS can guide to resuscitation in an elderly trauma patients significantly reducing volumes of intravenous fluids and mechanical ventilation days [154].

**Key Question 3.3**

**Which vasopressors are indicated in comorbid elderly injured patients?**

**Statement 3.3.1**

The use of a vasopressors before volume replacement may be deleterious in all trauma patients (QoE A strong).

**Statement 3.3.2**

The use of vasopressors is indicated in trauma patients who do not respond to early fluids in the context of damage control resuscitation and permissive hypotension (QoE B moderate).

**Statement 3.3.3**

In trauma patients not responders to early resuscitation with hypotension refractory to volume filling, and with hypotension of neurogenic and septic origin, the vasopressor of choice is norepinephrine (QoE A strong).

**Statement 3.3.4**

In an elderly trauma patients, it is appropriate to consider the administration of an inotrope in case of non-response or in case of hypotension due to cardiac dysfunction (QoE C low).

**Statement 3.3.5**

Dobutamine may be helpful in selected elderly trauma patients presenting with shock-related to heart failure, bradycardia from cervicothoracic myelic injury, and cardiac contusion (QoE B-C moderate-low).

**Recommendation 3.3**

We recommend against the routine use of vasopressors in elderly injured patients presenting with hypotension caused by hemorrhage **[Strong recommendation based on high-moderate level of evidence 1A].**

We recommend identifying the cause of hypoperfusion and assessing preexisting conditions and pharmacologic history before choosing a vasopressor in managing trauma in an elderly patient **[Strong recommendation based on a high-low quality level of evidence 1A].**

We suggest using norepinephrine in elderly patients suffering from neurogenic shock. The dose to be used must be the lowest to guarantee tissue perfusion. The possible onset of cardiac arrhythmia and possible hypotensive effects should be monitored **[Weak recommendation based on a moderate-low quality level of evidence 2B].**

### Summary of evidence and discussion

The use of vasopressors in geriatric trauma patients is an ongoing debate. Vasopressors, such as norepinephrine and epinephrine, are commonly used in the management of hypotension and shock in critically ill patients, including trauma patients. However, their use depends on the origin of hypoperfusion that can be hemorrhagic, obstructive, cardiogenic, neurogenic, or septic. Each cause requires a different treatment. Vasodilation is a common manifestation of the various forms of shock after traumatic injury. While initial vasoconstriction is an early characteristic of hemorrhage (i.e., sympathoexcitatory phase), continued blood loss with subsequent hypotension may cause vasodilation. Vasodilatory shock is the most common form of shock and represents the final common pathway for severe shock from any cause. Handling of shock should be individualized based on underlying cause. Vasopressors are required only under specific conditions and under close monitoring [155]. The implementation of vasopressors in elderly trauma patients depends on factors such as comorbidities, frailty, and medication interactions. Some studies have suggested that their use in elderly trauma patients may be associated with increased mortality [156–164]. The concerns about vasopressor use in trauma patients include rapid increases in arterial blood pressure, increased cardiac afterload, arrhythmias, and reduced tissue perfusion with subsequent organ dysfunction [157, 158].

A retrospective study enrolling 255 trauma patients who had emergency surgery and received vasopressors during surgeryshowed that these patients were older, more severely injured, had worse vital signs, and increased mortality (all *P* < 0.001). Epinephrine was independently associated with increased mortality (odds ratio, 6.88; *P* = 0.001) [156].

A prospective observational study showed that older patients had high plasma noradrenaline, attenuated adrenaline release with higher Injury Severity, impaired platelet and leukocyte mobilization, enhanced consumption of anticoagulants, and hyperfibrinolysis compared to younger patients [157].

These biological status of the elderly could be correlated with the negative outcomes following the administration of vasopressors but this has to be more investigated. Moreover, the use of vasopressors in the resuscitation of massively transfused trauma patients might be considered a marker of inadequate resuscitation [158]. The immediate management goals in hemorrhagic shock should be mechanical control of bleeding, treatment of trauma-induced coagulopathy, and restoration of intravascular volume. If bleeding cannot be controlled immediately, then the management goal should be to minimize further blood loss until hemorrhage control can be achieved [27, 28].

Uchida et al. demonstrated in a retrospective single centre study that non-survivors trauma patients were administered significantly earlier vasopressors and significantly higher doses despite their similar characteristics and injury severity to those who survived. Max catecholamine index was significantly higher in non-survivors (2 [0–4] vs 14 [10–18]; *P* = 0.008). Administered vasopressors were terminated significantly earlier (12 [4–26] vs 34 [10–74] hours; *P* = 0.026) in survivors. Total blood transfusion within 24 h after admission was significantly higher in survivors (8430 [5680–9320] vs 6540 [4550–7880] mL; *P* = 0.03) [158].

Vasopressor administration and their high-dose use for resuscitation of traumatic hemorrhagic shock patients are associated with increased mortality. Early termination of vasopressors has to be considered in these patients although transfused volume of blood products may increase [158]

Singer et al. investigated the relationship between the maximum dose of norepinephrine, timing of norepinephrine administration, and mortality in trauma patients.Patients who died received an average maximum dose of 16.7 mcg/min compared to 9.1 mcg/min in survivors (*P* = 0.0003). Mortality rate increased with dosage (*P* < 0.0001), with doses greater than 20 mcg/min having 79% mortality. Those who received norepinephrine within the first 24 h had an inflection point in mortality at 16 mcg/min (Youden = 0.45) (OR 1.06; 95% CI 1.03–1.10). For patients who received norepinephrine after the first 24 h, an inflection point in mortality was at 10 mcg/min (Youden = 0.34) (OR 1.09; 95% CI 1.04–1.14) [159].

Cardinale et al. investigated the impact of norepinephrine dose during damage control resuscitation High doses of norepinephrine infusion were associated with deleterious effects as attested by a higher SOFA score at 24 h, and likely hypovolemia as measured by reduced non-blood resuscitation volume in trauma patients with hemorrhagic shock [160].

In contrast several studies showed that the early administration of vasopressors does not increase mortality [161, 162].

A retrospective, propensity score–matched cohort study reported no significant increase with in-hospital mortality in patients who received prehospital norepinephine [163].

A retrospective study of 746 trauma patients requiring emergent operations observed no significant increase in mortality in patients who received vasopressors, exclusive of epinephrine [156].

Two RCTs investigated the role of arginine vasopressin (AVP) in managing resuscitation and hypotension. AVP activates vascular smooth muscle V1 receptors independent of α-adrenergic stimulation, mitigates vasoplegia and increases venous capacitance which is observed in late-stage shock by inhibiting vascular adenosine triphosphate–sensitive potassium channels and by blunting nitric oxide–induced vasodilation [164, 165]. Cohn et al. assessed the safety and efficacy of adding AVP to resuscitative fluid. The authors blindly randomised 78 hypotensive patients with acute traumatic injury to fluid alone group (control) and fluids + vasopressin (experimental). The experimental group required a significantly lower total volume of resuscitation fluid over 5 days than did the control group (*P* = 0.04). The mortality rate at 5 days was 13% in the experimental group and 25% in the control group (*P* = 0.19) [164].

Sims et al. investigated whether low-dose AVP supplementation decreased the need for blood product transfusions in patients with traumatic hemorrhagic shock. Using low-dose AVP supplementation in patients presenting significantly decreased the need for blood products without increasing morbidity [165]. All these studies were carried out on general population, without distinction of age.

In general, fluid resuscitation is the first-line therapy to restore intravascular volume and to prevent cardiac arrest. At this point of time, before source bleeding control, the main endpoint is to maintain arterial pressure to the bare minimum to minimize dilution of coagulation factors and complications of over fluid resuscitation, considering the severity of the hemorrhage. A target systolic arterial pressure of 80–90 mmHg is recommended until the control of hemorrhage in trauma patients without brain injury. Early vasopressor support may be required to restore arterial pressure and prevent excessive fluid resuscitation. It is crucial to find the best harmony between fluid resuscitation and vasopressors, to consider hemodynamic monitoring and to establish trauma resuscitative protocols [166]. The administration of vasopressors to manage hypotension has to be decided in a case by case basis and carefully monitored by the trauma team.

Clinicians give vasopressors to avoid hypotension which can be exacerbated in hemorrhagic shock due to vasodilatation; however, excessive vasoconstriction and other effects associated with vasopressors—such as increased cardiac workload—could cause harm to critically ill patients. Invasive pressure monitoring is indicated in all patients receiving continuous infusion of vasopressors. The choice of vasopressor depends on the preexisting condition of the patient and the pathophysiology of each case of hypoperfusion. Invasive and non-invasive hemodynamic monitoring can aid the choice of vasopressor and its titration of an appropriate dosage [167, 168].

There is no specific literature on vasopressors in the elderly population. Based on the evidence on general polytrauma patients, the use of vasopressors can be recommended in patients who do not respond to volemic filling in the context of damage control resuscitation and permissive hypotension. The vasopressor of choice is norepinephrine, which can be used in patients with hypotension refractory to volemic filling and patients with hypotension of neurogenic and septic origin. It is appropriate to consider the addition of an inotrope in case of nonresponse or in case of hypotension due to cardiac dysfunction. Focused resuscitative protocols tailored to geriatric trauma patients are lacking, and further prospective studies on this topic are needed.

**Key Question 3.4**

**Vasopressors treatments versus permissive hypotension in geriatric trauma patients: which are the clinical parameters and laboratory tests to consider for choice?**

**Statement 3.4.1**

The administration of vasopressors versus permissive hypotension debate during the early resuscitative stage of elderly trauma patients remains unresolved. Management should be individualized according to the mechanism of trauma, the patient's acute and chronic conditions, and frailty (QoE B-C moderate-low).

**Recommendation 3.4**

We recommend to carefully evaluate to implement permissive hypotension in managing selected elderly trauma patients. Tissue perfusion has to be constantly monitored by base excess level, arterial lactate dosage, urine output, and when possible, neurologic assessment. **[Strong recommendation based on a high-low quality level of evidence 1A].**

### Summary of evidence and discussion

The concept of “permissive hypotension” refers to managing trauma patients by restricting the amount of fluid resuscitation administered while maintaining blood pressure lower than normal if there is still active bleeding during the acute period of injury [145] Although this treatment approach may avoid the adverse effects of early and high-dose fluid resuscitation, it carries the potential risk of tissue hypoperfusion. It is included in the damage-control resuscitation (DCR) protocol and provides optimal fluid resuscitation and transfusion to patients with hemorrhagic shock secondary to severe trauma. The main DCR principles are: (1) permissive hypotension/hypotensive resuscitation; (2) rapid and definitive/surgical control of bleeding; and (3) the prevention/treatment of hypothermia, acidosis, and hypocalcemia;

Its main endpoints are: (1) to minimize iatrogenic resuscitation injury; (2) to prevent worsening of initial traumatic shock; and (3) to obtain definitive hemostasis [169, 170].

For patients with major trauma, defined by an ISS ≥ 16, the American College of Surgeons’ Advanced Trauma Life Support (ATLS) guidelines currently advocate “balanced” resuscitation with an initial 1–2 L of crystalloids before definitive/surgical control of bleeding [27]. The European guideline on management of major bleeding and coagulapathy following trauma [145] recommends a restricted fluid resuscitation to achieve specific target blood pressure using serum lactate and/or base deficit measurements to estimate and monitor the extent of bleeding and shock.

Permissive hypotension and possible cut-offs in the traumatized elderly are highly debated, but there is no evidence to-date to definitive recommendations. A RCT demonstrated that permissive hypotension did not significantly reduce 90-day mortality compared with usual care in critically ill patients aged ≥ 65 years who received vasopressors for vasodilatory hypotension, [171].

In elderly patients, blood pressure is not sufficient to estimate tissue perfusion. Accordingly, permissive hypotension can be dangerous in this vulnerable category of patients [47, 50, 83–85]. Data suggest that 110 mmHg is the threshold value associated with increased mortality in geriatric blunt trauma patients [87]. Vasopressors which is administrated to increase blood pressure in the hypovolemic hemorrhagic untreated shocked patient may worsen tissue perfusion by causing vasoconstriction [75, 84–158, 172].

An association between early vasopressor use and poor outcome in trauma was reported [169, 170].

There are no definitive trials focused on elderly trauma patients that could answer if the administration of vasopressors versus permissive hypotension have benefits in the early resuscitation [156]. Evidence focused on geriatric trauma patients, according to mechanism of trauma, are lacking. Further studies are needed.

**Key Question 3.5**

**How intraoperative hypotension status is correlated with delirium in geriatric patients?**

**Statement 3.5.1**

There is evidence correlating the occurrence of postoperative delirium and perioperative hemodynamic changes (QoE C low).

**Recommendation 3.5**

We suggest assessing, as early as possible, the risk factors for the onset of delirium because it is related to unfavourable outcomes in trauma geriatric patients. **(Weak recommendation based on a moderate-low quality level of evidence 2B].**

### Summary of evidence and discussion

Delirium is an acute and often fluctuating disturbance in attention and awareness that is extremely common among hospitalized older adults, with an incidence of 29–64% in general medical wards, 50% after high-risk surgical procedures, and up to 75% in patients receiving mechanical ventilation in the intensive care unit [173, 174].

It is associated with adverse outcomes, including increased risk of falls, functional decline, dementia, prolonged hospitalization, institutionalization, and death, at an annual cost of $38 billion to $152 billion in the US [175, 176]

Risk factors associated with delirium are advanced age, dementia, cognitive impairment, frailty, history of delirium or other central nervous system disorders, cumulative comorbidities, alcohol use, depression, malnutrition, and functional, visual, or hearing impairment [177]. In a systematic review, a total of 112 precipitating factors were identified including surgical factors, tachypnea, hypotension, fever or hypothermia, type and depth of anesthesia, infection, fand trauma [177].

Neerland and al [178] investigated the associations between perioperative hemodynamic changes, use of vasopressor drugs, risk of acute delirium and risk of long-term dementia in patients presenting with hip fracture who had surgery. Risk factors for postoperative delirium were low body mass index, low level of functioning, severity of physical illness, and receipt of ≥ 2 blood transfusions. Long-term dementia was associated with severity of physical illness, delirium, receipt of vasopressor drugs perioperatively, and high mean arterial pressure postoperatively.

Radinovic and al [179] investigated the impact of intraoperative blood pressure on onset of postoperative delirium and showed that a higher MAP had a protective effect on the occurrence of postoperative delirium in patients managed for hip fracture. This issue has not been comprehensively addressed in the trauma setting. Predictive models developed in elective surgery settings do not directly report intraoperative hypotension among predictors of postoperative delirium. Some variables, including age, anesthesia methods, Mini-Mental State Examination (MMSE) score, hypoxia during operation, company of family members, serum concentration of IL-6 above 9 ng/ml, and major hemorrhage and other related to hypotension may be involved in the development of delirium [180–183].

Studies focused on the prevention, assessment and management of geriatric patients’ delirium are lacking in the trauma setting. Further studies are needed to prevent and manage delirium in this group of patients according to age and frailty.

## Management of oral anticoagulants

**Key Question 4.1**

**Which blood tests are useful to evaluate geriatric patients on anticoagulant drugs in trauma setting?**

**Statement 4.1.1**

When assessing the risk of bleeding in the emergency setting, the type of anticoagulant ingested, time of ingestion, age, renal and hepatic function must be evaluated (QoE A high).

**Statement 4.1.2**

Prothrombin Time and INR are a reliable methods to assess clinically relevant exposure to oral vitamin K antagonists anticoagulants (QoE A high).

**Statement 4.1.3**

Due to the low sensitivity and specificity of the Prothrombin Time (PT) and Activated Partial Thromboplastin Time (aPTT) to direct oral anticoagulants (DOACs), they are not reliable to assess DOACs activity, especially apixaban and endoxaban [QoE A high].

**Statement 4.1.4**

A normal Thromboplastin Time (TT) suggests that there is no clinical relevant Dabigatran activity (QoE B moderate).

**Statement 4.1.5**

Although viscoelastic testing including TEG or ROTEM has been advocated to guide the identification of trauma coagulopathy, it’s role in elderly patients on anticoagulant therapy is not established (QoE B-C moderate-low).

**Statement 4.1.6**

More reliable and faster qualitative and quantitative tests of coagulation such as calibrated drug-specific anti-Xa levels should be strongly considered in the workup of geriatric trauma patients (QoE B-C moderate-low).

**Statement 4.1.7**

Anti-Xa assay is the gold standard for monitoring Low Molecular Weight Heparin (LMWH) therapy (QoE A high).

**Statement 4.1.8**

Quantitative assays, such as ecarin clotting time (ECT), dilute thrombin time (dTT), and anti-Xa inhibitor, which can accuracy determine DOACs plasma concentrations, are not readily available in all hospitals. They can be utilized, if the results don’t require urgency, as second-line tests (QoE B-C moderate-low).

**Recommendation 4.1**

We recommend performing routinely the common coagulation assays in elderly patients including the Activated Partial Thromboplastin Time (aPTT), Thromboplastin Time (TT), Prothrombin Time (PT), INR, and anti-Xa levels to assess early anticoagulants exposure in the trauma setting. There is not enough evidence to support the routinely use of TEG or ROTEM in elderly trauma patients. Further studies are necessary to determine their role. **[Strong recommendation based on a moderate level quality of evidence 1B].**

### Summary of evidence and discussion

Elderly trauma patients using anticoagulants drugs have been steadily increasing [184, 185]. Acute trauma coagulopathy and bleeding occur in 25–35% in trauma patients, 3% of whom are on anticoagulant therapy having an increased risk of bleeding [186–188]. VKAs, by inhibiting the enzyme Vitamin K oxide reductase, reduce levels of factors II, VII, IX and X and the natural anticoagulants Proteins C and S. These drugs, due to its narrow therapeutic index, their potential of life-threatening effects, and their numerous drugs and food interactions, requires frequent monitoring [189]. Monitoring VKAs is accomplished by measuring the prothrombin time (PT). This assay responds to a reduction of 3 (FII, FVII, and FX) of the 4 vitamin K-dependent clotting factors. Although it is simple to perform, PT is limited by the variability of the different thromboplastins reagents in their respone to the anticoagulant effect of VKA [190].The International normal ratio (INR) was established to standardise the PT assay. It is the ratio between the patient’s PT and the PT of normal plasma raised to potential of the international sensitivity index (ISI) of the thromboplastin [191]. PT-INR measurements are an excellent assay for trauma patients using VKAs.

Heparin is a glycosaminoglycan containing a pentasaccharide that binds to and enhances the activity of antithrombin III. This binding reduces thrombin generation via inihibiting coagulation factors (XIIa, IXa, XIa, and Xa) [192]. Heparins were classified into Unfractionated Heparin (UFH) and Low molecular-weight heparin (LMWH). The most widely laboratory test which measures the anticoagulant in clinical practice is the Activated Partial Thromboplastin Time (aPTT) [193]. Despite this, there are several factors that can make aPTT monitoring troublesome and questionable. Its sensitivity depends on other variables like standardizing the reagents used, lack of correct synthesis of coagulation factors, consumption of coagulation factors in active bleeding or thrombosis, and liver or hematological disorders increasing baseline aPTT [194, 195]. UFH may also be monitored through anti-factor Xa (anti-Xa) activity. Anti-Xa is more efficient in achieving the therapeutic range of UFH compared with aPTT. Nevertheless, this does impact clinical outcome [196, 197]. Compared with aPTT, it is more costly, has little clinical expertise in its interpretation, and is less available. Anti-Xa assay is the gold standard for monitoring LMWH therapy [198].

Direct oral anticoagulants (DOACs) specifically inhibit thrombin or activated factor X (FXa) which affects commonly used global coagulation assays as well as select special coagulation tests [199–201]. Their safety profile, low risk of spontaneous bleeding and the predictable bioavailability lacking the need of regular monitoring tests, made them the preferred choice in anticoagulation therapy. Dabigatran is a direct thrombin inhibitor that competitively inhibits thrombin and thus prevents the conversion of fibrinogen to fibrin. Peak plasma concentration occurs 2–3 h after ingestion and it is eliminated predominantly by the kidneys. Therefore, impairment in renal function can extend the drug half-life and predispose to higher bleeding risks compared to other DOACs [202].

Rivaroxaban is a direct, reversible inhibitor of activated factor X (FXa). It prevents the conversion of prothrombin to thrombin, which thereby prevents fibrin formation [203]. Peak plasma concentration occurs after 2 h, its elimination is by the kidney, where the unmetabolized drug is excreted in the urine, and by the liver. Both kidney and hepatic impairment increase plasma rivaroxaban levels [204].

Apixaban and Endoxaban, are direct, reversible inhibitors of FXa, with a high affinity for FXa which inhibits free FXa, FXa in the prothrombinase complex, and FXa bound to platelets. Apixaban has a peak plasma concentration at 3 to 4 h, and it is eliminated by the intestine and kidney. Endoxaban has a faster peak plasma concentration of 1–2 h [191, 205].

Hence, it is necessary to interpret the population age, gender, renal function, bioavailability and pharmacodynamics-kinetics to understand the usefulness and interpretation of the coagulation assays.

Common coagulation assays (CCA) such as PT and aPTT are readily available in all hospitals and have been used as first-line tests to supply a qualitative assessment. Dabigatran may prolong clotting assays such as aPTT. The concentration–response curve for prolongation of the aPTT is non-linear at its higher concentrations (≥ 200 ng/mL) [206–209]. Additionally, the results of this assay vary between reagents and their different sensitivities to dabigatran [207]. PT/INR are mildly elevated on dabigatran, but these tests are less sensitive than aPTT. For example, aPTT levels can be twofold that of normal levels at the peak concentration of dabigatran. However, after 12 h, aPTT levels can still be as high as 1.5 times that of normal levels and therefore it is difficult to correlate the timing of dabigatran use with aPTT levels [188]. A normal aPPT has limited value in the trauma bay, especially in out of the peak plasma drug concentration.

On the other hand, rivaroxaban prolongs PT in a dose-dependent manner, but this quantitative effect varies based on different thromboplastins because of their differing sensitivities to rivaroxaban [208]. As international normalized ratio (INR) and the international sensitivity index (ISI) are based on VKA sensitivity. PT should not be interpreted as INR in patients treated with DOACs. PT can be a helpful tool determining the presence of DOACs in the trauma setting [209]. Rivaroxaban also prolongs the aPTT, but this assay is less sensitive than the PT [209–212].

Despite lack of publications on apixaban and endoxaban, the qualitative CCA demonstrated that most PT and aPTT reagents show only mild or modest sensitivity for the drug [213, 214]. Depending on the reagent used, PT may remain normal at therapeutic concentrations of apixaban [215]. CCAs are not recommended for estimating the relative anticoagulation intensity or plasma concentration of apixaban and endoxaban after therapeutic doses. In general, direct thrombin inhibitors (Dabigatran) tend to affect the aPTT more than the PT. In contrast, direct FXa inhibitors (Rivaroxaban, Apixaban and Endoxaban) impacts the PT more than the aPTT, nevertheless, a normal aPTT and/or PT cannot rule out the DOAC effect [216].

An analysis of a prospective observational study across 16 trauma centers [217] showed that admission INR values were mildly elevated among patients on dabigatran and rivaroxaban compared with apixaban, patients on dabigatran presented with slightly higher than normal aPTT values whereas those on rivaroxaban and apixaban did not.

Ali et al. [209], in a Trauma Level 1 Center, evaluated a cohort of 54 patients to determine the impact of pre-injury anticoagulation on CCA and TEG variables. CCA, identified a greater proportion of coagulopathy for patients on pre-injury anticoagulation therapy. This result demonstrated prolongation of the INR or aPTT, in 67% of the patients in anticoagulation therapy. aPTT values for patients exceeded the threshold for coagulopathy in 100% of patients on dabigatran, PT-INR in 85% of VKAs patients, and 63% of patients on anti-Xa inhibitors.

Although quantitative assays, such as ecarin clotting time (ECT), dilute thrombin time (dTT), and anti-Xa inhibitor have accuracy in determining DOACs plasma concentrations, they are not readily available in all hospitals. They can be utilized, if the results don’t require urgency, as second-line tests. These assays are complex, costly, not widely available and have a long turn-around time. In trauma patients its utility is clinically limited. Thrombin time (TT) is another useful tool for bleeding patients using dabigatran. This test is very sensitive and important. Its normal assay excludes the presence of relevant dabigatran levels. Nevertheless, the assay is too sensitive for quantification of above therapy levels [218, 219]. To address this, issue dilute TT was developed which appears to have a good linear correlation with the drug plasma level [202].

Another range of tests, such as ecarin clotting time (ECT), ecarin chromogenic assay (ECA) and anti-FXa activity can be usefull but they are limited to high complexity laboratories. Furthermore, their longer turn-around time inhibits their utilization in the emergency department [220–223].

Viscoelastic coagulation test, including the thromboelastograph (TEG) and rotational thromboelastogram (ROTEM), allows rapid detection of coagulation disorders. This point of care assay reproduces information on clot development, stabilization, and dissolution in short time. The assay is immediately accessible in trauma and perioperative settings [224]. They demonstrated that R times and clot formation times (CFT) are correlated with dabigatran and rivaroxaban concentration. However, they perform better with peak samples, losing their sensitivity to detect residual drug activity in patients. In an experimental porcine model study, Grotke et al. [225] investigated the anticoagulant effects of dabigatran in combination with trauma-induced bleeding using ROTEM parameters. The authors observed prolongation of Clot Time (CT) and reduced clot strength which was increased by bleeding after trauma.

Van Ryn et al. [226] showed that viscoelastic coagulation tests may be useful in the detection of coagulopathy associated with dabigatran and in monitoring the effects of reversal therapy. These tests are fast, feasible in trauma setting, and can allow prompt decision making. Dias et al. [227] after characterization in vitro effects of DOACs, concluded that TEG can be used to guide reversal therapies. Bliden et al. [228] evaluated patients on dabigatran, rivaroxaban and epixaban, and found that automated TEG6s has more than 92% sensitivity and 95% specificity indicating that TEG can be an effective tool to identify the anticoagulant effects of DOAC facilitating the care of bleeding patients. Seyve et al. [229] compared plasma DOACs concentrations on 3 commonly ROTEM tests and demonstrated the DOAC dose-dependent increase in ROTEM CTs. CTs were poorly impacted by low levels of edoxaban, rivaroxaban or dabigatran. Apixaban had a low effect even at high concentrations. Drug concentration must be taken into consideration when interpreting the assay. Kobayashi et al. [216] sought to determine if DOACs were associated with abnormal values on CCA or TEG. They did not show significant differences in TEG ability to detect clinically significant coagulopathy at admission nor significant impact of reversal therapy. The median values for R, alpha, and maximum amplitude (MA) were within normal limits and did not differ significantly between DOACs. PT values correlated moderately with the R value on TEG only among patients on rivaroxaban. aPTT correlated with the R value on TEG strongly in patients on dabigatran and moderately in patients on rivaroxaban but did not correlate for patients on apixaban. Ali et al. [209] found that TEG identified coagulopathy only in 50% of patients on dabigatran and 8% on VKAs and suggested that TEG should not be used to guide anticoagulation reversal decisions in acute trauma patients.

A urine dipstick is a point of care method that can screen dabigatran from anti-Xa DOACs. It may be useful in trauma settings due to rapid assessment. Its limitations include difficulties in reading pads due to urine color, the lack of correlation with plasma DOAC concentrations, and delay between drug ingestion and urine detection [230].

**Key Question 4.2**

**Which reversal protocol is indicated in patients being treated with vitamin K antagonists?**

**Statement 4.2.1**

Oral vitamin K antagonists reversal protocol is indicated in elderly patients presenting with hemorrhagic shock not responding to supportive measures or needing for an urgent surgery or invasive procedure (QoE A high).

**Statement 4.2.2**

Anticoagulated patients with oral vitamin K antagonists (VKAs) presenting with head trauma but without radiographic evidence of Intracranial hemorrage (ICH) or other bleedings should not receive preventive reversal (QoE B-C moderate-low).

**Statement 4.2.3**

If an emergency surgical exploration is indicated and it can be delayed for 6–12 h, in trauma elderly patient with a history of VKAs treatment, the INR can be corrected by administrating intravenous vitamin K, in selected cases (QoE A-B high-moderate).

**Statement 4.2.4**

For surgery that requires reversal of oral vitamin K antagonists, and which cannot be delayed for vitamin K to have time to take effect, the INR can be corrected by giving prothrombin complex concentrate (PCC) and intravenous vitamin K. PCC should not be used to enable elective or non-urgent surgery (QoE C low).

**Statement 4.2.5**

Oral vitamin K antagonists (VKAs) reversal agents should be managed according to INR level (QoE B moderate).

**Statement 4.2.6**

PCC, preferably intravenous (4F-PCCs), is strongly recommended for prompt oral vitamin K antagonistsVKAs reversal. If those are not available Fresh Frozen Plasma (FFP), III factor Prothrombin Complex (3PCC), or recombinant FVIIa could be used (QoE B moderate).

**Statement 4.2.7**

Vitamin K administration alone is not recommended as a reversal agent in patients with life-threatening bleeding, but as an adjunct treatment in these patients (QoE A-B high-moderate).

**Statement 4.2.8**

The use of Recombinant activated factor VII (rFVIIa) as VKAs reversal agent increases the risk of thromboembolic events, especially in elderly patients (QoE B moderate).

**Recommandations 4.2**

We recommend administrating a reversal agent in elderly trauma patients anticoagulated with oral vitamin K antagonists who present with bleeding, not responding to supportive measures, major life-threatening bleeding, bleeding located in critical organs (central nervous system, abdominal, thoracic), or needing urgent surgical or invasive procedures **[Strong recommendation based on a moderate level quality of evidence 1B].**

We recommend using the reversal protocol including intravenous factor prothrombin complex concentrates (4F-PCCs) and 5 mg intravenous vitamin K in case of life-threatening bleeding and/or urgent surgical procedures. Further doses should be administered if needed to achieve INR < 1.5 **[Strong recommendation based on a high level quality of evidence 1A].**

We recommend giving Fresh frozen plasma (FFP) as oral vitamin K antagonists (VKA) agent reversal only if no other treatment is available **[Strong recommendation based on a moderate quality level of evidence 1B].**

We do not recommend the use of recombinant activated coagulation factor VII (rFVIIa) as first-line VKA reversal agent** [Strong recommendation based on a low level of quality evidence 1C].**

### Summary of evidence and discussion

Underlying cardiac, respiratory, or renal dysfunctions associated with previous drugs administration, can significantly alter the fluid management and product resuscitation goals in a bleeding elderly trauma patient.

Warfarin is the most widely prescribed oral anticoagulant for the prevention and treatment of thromboembolism. The reported annual incidence of any bleeding is 25.8%, major bleeding ranges from 1.3 to 7.2%, while intracranial haemorrhage (ICH) is up to 2.5% [231]. In the largest study, after adjusting for covariates, warfarin use was associated with an odds ratio (95% confidence interval) of 1.32 (1.05–1.65) for trauma-related mortality [232, 233].

Collins et al. evaluated a cohort of Medicare patients with head trauma and found that pre-injury warfarin increased the odds of intracranial hemorrhage by 40%, and doubled the risk of mortality [234]. These data highlight the importance of obtaining accurate medication histories, so anticoagulated patients could be identified, and reversal strategies considered. Based on the patient’s history and results from the primary survey and physical examination, radiographic studies should be performed to identify occult injuries that could also be complicated by the patient’s anticoagulation status. Generally, the risk of bleeding increases simultaneously with an increase in the international normalized ratio (INR). Therefore, appropriate laboratory testing should be done to establish the degree of coagulopathy on presentation. In an emergency setting, the strategy for oral anticoagulant reversal depends on the type of drug; the presence, location, and level of bleeding; and the need for surgery.

The current treatment options for the reversal of warfarin anticoagulation effect includes withholding warfarin, administering vitamin K1 (phytomenadione), fresh frozen plasma (FFP), and prothrombin complex concentrate (PCC) [145]. FFP is prepared from whole blood and plasma apheresis donations and contains both pro-coagulant and anticoagulant proteins. It is indicated for coagulation factor replacement in patients with multiple coagulation factor deficiencies including vitamin K–dependent coagulation factor deficiency due to warfarin therapy. Administration of FFP requires ABO blood group compatibility testing, thawing, and infusion. The adverse effects of FFP include allergic reactions, acute lung injury, transmitted diseases, circulatory overload and thromboembolic events [145]. Prothrombin complex concentrate (PCC) is a concentrated vitamin K–dependent coagulation factor product (factors II, VII, IX, and X) derived from large donor pooled plasma that is stored as a lyophilized powder. PCCs are available as 3-factor [3F-PCCs] (factors II, IX, X) or 4-factor[4F-PCCs] (factors II, VII, IX, X) concentrates. Three-factor PCCs have small amounts of natural anticoagulants (protein C and S), but 4F-PCCs have concentrated amounts of protein C and S and small amounts of heparin.

Guidelines [235–238] suggest that patients with major or life-threatening VKA-associated bleeding that all anticoagulants must be discontinued, and reversal agents must be administered if available. Major bleeding as defined by the Control of Anticoagulation Subcommittee refers to either bleeding with hemodynamic compromise and/or bleeding in a critical anatomic site (intracranial, pericardial, intraspinal, intraocular, retroperitoneal, intra-articular, or intramuscular with compartment syndrome) and/or an acute drop in hemoglobin by more than 2 g/dL or the requirement of more than 2 units of blood, or massive transfusion [239]. This should not delay fluid and blood resuscitation and local measures to control the bleeding whilst ensuring normothermia, normal acid base status and ionized calcium. Ivascu et al. demonstrated more than 75% decrease in mortality related to posttraumatic intracranial hemorrhage in elderly patients with Coumadin-related coagulopathy after implementation of a protocol to ensure rapid head computed tomography, initiation of INR-correcting therapy within 1.9 h, and full correction of coagulopathy within 4 h of admission. The same authors suggested that reversal of INR is not necessary in the absence of intracranial bleeding [240, 241].

While reversal is important in the situations described above, the risk of subsequent thromboembolic events due to reversal, ranging from 7.2–12% within 30 days from the event, should also be kept in mind [242, 243]. Thus the decision on when and how to restart anticoagulation following an episode of acute bleed is also important.

Although large-volume FFP was previously the standard of care for VKA reversal in this population, PCC has become the treatment of choice for VKA reversal in emergency setting. American [236], British [238] and European [237], French [244] clinical practice guidelines recommended PCCs over FFP for warfarin associated major bleeding or urgent procedure [245]. PCC offers several advantages over FFP such as fast reconstitution into a relatively smaller volume which can be infused over a shorter period (20–30 min), fast onset of action, no requirement for ABO compatibility, minimal risk of viral transmission due to pathogen reduction and inactivation processes conducted during manufacturing, and reduced risk of other clinical adverse reactions such as transfusion associated circulatory overload or transfusion-related acute lung injury [239, 246, 247]. Not needing cross-matching, and the speed of correction make PCCs the ideal agents for correction of warfarin anticoagulation in trauma patients. The risk involved in the use of PCCs is mainly allergic reactions, heparin- induced thrombocytopenia, and thromboembolic complications.

4F-PCC has been approved by the FDA for warfarin reversal since 2013 and has demonstrated efficient and effective reductions in INR with low thromboembolic events. However, the evidence on the efficacy and safety of reversal procedures is still based on clinical experience more than on sound evidence of net clinical benefit. RCTs are available for a subset of anticoagulated patients. Three randomized controlled trials comparing PCCs vs FFP were published in patients with life-threatening bleeding during VKA treatment [246, 248]. Overall, patients receiving 4-F PCCs achieved a more rapid INR normalization in case of ICH and urgent surgical interventions [247, 249].

A multicenter European prospective trial with 4-factor PCC showed an INR decline to 1.4 or less in 100% of patients at 30 min post-transfusion [250]. PCC use is also associated with a reduction in requirement of pack red blood cell (6.6 vs. 10 units; *P* = 0.001) and decline in mortality (23 vs. 28%; *P* = 0.04) [251, 252].

Sarode et al. [248] showed a lower incidence of fluid overload or cardiac events in the 4-factor PCC group compared with plasma group (4.9% vs 12.8%). A Cochrane Review evaluated 4-factor PCC compared to administration of FFP in patients with VKA associated bleeding or indication for emergent procedures. The authors conclude that PCC can reverse VKA associated INR prolongation without further requiring FFP or other blood products [253].

Yanamadala et al. reported a study of patients undergoing emergency reversal of VKA anticoagulation using either plasma (n = 28) or PCC (n = 5). The time to reversal was significantly shorter in the PCC group (65 vs 256 min; *P* < 0.05) and, consequently, surgery was performed sooner in the PCC group [254].

Similarly, a retrospective study about ICH in geriatric trauma patients, showed that PCC resulted in significantly faster INR reversal versus plasma. The incidence of ICH progression was decreased with PCC compared to plasma (17.2% vs 44.2%; *P* = 0.031) [255].

Rapid reversal of coagulopathy in geriatric patients on warfarin is vital to limit the extent of ICH. Adopting such a protocol is associated not only with a more rapid reversal and less FFP use, but also will prevent further intracranial hematoma expansion and facilitate rapid surgical evacuation [256, 257].

Quick et al. [256] reported their preliminary experience with PCC for warfarin reversal of geriatric patients in rural trauma setting. Fifteen patients who were taking warfarin before injury, received PCC (15 to 30 IU/kg) alone or in conjunction with FFP. Compared with 10 patients who received FFP alone, they showed benefit in patients receiving PCC including lower volume infusion (< 50mL vs 1 L), a trend towards fewer units of FFP administration, and a greater decrease in INR. Although significance was not reached, fewer units of PRBC transfusion were required in the PCC group. This indicates a potential positive impact of PCC use in rural hospitals with limited financial resources and limited ready access to blood bank facilities.

Four-factor PCC is preferred over 3-factor PCC in view of the more successful INR reversal, with less adverse events [258].

Four-factor PCC is administered intravenously in a dose of 25–50 U/kg. There are algorithms available with which to calculate the most appropriate dose based on bodyweight and INR level. At present, the FDA approves a variable dosing protocol for administration of 4FPCC for warfarin-induced anticoagulation based on patient weight and presenting INR. A stepwise dosage is recommended, e.g. 25 U/kg if INR is 2–4.0, 35 U/kg if INR is 4–6.0 and 50 U/kg if INR is > 6.0 [257].

Safaoui et al. [259] published a preliminary report using 3-factor PCC for 28 patients on warfarin that presented with traumatic brain injury. They received 2,000 units of PCC pre-emptively following the protocol. Mean INR was significantly reduced (5.1 to 1.9, *P* = 0.008). The mean time to correction of INR was 13.5 min. Joseph et al. [260] reviewed 45 coagulopathic trauma patients who received 3-factor PCC (Profilnine SD). INR was significantly reduced with PCC (2.6 ± 1.0 vs. 1.5 ± 0.2, *P* = 0.001) in the 25 patients with pre-injury warfarin use. The Australian Society of Thrombosis and Hemostasis (ASTH) 2013 guideline recommends using 3F-PCC with or without FFP. 3F-PCC reverses the INR level faster with or without a low dose of FFP or vitamin K1 [261].

However, studies on 3F-PCC are limited due to the preference for four-factor (4F-PCC) use in most countries. Current literature on the efficacy of 3F-PCC is inconsistent. In a prospective, multicenter, observational trial detailing 256 patients who received PCC, no thromboembolic adverse events were documented after 4F-PCC administration. In contrast higher incidence of thromboembolic events has been reported in trauma patients with the use of three-factor PCC compared with four-factor PCC [262]. Therefore, in patients who have received PCC, thromboprophylaxis is prudent as early as possible after bleeding has been controlled. Cost-effectiveness is another positive impact related to the use of PCC. In a cost-effective analyses using the cost per life-year gained, Guest et al. [263] concluded that the use of PCC appeared to be more cost effective than FFP in VKA reversal after several types of hemorrhage. Reduction of potentially severe transfusion reactions and/or circulatory overload may counterbalance the higher cost of PCCs compared to FFP; in addition, FFP requires additional staff time to be prepared and administered. Treatment with FFP and vitamin K is recommended only if no other treatments are available. FFP should be administered at 10–20 mL/kg IV in combination with one dose of vitamin K 10 mg IV. Vitamin K alone is not recommended as a reversal agent in emergency setting because it could take from 4 to 24 h to normalize coagulation [264].

However, it is recommended as an adjunct treatment in these patients at the dosage of 5–10 mg administered intravenously. In cases of bleeding, vitamin K administration helps replete stores of clotting factors II, IV, IX, and X, thereby increasing the speed of reversal and reducing the INR over 4 h to 6 h for IV infusion and up to 24 h for oral administration. When emergent reversal is required, IV vitamin K should be given with PCC or FFP to prevent rebound because FFP’s half-life is 4 h to 6 h whereas the half-life of PCC is dependent on the clotting factors present, ranging from 4 h for factor VII to 60 h for factor II [265].

A rare and unpredictable but important side effect of intravenous vitamin K is an anaphylactic reaction, in some cases resulting in cardiac arrest, with an incidence of 3 per 100,000 doses via a non-immunoglobulin E (IgE) mechanism, possibly due to the solubiliser in the vitamin K solution [266]. “Overcorrection” of warfarin reversal with additional PCC and vitamin K1 can lead to harm. More than 10 mg vitamin K1 can prevent re-warfarinisation for days and over use of PCC (administration of further PCC when INR is in the normal range) may create a prothrombotic state, which could lead to further thrombosis [265].

Recombinant activated factor VII (rFVIIa) is a hemostatic agent that increases thrombin generation by activating factor X at the site of vascular injury. It should not be used as a single agent to reversal because it is usually not capable of restoring hemostasis. Limited evidence exists regarding the use of rFVIIa for reversal of VKA-related hemorrhage. The actual recommendation is not to use rFVIIa for warfarin reversal unless no other option is available, or in case of failure with previous treatments. Data from a literature review show that the use of rFVIIa as a prothrombotic agent could result in an increased risk of thromboembolic events, especially in elderly patients and when used for off-label indications such as the reversal of anticoagulant agents [267].

**Key Question 4.3**

**Which reversal protocol is indicated in patients in treatment with direct oral anticoagulants (DOACs)?**

**Statement 4.3.1**

In deciding if it is necessary to proceed with active reversal of direct oral anticoagulants (DOACs) in trauma setting, it is crucial to assess DOAC plasma concentration, in fact the administration of a reversal agent is useful only when the anticoagulant drug is active in patient's plasma in measurable quantities but only few centres have the DOAC mesurement available (QoE A-B high-moderate).

**Statement 4.3.2**

In general clinical practice, it is recommended to consider anticoagulant DOAC reversal for patients with serious bleeding and a DOAC level > 50 ng/mL, and for patients requiring an invasive procedure with high bleeding risk and a DOAC level > 30 ng/mL (QoE A high).

**Statement 4.3.3**

The main DOACs reversal agents are the idarucizumab (Praxbind*®*) for reversal of dabigatran and the andexanet alfa for reversal of apixaban and rivaroxaban (QoE A high).

**Statement 4.3.4**

Andexanet alfa is not indicated for DOACs reversal in patients requiring urgent surgery (QoE C low).

**Statement 4.3.5**

If a DOAC-treated patients requires an invasive procedure, the active reversal is indicated only if the procedure cannot be safely performed while the patient is anticoagulated, cannot be delayed, and there is demonstration or reasonable expectation that the patient has clinically relevant plasma DOAC levels (QoE B-C moderate-low).

## Recommendation 4.3

We recommend an early assessment of laboratory coagulation tests and direct measurements of DOAC levels, if quantitative tests are available, in elderly trauma patients receiving or suspected of having received a DOAC before deciding for reversal due to the thromboembolic risk **[Strong recommendation based on a moderate level quality of evidence 1B].**

We suggest the administration of DOACS reversal agents only in critically ill patients with dosable plasma DOAC levels and presenting with hemorrhagic shock not responding to resuscitation, when level of DOACS can be assessed **[Weak recommendation based on a moderate-low quality of evidence 2B].**

If the trauma patient with uncontrolled life-threatening bleeding, was treated with dabigatran (anti-FIIa activity), the suggested reversal protocol is to administer idarucizumab 5 g IV. If idarucizumab is not available, 50 units/kg IV of activated prothrombin complex concentrates (APCC) may be administrated **[Weak recommendation based on a moderate-low quality level of evidence 2B].**

In patients with rivaroxaban-associated or apixaban-associated (FX inhibitors) life-threatening and uncontrolled bleeding, the suggested reversal protocol is the administration of andexanet alfa as an intravenous bolus of 400 mg over 15 min followed by a continuous infusion of 480 mg over 2 h (low dose) or 800 mg over 30 min followed by a continuous infusion of 960 mg over 2 h (high dose), according to the last dose of DOAC and the size of the dose. If andexanet alfa is not available, 2000 units of four-factor prothrombin complex concentrates (PCC) may be administrated **[Weak recommendation based on a moderate-low quality level of evidence 2B].**

### Summary of evidence and discussion

The direct oral anticoagulants (DOACs), including dabigatran, which is direct thrombin inhibitors, and apixaban, betrixaban, edoxaban, and rivaroxaban, which are direct factor Xa inhibitors, have been approved by the United States Food and Drug Administration (US FDA) for prevention of stroke and systemic embolism in non-valvular atrial fibrillation (AF), prevention and treatment of venous thromboembolism (VTE), and secondary prevention of arterial ischemic events in patients with chronic coronary or peripheral artery disease [268–270].

AF affects an estimated 2.7–6.1 million people in the USA, and the incidence is expected to rise as the population ages and life expectancies increase [271]. The prevalence of AF is higher in the elderly with about 9% of people aged > 65 years and 2% of those aged < 65 years [272].

The risk for major bleeding with DOACs in patients with AF versus VKAs was reported ranging 1.6–3.6 for DOACs versus 3.1–3.6 for VKAs [273] The risk of major bleeding with oral anticoagulants in patients with VTE is 2% to 3% per year [274].

Major bleeds are defined those that result in death, are life-threatening, cause chronic sequelae or consume major health-care resources. The Control of Anticoagulation Subcommittee [275] defines major bleeding in non-surgical patients as: (1) fatal bleeding; (2) Symptomatic bleeding in a critical area or organ, such as intracranial, intraspinal, intraocular, retroperitoneal, intra-articular, pericardial, or intramuscular with compartment syndrome; (3) bleeding causing a fall in hemoglobin level of 20 g L)1 (1.24 mmol L)1) or more, or leading to transfusion of 2 or more units of whole blood or red cell.

In a 2015 meta-analysis [276] including 13 randomized trials, DOACs were associated with a 47% reduction in the risk of fatal bleeding (RR 0.53, 95% CI 0.43–0.64) compared with VKAs. The case-fatality rate was reported lower with DOACs than warfarin (10% vs 15%; RR 0.66, 95% CI 0.49–0.89) [277]. Although the risk of major bleeding with DOACs compared with VKAs is decreased, patients taking DOACs may present with serious bleeding because of trauma or need for an urgent unplanned procedure.

Two specific DOACs reversal agents have been approved by the US FDA: the idarucizumab for reversal of dabigatran and the andexanet alfa for reversal of apixaban and rivaroxaban [278].

Idarucizumab (Praxbind*®*)is a humanized monoclonal antibody fragment that binds irreversibly to free and thrombin-bound dabigatran within few minutes. It is administered as two consecutive rapid bolus doses of 2.5 g intravenous, no more than 15 min apart [279].

The RE-VERSE AD (Reversal Effects of Idarucizumab on Active Dabigatran) study [280] was carried out to assess whether 5 g of intravenous idarucizumab could reverse the anticoagulant effect of dabigatran. Five-hundred three patients with uncontrolled bleeding (301 patients in the group A) or needing for urgent procedure (202 patients in the group B) were enrolled. The median maximum percentage reversal of dabigatran was 100% (95% CI, 100 to 100), on the basis of either the diluted thrombin time or the ecarin clotting time. In group A, 137 patients (45.5%) presented with gastrointestinal bleeding and 98 (32.6%) presented with intracranial hemorrhage; among the patients who could be assessed, the median time to the cessation of bleeding was 2.5 h. In group B, the median time to the initiation of the intended procedure was 1.6 h; peri-procedural hemostasis was assessed as normal in 93.4% of the patients, mildly abnormal in 5.1%, and moderately abnormal in 1.5%. At 90 days, thrombotic events had occurred in 6.3% of the patients in group A and in 7.4% in group B, and the mortality rate was 18.8% and 18.9%, respectively.

Andexanet alpha (Andexxa*®*) is a modified human recombinant factor Xa decoy protein that lacks catalytical activity. It is a specific urgent reversal agent for rivaroxaban and apixaban that binds with high affinity to direct Xa inhibitors and also indirect Factor Xa inhibitors, such as low-molecular-weight heparin and fondaparinux [279].

The dosing of andexanet alfa depends on the DOAC and on the timing since last intake: for rivaroxaban (with the last intake > 7 h before reversal) or apixaban, a 400 mg bolus is administered followed by a 480 mg infusion (4 mg/min). For rivaroxaban (with the last intake < 7 h before reversal or unknown recent intake), edoxaban or enoxaparin, an 800 mg bolus followed by a 960 mg infusion (8 mg/min) is given [https://www.portola.com/wp-content/uploads/Andexxa-prescribing-information-pdf.pdf.].

The **ANNEXA-4** (Prospective, Open-Label Study of Andexanet Alfa in Patients Receiving a Factor Xa Inhibitor Who Have Acute Major Bleeding) [281] study was carried out to evaluate the andexanet alfa reversal efficacy in patients presenting with major bleeding ≤ 18 h after taking an FXa inhibitors (apixaban, rivaroxaban, edoxaban). Three-hundred fifty-two patients were enrolled and treated with andexanet alfa administered as an intravenous bolus, followed by a 2-h infusion, according to the type of FXa inhibitor and timing of the last FXa inhibitor dose. At the end of the andexanet bolus, anti-FXa activity was reduced from baseline by 92% (95% CI 91–93) in apixaban-treated patients and by 92% (95% CI 88–94) in rivaroxaban-treated patients. This effect persisted for the duration of the infusion, followed by a subsequent increase in anti-FXa activity seen 4 h after discontinuation. Of 254 patients, 82% had good/excellent hemostasis at 12 h (95% CI 77–87), with similar results for gastrointestinal (85%; 95% CI 76–94) and intracranial (80%; 95% CI 74–86) bleeding. Andexanet has not been studied in patients requiring urgent surgery and is not approved for this indication.

In patients suffering from an intracerebral haemorrhage, andexanet alfa reduced anti-FXa activity with a high rate of haemostatic efficacy and a beneficial outcome [282].

PCC contain plasma-derived inactive vitamin K–dependent coagulation factors, with 3-factor (factors II, IX, and X) and 4-factor (factors II, VII, IX, and X) formulations available. Four-factor PCC may antagonize the anticoagulant effect of FXa and thrombin inhibitors. PCC increases prothrombin and factor X levels inducing a compensatory pro-hemostatic effect with increased thrombin generation potential. The effect of PCCs on laboratory indices of DOAC anticoagulant effect has been studied in in vivo/ex vivo experiments, animal models, and human volunteers, showing conflicting and, at best, modest effects [283]

In a cohort study [284], 4-factor PCC (weight < 65 kg, 1500 units; weight > 65 kg, 2000 units) was administered to 84 prospectively enrolled patients with major bleeding who were receiving apixaban or rivaroxaban. The median time from FXa inhibitor administration to PCC treatment was 12 h. Hemostasis was classified as effective in 69% of patients. In another cohort study, 4-factor PCC (fixed dose of 2000 units) was administered to 66 prospectively enrolled patients with apixaban- or rivaroxaban-related major bleeding [285]. The median time from last dose of FXa inhibitor to PCC administration was 17 h (IQR, 12–21). Hemostasis was judged as good in 65%, moderate in 20%, and poor/none in 15% of patients. Propensity score-matched analysis showed that the adjusted 30-day mortality rates were lower for patients treated with andexanet alfa than in matched patients receiving PCC [286]. A retrospective single center compared the safety and effectiveness of andexanet alfa versus four-factor PCC reversal in ICH. It showed no significant differences in good or excellent ICH hemostasis within 24-h, or new thrombotic events within 14-days between the two drugs [287].

In deciding if it is necessary to proceed with active reversal of DOACs in a trauma patient, it is important to dispose of DOAC plasma concentration. The administration of a reversal agent can be useful only when the anticoagulant drug is active in patient's plasma in measurable quantities [288–291].

In 2015 the International Society on Thrombosis and Haemostasis recommends to consider anticoagulant reversal for patients with serious bleeding and a DOAC level > 50 ng/mL, and for patients requiring an invasive procedure with high bleeding risk and a DOAC level > 30 ng/mL. In 2019 this recommendation was revised because of the lack of specific DOACS tests in several hospitals [289]. Because of their dependence on renal function for clearance, all DOACs have higher blood levels and longer half lives in patients with renal dysfunction. Direct FXa inhibitors are partially metabolized by the liver. Patients with renal dysfunction may also present with uremia-associated platelet dysfunction, and may benefit from administration of desmopressin acetate or cryoprecipitate, and optimization of renal status with hemodialysis [288, 292–294]. Dabigatran is the only DOAC that can be removed by hemodialysis [289].

Early assessment of both laboratory coagulation tests and direct measurements of DOAC levels is crucial in trauma patients receiving or suspected of having received a DOAC. Quantitative assays for dabigatran include the dilute thrombin time, ecarin clotting time, and ecarin chromogenic assay. The preferred test for quantitation of apixaban, edoxaban, and rivaroxaban is a chromogenic anti-FXa assay calibrated with the drug of interest [290]. It they are not available or they can delay the management of the bleeding patient, qualitative assays can be performed.

Dabigatran (direct thrombin inhibitor) tends to influence aPTT more than PT. A prolonged aPTT suggests the presence of on-therapy or above on-therapy levels of dabigatran; a normal aPTT does not exclude the presence of on-therapy levels. A normal TT excludes clinically relevant dabigatran levels, but a prolonged TT does not discriminate between clinically important and insignificant drug concentrations [289, 290].

In contrast, direct FXa inhibitors (Rivaroxaban, Apixaban and Endoxaban) impacts the PT more than the aPTT, nevertheless, a normal aPTT and/or PT cannot rule out the DOAC effect [289, 290]

An (universal LMWH-calibrated) anti-Xa activity assay may determine rivaroxaban, apixaban and edoxaban concentrations and correctly predicts relevant drug concentrations [289, 290].

Viscoelastic coagulation tests may be helpful, in particular in patients with alterated liver function, since most DOACs prolong the clotting time (ROTEM or ClotPro) progressively [232, 295].

When managing an elderly trauma patient treated with DOAC, if anti-FXa activity has been detected, andexanet alfa should be administrated as reversal agent; if it is not available or patients are on edoxaban, PCC (25–50 U/kg) treatment may be initiated. In case of anti-FIIa activity due to dabigatran, idarucizumab (5 g i.v.) should be initiated. The co-administration of tranexamic acid (15 mg/kg or 1 g) is indicated in trauma patients independent of the present DOAC and reversal strategy [296, 297].

## Antibiotics, analgesia and anti-thrombotic prophylaxis

**Key Question 5.1**

**When is it indicated to administer antibiotics in elderly trauma patients?**

**Statement 5.1.1**

Antibiotic prophylaxis including a single pre-operative narrowest spectrum antibiotic dose covering aerobic and anaerobic bacteria, is commonly administered after penetrating abdominal trauma in all patients before surgical exploration (QoE C low).

**Statement 5.1.2**

If an exploratory laparoscopy/laparotomy is required, in case of peritoneal cavity contamination due to a perforated hollow viscus, antibiotics are administered out of prophylaxis and in high risk patients including immunocompromised patients or patients with American Society of Anesthesiologists (ASA) score > 3, or obesity (QoE B-C moderate-low).

**Statement 5.1.3**

If present, the level of peritoneal contamination and the presence of signs of sepsis and shock are the main factors to guide the administration and duration of an antimicrobial treatment in trauma patients (QoE C low).

**Statement 5.1.4**

There is no evidence confirming the benefit of a long course of antibiotic prophylaxis (> 24 h) compared to a short course (≤ 24 h) on abdominal surgical site infection, mortality, or intra-abdominal infection, in the absence of risk factors (obesity, immunosuppression, high ASA score) for post-operative septic complications (QoE B moderate).

**Statement 5.1.5**

The antibiotic of choice should be active against the common bacteria causing surgical site infections in peritonitis, such as *Escherichia coli* or other *Enterobacteriales* or *Clostridiales* (QoE B moderate).

**Statement 5.1.6**

Antibiotic prophylaxis in patients with thoracostomy and penetrating thoracic trauma can prevent infectious complications and protect against empyema and pneumonia (QoE A high).

**Statement 5.1.7**

In blunt chest trauma, antibiotics showed no protective effect against empyema or pneumonia.

(QoE C low).

**Statement 5.1.8**

The use of antibiotic prophylaxis in blunt chest trauma when inserting a chest drain is required, and mostly in chest penetrating trauma to reduce the risk of empyema and pneumonia. (QoE A high).

**Statement 5.1.9**

The use of antibiotics for preventing infection in open limb fractures is recommended (QoE A high).

**Statement 5.1.10**

In soft tissue penetrating injuries, broad spectrum, empirical, intravenous antibiotic therapy should be commenced once deep samples have been obtained, and then tailored once organisms and sensitivities are known. A short course, single agent regimens using cephalosporins in order to prevent adverse outcomes in soft tissue injuries associated with bony injury (open fractures) is recommended. Hand fractures do not require routine prophylaxis. (QoE B moderate).

**Statement 5.1.11**

A specific antibiotic or preferred dosing when employing local antibiotics in the management of fracture related infections cannot be recommended. (QoE C low).

**Statement 5.1.12**

The use of antibiotic prophylaxis as a protective factor for SSI after open reduction and internal fixation of ankle and closed extremity fractures is recommended (QoE A high).

**Statement 5.1.13**

In severely burned patients, the role of an adequate source control including the surgical removal of contaminated material and areas of necrosis and protection of the exposed lesion is crucial in decreasing infectious complications. Antibiotic prophylaxis could protect against septic complications in the high risk patients (QoE C low).

**Recommendations 5.1**

We recommend antibiotic prophylaxis in penetrating (abdominal, thoracic) trauma, in severely burned and in open fractures in elderly patients to decrease septic complications **[Strong recommendation based on a high-moderate quality level of evidence 1A].**

We recommend early empiric antibiotic therapy in patients presenting with signs of sepsis and septic shock and high risk patients (obesity, immunocompromised, high ASA score) in penetrating abdominal trauma, which should be active against common bacteria causing surgical site infections in peritonitis, such as *Escherichia coli* or other *Enterobacteriales* or *Clostridiales* **[Strong recommendation based on a moderate quality level of evidence 1B].**

We recommend against the administration of antibiotics in blunt trauma in absence of signs of sepsis and septic shock **[Strong recommendation based on a moderate-low quality level of evidence 1B].**

### Summary of evidence and discussion

Current guidelines supporting the use of prophylactic antibiotics for penetrating abdominal trauma are mostly based on expert opinion and on trials with several bias. There is currently no information from randomized controlled trials to support or refute the use of antibiotics for patients with penetrating abdominal trauma, without peritoneal contamination and peritonitis. No specific considerations can be deduced from literature for elderly patients. Similarly, to extend the duration of antibiotic prophylaxis for people undergoing laparotomy for penetrating abdominal trauma beyond 24 h and the choice of certain drug regimens cannot be recommended based on strong evidence.

### Abdominal penetrating and blunt trauma

In clinical practice, antibiotic prophylaxis is commonly administered, including a single pre-operative broad spectrum antibiotic dose, covering aerobic and anaerobic bacteria, and continuation (up to 24) in case of an exploratory laparotomy/laparoscopy, according to expert opinion recommandations. If a hollow viscus perforation is found, the antibiotics are continued [295, 298, 299].

A Cochrane systematic review [300] was carried out with the aim to assess the benefits and harms of prophylactic antibiotics administered for penetrating abdominal injuries to reduce the incidence of septic complications including septicemia, intra-abdominal abscesses and wound infections. No randomized controlled trials were found focusing on this issue.

In 2019, a systematic review was carried out to assess the effects of antibiotics in penetrating abdominal trauma, focusing on the type of agent administered and the duration of therapy [301]. Twenty nine RCTs, with a total of 4458 participants, showing a very low quality of evidence. There was no evidence confirming the benefit of performing a long course of antibiotic prophylaxis (> 24 h) compared to a short course (≤ 24 h) on abdominal surgical site infection, mortality, or intra-abdominal infection. Fifteen studies, involving 2020 participants, were found comparing different drug regimens with activity against three classes of gastrointestinal flora (gram positive, gram negative, anaerobic), but no strong evidence was found on the benefit of one regimen over another [301].

Emergency laparotomy is correlated with high incidence of SSI. The infection rate after trauma laparotomy ranges between 7.1 and 28.4% for superficial and deep SSI, and 7.9–25.2% for IAI [302, 303].

The implementation of a protocol for antimicrobial prophylaxis with the use of ertapenem for trauma laparotomy showed that SSI could be reduced in a single centre experience [304].

Antimicrobial prophylaxis is strongly suggested in clean contaminated and contaminated surgical procedure associated with a high incidence of SSIs, in high risk patients including immunocompromised patients or patients with American Society of Anesthesiologists (ASA) score > 3 or obesity [305]. The antibiotic of choice should be active against the common bacteria causing SSIs in peritonitis, such as *Escherichia coli* or other *Enterobacteriales* or *Clostridiales* [305].

It is important to choose antibiotics with the narrowest spectrum of activity to avoid the selection of resistant bacteria [305]. The antibiotic should be administrated in the right dose according to weight and organ function at the right time to increase its concentration and effectiveness [305].

If present, the level of peritoneal contamination and the presence of signs of sepsis and shock are the main factors to guide the administration and duration of an antimicrobial treatment in trauma patients. The antibiotic regimen has to be adapted rapidly to the microbiological results and revaluated according to biological and clinical features [305].

There are no reported benefits in long term antibiotic treatment versus short duration in critically ill patients [306]**.**

### Thoracic blunt and penetrating trauma

Retained hemothorax is a risk factor for developing pneumonia and empyema [307] alongside with pathological contact with the outside environment in penetrating trauma. Seventy percent to 90% of severe thoracic trauma patients may need tube thoracostomy [308]. Tube thoracostomy treats both pneumothorax and hemothorax, evacuating the content of the thoracic cavity and reducing the incidence of subsequent empyema. Nevertheless, the post-traumatic empyema rate varies from 2 to 25%, with S. aureus being responsible for 35–75% of subsequent infections [308]. Presumptive antibiotic use in thoracostomy has a clear role in preventing infectious complications in chest trauma patients. When stratified by trauma type, antibiotic prophylaxis showed to be protective in penetrating injuries, against empyema and pneumonia. In blunt trauma, antibiotics showed no protective effect against empyema or pneumonia [309, 310].

Other studies [311, 312], showed that in patients with blunt or penetrating thoracic trauma requiring the insertion of a chest drain, the administration of an antibiotic prophylaxis was associated with a reduced risk for post-traumatic empyema and pneumonia.

Further studies are required to define the optimal type, dose, and duration of antibiotic administration in thoracic trauma patients and in the elderly.

### Traumatic limb fractures

Fractures are common in geriatric population and SSI is a significant post-operative complication after open reduction and internal fixation of traumatic fractures. A metanalysis was carried to evaluate the risk factors of SSI after open reduction and internal fixation of ankle fracture. It showed that BMI, ASA ≥ 3, diabetes, alcohol, open fracture, subluxation/dislocation, incision cleanness grade 2–4, high-energy injury mechanism, chronic heart disease, history of allergy, and use of antibiotic prophylaxis were identified as risk factors for the development of SSI after open reduction and internal fixation of ankle fracture [313].

A randomized double-blinded placebo-controlled trial showed that there is no statistically significant difference between patients who received prophylactic postoperative cefazolin for 23 h versus placebo although it decreased the risk of SSI after open reduction and internal fixation of closed extremity fractures. Patients with diabetes mellitus are more likely to develop SSI [314].

There is evidence supporting the use of antibiotic prophylaxis in the management of open fractures [315]. Short course, single agent regimens using cephalosporins in order to prevent adverse outcomes in open fractures is the recommended approach. However, there is no conclusive evidence supporting prophylactic antimicrobial use in the management of small soft tissue upper extremity trauma and simple lacerations. The updated Surgical Infection Society guidelines recommend against administration of extended-spectrum antibiotic coverage compared with gram-positive coverage alone to decrease infections complications, hospital length of stay or mortality in type I or II open extremity fractures. In type III open extremity fractures, it is recommend to administer an antibiotic therapy for no more than 24 h after injury, in the absence of clinical signs of active infection, to decrease infectious complications, hospital length of stay or mortality. SIS recommends against extended antimicrobial coverage beyond gram-positive organisms to decrease infectious complications, hospital length of stay or mortality. In type III open extremity fractures with associated bone loss, it is recommended to administer antibiotic therapy in addition to systemic therapy to decrease infectious complications [316].

There is promising literature on the beneficial effects of the use of local antibiotics, e.g. by antibiotic beads in managing traumatic fractures. Coating of internal fixation devices is a modern approach to improve infection prophylaxis and gentamicin-coated implants have been demonstrated to be safe in clinical application [317, 318].

### Burn patients

Infections among burn patients are common and are associated with high mortality rate. In a series of 175 patients with severe burns, infections preceded multiorgan dysfunction in 83% of patients and were considered as the direct cause of death in 36% of patients [319].

Systemic antibiotic prophylaxis administered in burn patients in the first 4–14 days significantly reduced all cause mortality by nearly a half; limited perioperative prophylaxis reduced wound infections but not mortality. Topical antibiotic prophylaxis applied to burn wounds had no beneficial effects [320].

Barajas-Nava [321] reviewed 36 RCTs (2117 participants); twenty-six (72%) evaluated topical antibiotics, seven evaluated systemic antibiotics (four of these administered the antibiotic perioperatively and three administered upon hospital admission or during routine treatment), two evaluated prophylaxis with non absorbable antibiotics, and one evaluated local antibiotics administered via the airway. There was a statistically significant increase in burn wound infection associated with silver sulfadiazine compared with dressings/skin substitute (OR = 1.87; 95% CI: 1.09 to 3.19, I(2) = 0%), with significantly longer length of hospital stay compared with dressings/skin substitute (MD = 2.11 days; 95% CI: 1.93 to 2.28). Systemic antibiotic prophylaxis was evaluated in three trials (119 participants) and there was no evidence of an effect on rates of burn wound infection. Systemic antibiotics (trimethoprim-sulfamethoxazole) were associated with a significant reduction in pneumonia (only one trial, 40 participants) (RR = 0.18; 95% CI: 0.05 to 0.72) but not sepsis (two trials 59 participants) (RR = 0.43; 95% CI: 0.12 to 1.61). Perioperative systemic antibiotic prophylaxis had no effect on any of the outcomes of this review. There was a statistically significant increase in rates of MRSA associated with use of non-absorbable antibiotics for selective decontamination of the digestive tract with non-absorbable antibiotics plus cefotaxime compared with placebo (RR = 2.22; 95% CI 1.21 to 4.07).

The role of an adequate source control including surgical removal of contaminated material and areas of necrosis and protection of the exposed lesion is crucial in decreasing the infective risk. Antibiotic prophylaxis could protect the high risk patients from infectious complications [305].

**Key Question 5.2**

**How to control pain in elderly patients admitted for trauma?**

**Statement 5.2.1**

Pain assessment is crucial in obtaining an effective pain control in elderly trauma patients (QoE B-C moderate-low).

**Statement 5.2.2**

Opioids administration should be avoided in elderly patients in the trauma setting to reduce side effects (QoE B-C moderate-low).

**Statement 5.2.3**

Multimodal analgesic approach or “balanced analgesia” including regional and peripheral nerve blocks and neuroaxial analgesia should be implemented in elderly patients pain control, in the trauma setting (QoE B moderate).

**Statement 5.2.4**

Regular intravenous administration of acetaminophen is effective and safe in elderly trauma patients (QoE B-C moderate-low).

**Statement 5.2.5**

Opioids administration for post-traumatic pain in elderly patient should consider a progressive dose reduction because of high risk of morphine accumulation and subsequent over-sedation, respiratory depression and delirium (QoE A high).

**Statement 5.2.6**

Non-pharmacological approaches play an important role in improving trauma pain, including immobilizing limbs and applying dressings or ice packs in conjunction with drug therapy (QoE C low).

**Recommendations 5.2**

We recommend a regular administration of intravenous acetaminophen every 6 h as first line treatment in managing acute trauma pain in the elderly in a multimodal analgesic approach** [Strong recommendation based on high quality level of evidence 1A].**

We suggest considering to add NSAIDs in elderly patients presenting with severe pain, taking into account potential adverse events and pharmacological interactions **[Weak recommendation based on a moderate quality level of evidence 2B]**.

We recommend the implementation of Multi-Modal-Analgesia approach (MMA) in trauma setting for elderly injured patients including acetaminophen, gabapentinoids, NSAIDs, lidocaine patches, and tramadol and opioids only for breakthrough pain for the shortest period of administration at the lowest effective dose **[Strong recommendation based on a moderate quality level of evidence 1B].**

We recommend peripheral nerve blocks placement in elderly patients with acute hip fractures at the time of presentation to reduce preoperative and postoperative opioid use for analgesia **[Strong recommendation based on a high quality level of evidence 1A].**

We suggest the adoption of epidural analgesia and regional anaesthesia to control severe pain in acute hip fractures in selected elderly patients **[Weak recommendation based on a moderate quality level of evidence 2B].**

In elderly patients with ribs fractures, we recommend the association of systemic analgesic treatment with thoracic epidural and paravertebral blocks to offer an adequate pain control with limited contraindications and improvement in respiratory function, reducing opioid consumption, infections and delirium, if skills are available **[Strong recommendation based on a high quality level of evidence 1A]**.

We recommend to routinely consider the use of epidural or spinal analgesia for management of postoperative pain in elderly patients who undergo major thoracic and abdominal procedures for trauma, if skills are available **[Strong recommendation based on a high-quality level of evidence 1A]**.

We recommend carefully evaluating the use of neuraxial and plexus blocks for patients receiving anticoagulants to avoid bleeding and complications **[Strong recommendation based on a high-quality level of evidence 1A]**.

We suggest the implementation of non-pharmacological measures such as immobilizing limbs and applying dressings or ice packs in conjunction with drug therapy*,* in control acute pain in elderly patients in the trauma setting **[Weak recommendation based on a very low level of evidence 2D].**

### Summary of evidence and discussion

The treatment of pain in the elderly can be challenging particularly in patients with underlying cognitive impairment, who are less able to communicate. Patients with cognitive impairment would receive less pain medication, have poorer mobility, poorer quality of life and higher mortality than patients with intact cognition. Under-treated pain and inadequate analgesia increase stress and are risk factors for agitation, aggression, wandering, delay in mobilization, development of chronic pain, refusal of care and delirium in elderly patients [177, 322, 323].

The key steps in managing pain in trauma patients are the assessment of pain and the regularly evaluation of pain relief [322, 323].

Several studies demonstrated that the assessment and delivery of pain-relieving medication is suboptimal for geriatric trauma patients.

It was reported that 42% of patients over the age of 70 years old didn’t receive adequate analgesia, even when patients reported moderate to high level of pain after closed-isolated extremity and clavicular fractures [324].

One in three older adults presenting to the ED with non-operative fragility pelvic fractures receive no analgesia during the course of their prehospital and ED care [325].

A study evaluating the impact of age on pain perception in the ED, found that older adults experience the same level of pain as their younger counterparts from dislocations and fractures [326].

Pain assessment based on the patient’s self-report is the most accurate and reliable evidence of the existence of pain and its intensity for patients of all ages, regardless of communication or cognitive deficits [327].

For that, a variety of tools [328–330] were developed to quantify pain intensity, such as the numeric rating scale (NRS), rating pain from 0 to 1; the verbal descriptor scale (VDS), including series of phrases which descried different levels of pain intensity (e.g., “no pain,” “mild pain,” “moderate pain,” “severe pain,” “extreme pain,” and “the most intense pain imaginable”) and the faces pain scale (FPS), providing series of progressively distressed facial expressions to be choosen by the patient according to the severity or intensity of his/her current pain; and the visual analogue scale (VAS), consisting of a 10-cm line, with the left-hand side labeled “no pain” and the right-hand side labeled “most intense pain imaginable” (or similar descriptor).

The appropriate pain measurement scale should be selected according to individual’s ability to read, hear, and understand how to complete the tool.

In non-communicative older adults with cognitive impairment and dementia, the pain assessment can rely on observational and surrogate (family, certified nursing assistants) reports. Six main types of pain behaviors and indicators were described [331] including:

- Facial expressions as slight frown, sad, frightened face, grimacing, wrinkled forehead, closed or tightened eyes, any distorted expression, rapid blinking;
- Verbalizations, vocalizations as sighing, moaning, groaning, grunting, chanting, calling out, noisy breathing, asking for help;
- Body movements such as rigid, tense body posture, guarding, fidgeting increased pacing, rocking, restricted movement, gait, or mobility changes;
- Changes in interpersonal interactions such as aggressive, combative, resisting care, decreased social interactions, socially inappropriate, disruptive, withdrawn, verbally abusive
- Changes in activity patterns or routines such as refusing food, appetite change, increase in rest periods or sleep, changes in rest pattern, sudden cessation of common routines, increased wandering.
- Mental status changes such as crying or tears, increased confusion, irritability, or distress.

For patients with severe dementia, the Pain Assessment IN Advanced Dementia (PAINAD), the Functional Pain Scale, or Doloplus-2 are better than other tests. [332, 333]. In non-verbal patients who cannot provide self-report, pain behaviors such as guarding and grimacing and input from family and caregivers associated with the Critical care Pain Observation Tool (CPOT) and Behavioral Pain Scale (BPS) are valid tools [334].

In the treatment of acute pain in injured patients, drugs should be administrated early; they should be quick and easy to administer; have short half-life, high effectiveness, and minimal side effects.

The choice of drugs and appropriate methods of administration should consider the response to and the need for continuous analgesia in the management of the patient.

In trauma patients, medication selection must focus on those with the least negative effects on hemodynamic status of the patient.

Common analgesics used are opioids, N_2_O, paracetamol or acetaminophen and non-steroidal anti-inflammatory drugs (NSAIDs). The type of analgesics used are tailored according to the type of injury, pain severity, triage system, and patient’s clinical features [335].

A dutch double-blind, randomized, clinical trial including 182 patients treated with acetaminophen, 183 with diclofenac, and 182 with combination treatment, showed that acetaminophen is not inferior to non-steroidal anti-inflammatory drugs, or the combination of both, in minor musculoskeletal trauma [336].

Regular intravenous administration of acetaminophen every 6 h, unless contraindicated, is effective in traumatic pain relief. NSAIDs need to be used with caution in elderly patients due to their potential adverse events, such as acute kidney injury and gastrointestinal complication. In the perioperative pain management of elderly patients with hip fractures, NSAIDs are usually not recommended [337].

However, if NSAIDs are administrated for pain relief in elderly trauma patients a proton pump inhibitor should also be co-prescribed and particular attention should be paid to patients who are on angiotensin-converting enzyme inhibitors, diuretics or antiplatelets because of drug interactions [338].

Opioids are the cornerstone in the management of trauma patients and should be considered in moderate to severe pain. They are effective in pain control but associated with serious cardiovascular events, acute dyspeptic syndrome with nausea and vomiting and an increased risk of respiratory failure [339].

Elderly trauma patients are particularly vulnerable to opioid use disorders and the high risk of morphine accumulation and subsequent over-sedation and respiratory depression.

Oxygenation with assisted ventilation was required in 0.05% of patients treated with ketamine, in 0.02% of patients treated with fentanyl and in 0% of patients treated with morphine. Nausea and vomiting were the main adverse effects of morphine (4.8%), fentanyl (1.5%) and ketamine (0.5%), while hypotension occurred in 1.6% of cases with fentanyl and 0.5% of cases with morphine [340].

Tramadol is a centrally acting analgesic with 2 mechanisms of action: it has a weak opioid agonist activity and inhibits serotonin re-uptake. It has a reduced depressive effect on the respiratory and gastrointestinal systems in comparison with other opioids; however, confusion may be a problem for older patients. Tramadol may reduce the seizure threshold and is contraindicated in patients with a history of seizures [341].

Opioid use concomitantly with other central nervous system depressants (e.g., benzodiazepines, skeletal muscle relaxants, gabapentinoids, etc.) has to be avoided outside of specific clinical scenarios in highly monitored settings. N-methyl-D-aspartate receptor antagonists (ketamine, magnesium), membrane stabilisers (lidocaine), anticonvulsants (gabapentinoids), antidepressants (amitriptyline) and ∝-agonists (clonidine, dexmedetomidine) are administrated alone or in combination with other analgesics to improve their effect. When selecting an adjuvant agent, physicians should prescribe medications with the lowest side effect profile for the geriatric patient, titrate the drug slowly, and assess patients carefully for both effectiveness and the presence of adverse effects, which have to be anticipated and managed accordingly. Laxative therapy, such as the combination of a stool softener and a stimulant laxative should be prescribed in a patient treated with opiods [341].

Several alternatives to opioids were proposed in literature. Recently methoxyflurane, an inhaled non-opioid analgesic with a rapid onset of pain relief was approved in low-dose for emergency relief of moderate-to-severe trauma-related pain in adults. A recent multicenter, randomized, controlled, open-label trial in adult patients (age range 19–91 years) showed that inhaled methoxyflurane (3 mL) is effective in providing superior short-term pain relief to intravenous morphine in patients with severe trauma pain [342].

A meta-analysis was carried out to compare the efficacy and safety of low-dose methoxyflurane with standard of care analgesics in adults with trauma-related pain. This meta-analysis showed that pain intensity reduction was statistically superior with low-dose methoxyflurane compared with standard of care analgesics (overall estimated treatment effect = 11.88, 95% CI 9.75–14.00; *P* < 0.0001). Significantly more patients treated with methoxyflurane achieved response criteria of pain intensity ≤ 30 mm on a visual analog scale, and relative reductions in pain intensity of ≥ 30% and ≥ 50%, compared with patients who received standard of care analgesics. The median time to pain relief was shorter with methoxyflurane than with standard of care analgesics. The findings were consistent in the subgroup of elderly patients (aged ≥ 65 years) [343].

The multimodal analgesic approach (MMA) or “balanced analgesia” was introduced with the aim of decreasing the exposure to opioids, to address acute pain effectively, and enhance recovery after surgical procedure and trauma. It is defined as the integrated use of multiple strategies including systemic analgesics, regional analgesic techniques, and non-pharmacological interventions to affect peripheral and/or central nervous system sites in the pain pathway with the main aim of achieving a synergistic effect of the various classes of drugs used at lower analgesic doses [344–349].

MMA provides the use of: (1) analgesics, including opioids, nonopioid analgesics (such as acetaminophen and NSAIDs), the gabapentinoids (gabapentin and pregabalin), serotonin norepinephrine reuptake inhibitors, tricyclic antidepressants, and *N*-methyl-d-aspartate (NMDA) receptor antagonists; (2) neuraxial (epidural and intrathecal) analgesia; (3) peripheral nerve blocks; and (4) intra-articular and wound infiltration with local anaesthetics.

The synergy created when multimodal regimens are used to target discrete components of the peripheral and central pain pathways leads to effective analgesia at lower opioid dosing, reducing related risk and producing fewer adverse effects [343, 344, 349] MMA should be individualized in a muldisciplinary approach according to the patient; type of pain; mechanism of pain (inflammatory or neuropathic); type of surgical procedure; location of pain; expected duration of pain. Because of their opioid-sparing effects, multimodal strategies are useful and safe for elderly patients [345, 346].

The MAST (Multi-modal Analgesic Strategies in Trauma) study [345] was a randomized, pragmatic, trial aimed to compare the original multimodal analgesic protocol regimen (MMPR) (intravenous administration, followed by oral, acetaminophen, 48 h of celecoxib and pregabalin followed by naproxen and gabapentin, scheduled tramadol, and as needed oxycodone) and the MAST MMPR (oral acetaminophen, naproxen, gabapentin, lidocaine patches, and as needed opioids). This strategy used a fixed schedule of acetaminophen, gabapentinoids, non-steroidal antiinflammatory drugs (NSAIDs), lidocaine patches, and tramadol with stronger opioids available only for breakthrough pain. It was reported that MMA reduce opioid exposure with a substantial reduction in patient-reported pain scores. This is due to administering non-opioid analgesics (e.g., paracetamol and NSAIDs) on a scheduled basis, rather than as needed, to mitigate the fluctuations between peak and trough serum levels.

A secondary analysis of the MAST data, showed that older trauma patients require fewer opioids than younger patients with similar characteristics and pain scores. Opioid dosing for post-traumatic pain should therefore consider age. A 20 to 25% dose reduction per decade after age 55 may reduce opioid exposure without altering pain control [346].

### Neuraxial and peripheral regional anesthesia

Regional analgesia is a crucial part of MMA. It includes peripheral nerve blocks (PNBs) with or without a continuous peripheral nerve block (CPNB) infusion directed toward an isolated nerve or plexus through the injection of a local anesthetic near the neural targets. These techniques allow a localized delivery of analgesia to specific painful areas and augment multimodal regimens [347].

Strong evidence confirm that the implementation of peripheral nerve blocks (PNBs) in managing acute pain associated with traumatic fractures in elderly patients is effective in decreasing the use of opioids, pain, and lenght of hospital stay [348].

In 2018, Steenberg et al. [350] made a systematic review of the literature (11 randomized and quasi-randomized controlled trials, with a total of 1062 patients) reported that the analgesic effect of fascia iliaca compartment block was superior to that of opioids during movement, resulting in lower preoperative analgesia consumption and a longer time for first request of analgesia, and reduced time to perform spinal anaesthesia. Block success rate was high and there were very few adverse effects. A Cochrane review (49 trials analyzed) was carried out to compare single shot PNBs used as perioperative, postoperative analgesia, or as a supplement to general anaesthesia versus no nerve block (or sham block) for adults with hip fracture. Three thousand sixty-one patients were enrolled; 1553 randomized to PNBs and 1508 to no nerve block with an average age of participants ranged from 59 to 89 years. PNBs reduced pain on movement within 30 min after block placement, risk of acute confusional state, chest infection, and time to first mobilization and probably costs [351].

A systematic review of 27 RCTs with 2478 cases assessed the use of fascia iliaca compartment block (FICB) as an analgesic strategy for perioperative pain management in geriatric patients with hip fractures after admission in the emergency department, and showed that this technique is safe, reliable, reproducible, and able to provide adequate pain relief compared with the conventional analgesia methods in the peri-ooperative management, promoting earlier mobilization, preventing complications, and reducing additional analgesic consumption [352].

Several studies investigated the efficacy of different type of PNB and showed a good pain control in patients presenting with hip fractures [353, 354].

The AnAnkle Trial, [355] a randomized blinded trial of two centers, which enrolled 150 patients reported that PNB anesthesia (ultrasound-guided popliteal sciatic and saphenous blocks with ropivacaine) decreased the postoperative pain intensity in primary ankle fracture surgery compared with spinal anesthesia and consequently the consumption of opioids, despite substantial rebound pain when PNBs subsided.

The prospective study carried out by Garlich et al. [356] in a level I trauma center focusing on 725 patients aged ≥ 65 yo reported that patients who received a preoperative fascia iliaca block (in single-shot, administering a 30- to 40-mL bolus of 0.25% bupivacaine with 1:200,000 epinephrine or in continuous, with a bolus of 10 to 20 mL of 0.2% bupivacaine, followed by a continuous infusion of 0.2% bupivacaine at 6 mL/h ending on the morning of postoperative Day 1) for hip fracture surgery consume less morphine preoperatively, with low rates of opioid-related adverse events.

Thompson et al. [357] prospectively randomized 44 patients into 2 groups to study the efficacy of a preoperative fascia iliaca compartment block in geriatric patients with fractures of the proximal femur. There was no significant difference in consumption of acetaminophen for mild pain, tramadol for moderate pain, or functional recovery between the 2 groups, but a statistically significant decrease in morphine consumption (0.4 mg vs. 19.4 mg, *P* = 0.05) and increase in patient-reported satisfaction (31%, *P* = 0.01). Using the same methodology in 97 patients randomized into 2 groups (experimental and no-block control), Schulte et al. [354] confirmed that single perioperative Fascia Iliaca Block (FIB) for patients with hip fracture surgery decrease opioid consumption and increase the likelihood to be discharged home. Similarly, the adoption of epidural analgesia (epidural infusion of bupivacaine/fentanyl or bupivacaine/morphine) and regional anesthesia are options for adequate pain relief in hip fractures [337].

Furthermore, PNBs administrated to patients with hip fractures and moderate cognitive impairment (a major risk factor for perioperative delirium) seem not to impact the cognitive status [358, 359].

Neuraxial anesthesia involves local administration of an anesthetic or opioid into the spinal cord's neuraxial (epidural or intrathecal) space. A local anesthetic and opioid combination work synergistically to relieve pain, but no single combination has proven superior to another. Decisions to use epidural analgesia either by single injection or by continuous infusion are often based on specific types and locations of pain, ability to closely monitor patients, and availability of anesthesia providers or pain service experts to oversee therapy [358, 359].

Risks associated with epidural analgesia include hypoventilation, atelectasis and pneumonia owing to the effects of local anesthetics on respiratory muscles and diaphragmatic excursion. Therefore, it has to be administered under closed monitoring in elderly injured patients, especially in those presenting with rib fractures [360].

The thoracic epidural (TE) and paravertebral blocks (PVB) have been considered as gold standard for analgesia for rib fractures since long time, offering an adequate pain control, even in coagulopathic and anticoagulated patients with some cautions. Hypotension might occur after TE. Vasopressors are often needed to offset this side effect. Motor block is a frequent occurrence and can limit mobilization. A systematic review supported the role of TE over other forms of analgesia (PVBs or parenteral opioids) for rib fractures pain control. TE and PVB were also shown to reduce opioid consumption and delirium in older people with rib fractures [361].

Recently, novel myofascial techniques such as erector spinae plane (ESPB) and serratus anterior plane (SAPB) blocks have been implemented in MMA for older patients. Teksen et al. conducted a randomised controlled trial and demonstrated that SAPB, as part of MMA in pain management for rib fractures, is safe and effective in reducing acute pain. The total tramadol consumption, the NRS scores and the chronic pain at rest and during effort were significantly less compared to the control group [362].

### Thoracic trauma

**Fig. 3:**
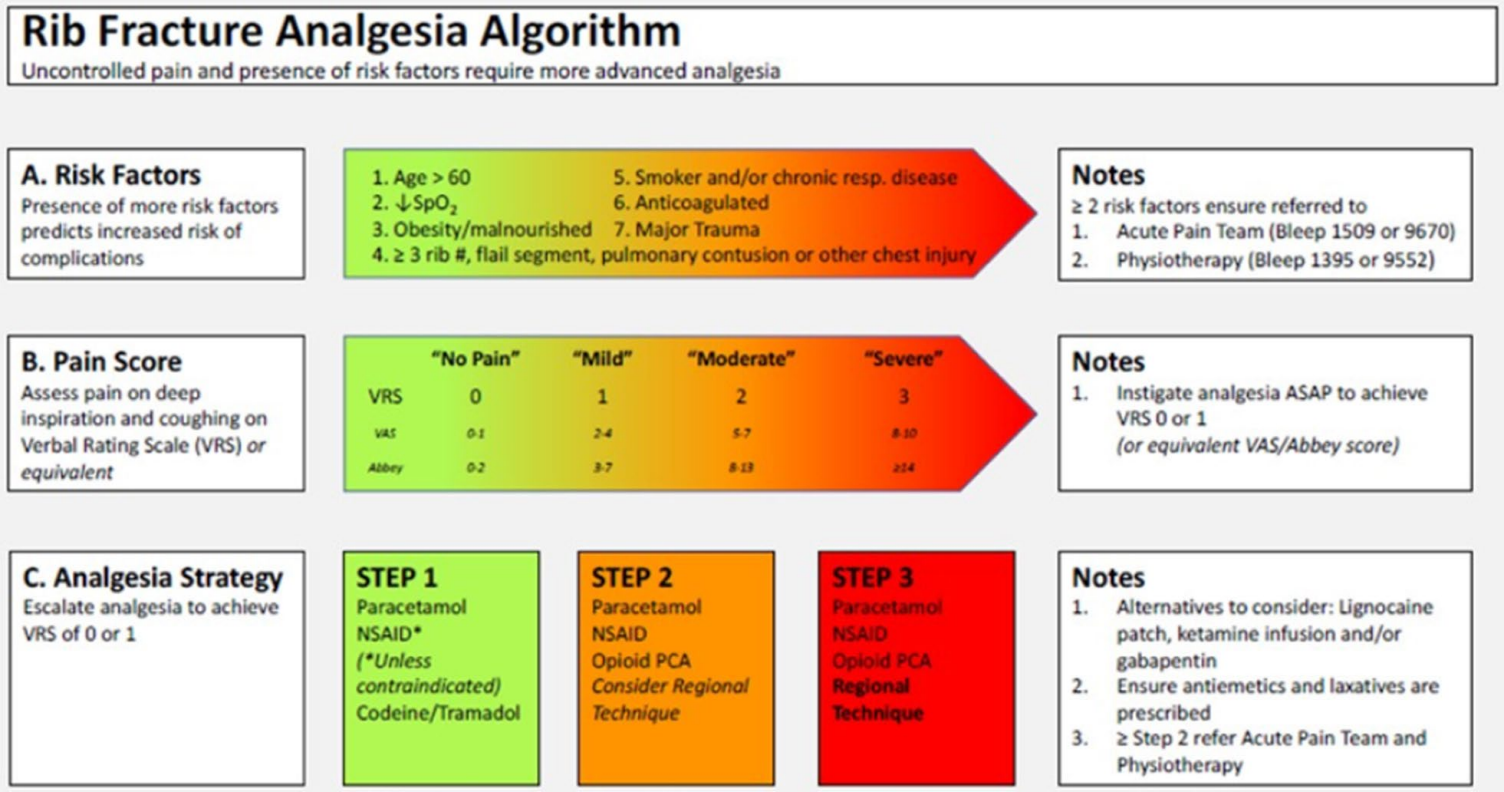
Rib fracture analgesia algorithm (Ref. [360])

Rib fractures occur in up to 40% of trauma patients. Overall mortality of patients having rib fractures is high, about 10% for all ages. Thoracic trauma is a common cause of trauma admission in elderly people. Mortality and morbidity from rib fractures primarily derive from pain-induced hypoventilation, pneumonia and respiratory failure. Acute pain management is importance to provide sufficient analgesia to allow respiratory rehabilitation and to prevent pulmonary complications [359–362] as showed in Fig. 3 [360].

Currently, opiate-based pain regimens are the cornerstone of rib fractures management, but they have several side effects in geriatric patients. Research tries to find safer alternative analgesics. Ketamine is an opioid alternative. It is an N-methyl-D-aspartate receptor antagonist and has been used widely for procedural sedations and intubations. High doses of ketamine may activate other receptors and result in undesirable adverse effects such as short-term hallucinations, night-time dreams, psychosis, dizziness, and blurry vision. To reduce these symptoms, it is proper to use low doses of ketamine, which also have analgesic effects and can decrease the overconsumption of opioids [363, 364].

A systematic review and meta-analysis showed that ketamine was non-inferior to morphine, and in low doses can be an alternative to opioids without side effects [364, 365] Recent systematic reviews and meta-analyses revealed that opioids had more side effects than ketamine and that ketamine did not cause life-threatening events [366, 367].

However, higher rates of neurological and psychiatric adverse events were seen in patients treated with ketamine whereas cardiovascular events were higher among patients using opioids [365]. Low dose ketamine administration reduced the use of morphine among patients with an ISS > 15, in the first 24 h after admission [368, 369].

Sub-dissociative intravenous-dose ketamine administered at 0.3 mg/kg over 15 min, provides analgesic efficacy comparable to morphine for short-term treatment of acute pain in the geriatric ED patients but results in higher rates of psycho-perceptual adverse effects [370].

Evidence supports the use of intravenous and oral administration of acetaminophen in a MMA approach when it is possible as primary treatment in elderly trauma patients with limited rib fractures presenting mild symptoms. Furthermore, oral acetaminophen is equivalent to intravenous acetaminophen for pain control (NO difference in morbidity or mortality) in elderly trauma patients with one or more rib fractures (138 patients; age ≥ 65 years), [371].

Several studies reported surgical rib fixation such as an important component of pain control.

Several indications have been proposed for considering surgical rib fracture repair including flail chest, severe pain and chest wall deformity [372].

A Cochrane meta-analysis evaluated the effectiveness and safety of surgical fixation in patients with flail chest, compared to conservative management [373]. They analysed data extracted from 3 studies with a total of 123 subjects and reported no statistically significant difference in mortality between the non operative and operative groups. However, in the surgical group, pneumonia, chest deformity and tracheostomy were reduced. Notably, the mean age of patients undergoing surgery was under 60 years old.

Sawyer et al. carried out a systematic review and meta-analysis to investigate the benefit of surgical rib fixation to identify the most “appropriate” patients for the operative treatment and reported a benefit of surgical treatment in terms of length of ICU stay, mechanical ventilation, mortality, pneumonia, and tracheostomy. The subgroup analysis showed that surgical fixation was most favourable for patients with flail chest and those who underwent surgical fixation within 72 h. However, patients over 60 years old showed to benefit from conservative management in terms of length of hospital stay and mechanical ventilation [374].

Focusing on elderly patients, Zhang et al. retrospectively analyzed data from 226 patients aged ≥ 60yo admitted for simple rib fractures managed operatively by internal fixation surgery and non-operatively. They showed that the pain score and fracture healing time were significantly improved in the operation group (*P* < 0.05) as well the duration of painkiller use was significantly shorter (*P* = 0.009). However, there was no significant differences in mortality, the incidence of bone nonunion, length of stay in the ICU, or duration of mechanical ventilation between the 2 groups [375].

Hoepelman et al. reviewed the literature to compare conservative and surgical ribs fixation in elderly patients (older than 60 years) with multiple rib fractures. The primary outcome was mortality. Secondary outcomes included hospital and intensive care length of stay, duration of mechanical ventilation and pneumonia rates. Five observational studies, including 2583 patients, were included. Mortality was lower in operatively treated patients compared to conservative treatment (4% vs. 8%). Pneumonia rate and duration of mechanical ventilation were similar (5/6% and 5.8/6.5 days) for either treatment modality. Overall hospital and intensive care length of stay were longer in operatively treated patients [376].

To the best of available evidence, it is unclear the individual contribution of operative and conservative treatment in reducing morbidity and mortality in the elderly with multiple rib fractures, and surgical ribs fixation can’t be recommended for pain control but an option to consider case by case.

### Abdominal trauma

Pain control after trauma laparotomy is important in limiting postoperative complications and mortality [373]. Inadequate analgesia may lead to distress and an increased risk of postoperative pulmonary and cardiac complications, thromboembolic events, and a greater stress response. Pain after laparotomy comes from the somatic afferent nerve fibres from the skin and muscle incision and the visceral pain from stretch and inflammation of the peritoneum. The visceral pain recovers more rapidly than the somatic pain [377].

The use of opioids to reduce somatic pain has to be reduced due to their side effects. A retrospective study on trauma laparotomy patients assessed the use of morphine for postoperative pain relief. Pain scores and time to first bowel movement and nasogastric tube duration were linked to the amount of opioids used. Frequent adverse effects of opioids included nausea, constipation, and preventing smooth postoperative recovery especially in elderly patients [378].

Provision of continuous thoracic epidural analgesia for at least 24 h after abdominal trauma surgery may improve survival, bowel recovery, and pulmonary function when compared to intravenous opioids. For midline laparotomies, thoracic epidural anesthesia and bilateral paravertebral bloc are comparably effective and provide superior analgesia compared to systemic opioids. Transversus abdominis plane (TAP) block, and Quadratus lumborum (QL) Block may be specifically adopted in this situation [379, 380].

Intravenous lidocaine given initially as a bolus (1–2 mg/ kg) then as an infusion (0.5–3 mg/kg/hr) can reduce opioid requirements and improve postoperative gastrointestinal motility [381]. A Cochrane review reported that it is uncertain whether intravenous perioperative lidocaine, when compared to placebo or no treatment, has a beneficial impact on pain scores in the early postoperative phase, and on gastrointestinal recovery, postoperative nausea, and opioid consumption. There is a lack of evidence about the effects of intravenous lidocaine compared with epidural anesthesia in terms of the optimal dose, timing, and duration [382]. Ketamine is efficacious as an analgesic in abdominal surgery, reduces opioid requirement, and can reduce the risk of the development of postoperative chronic pain. However, it is associated with the risk of postoperative psychiatric adverse effects [383, 384].

Magnesium reduces the requirement for opioids, improves analgesia, and can reduce the hyperalgesia seen with Remifentanil. Potential adverse effects of magnesium include hypotension and the prolongation of neuromuscular block [385]. Use of neuraxial and plexus blocks for patients receiving anticoagulants must be carefully considered and scheduled [386].

Key Question 5.3

**When and how is indicated to administer thrombo-prophylaxis in elderly trauma patients?**

**Statement 5.3.1**

The use of scoring systems to stratify the risk of venous thromboembolism (VTE) of elderly trauma patients is recommended (QoE C low).

**Statement 5.3.2**

Venous thromboembolism (VTE) pharmacological prophylaxis can be avoided in low risk elderly trauma patients (QoE C low).

**Statement 5.3.3**

Venous thromboembolism (VTE) pharmacological prophylaxis is recommended in moderate-high risk elderly trauma patients, if not controindicated (QoE C low).

**Statement 5.3.4**

Mechanical prophylaxis is recommended when pharmacological venous thromboembolism (VTE) prophylaxis is contraindicated (QoE C low).

**Statement 5.3.5**

Venous thromboembolism (VTE) pharmacological prophylaxis should be initiated as soon as possible in moderate-high risk patients and should be delayed of 24 h in case of Central nervous system injuries, active bleeding, coagulopathy, hemodynamic instability or solid organ injury (QoE C low).

**Statement 5.3.6**

Venous thromboembolism (VTE) pharmacological prophylaxis should be held in traumatic brain injury until computed tomography scan shows no progression (QoE C low).

**Statement 5.3.7**

Venous thromboembolism (VTE) pharmacological prophylaxis does not increase the rate of spinal hematoma in spinal injury (QoE C low).

**Statement 5.3.8**

Low Molecular Weight Heparin (LMWH) is recommended over un-fractionated heparin (UFH) to prevent deep vein thrombosis (DVT) (QoE C low).

**Statement 5.3.9**

The recommended dose of LMWH is 30 mg every 12 h. Dose adjustment according to anti-Xa levels and weight is warranted. In case of renal failure 5000 U of UFH every 8 h is recommended in elderly trauma patients (QoE C low).

**Statement 5.3.10**

Direct oral anticoagulants (DOACs) or aspirin may be considered as an alternative to heparin in view of better patient’s compliance after clinical stabilisation (QoE C low).

**Recommendations 5.3**

We recommend administering venous thromboembolism prophylaxis with LMWH or UFH as soon as possible in high and moderate risk elderly patients in the trauma setting according to the renal function, weight of the patient and bleeding risk **[Strong recommendation based on a low quality level of evidence 1C].**

If pharmacological prophylaxis of venous thromboembolism is contraindicated, we recommend mechanical prophylaxis **[Strong recommendation based on a low quality level of evidence 1C]**.

### Summary of evidence and discussion

The overall quality of evidence on the topic of venous thromboembolism (VTE) prophylaxis in elderly trauma patients is low as only observational retrospective studies and no RCTs are available. In addition, the vast majority of articles are on orthopaedic or neurologic trauma and very few articles include torso trauma. In order to achieve some recommendations, part of the indications were deduced from studies conducted on the general adult trauma population.

Trauma patients are at high risk of VTE mainly due to the reduction of mobility and to the inflammatory state generated by the trauma itself. For this reason VTE prophylaxis is usually recommended in these patients [387, 388]. The old age (> 60 years) has frequently been demonstrated to be an additional risk factor for VTE in trauma patients [389–405], as well as clearly mentioned also in the EAST guidelines for the management of VTE in trauma patients [399]. As a matter of fact, stratified age is among the major predictors of risk of VTE used to calculate both the Greenfield Risk Assessment Profile (RAP) [400]** (**Table 3**)** and the Trauma Embolic Scoring System (TESS) [401]** (**Table 4**)** which used in the recent Western Trauma Association guidelines algorithm for the management of VTE in trauma patients [402]. The 10 years retrospective study by Kim et al. [393] included 2500 elderly trauma patients (> 65 years old). The rate of VTE was 3.2%. Traumatic brain injury (*P* < 0.05); chest Abbreviated Injury Score > 3 (*P* < 0.001); mechanical ventilation (*P* < 0.001); major surgery (*P* < 0.001); and history of VTE (*P* < 0.05) were found to be independent predictors of VTE. Similarly, the 2 years retrospective study by Prabhkaran et al. based on a national trauma improvement program [392], selected 354,000 patients older than 65 years with post-traumatic VTE and demonstrated that being a male, ICU length of stay (LOS), overall LOS, spine injury, lower extremities injury, age > 75, severe traumatic brain injury, ventilator days, plasma transfusions within 24 h of admission were independent risk factors for deep vein thrombosis (DVT).

**Table 3:** Greenfield score < 5: low risk, >  = 5: high risk

Predictor		Greenfield Points
Age (years)	40–59	2
	60–74	3
	≥ 75	4
Obesity		2
Malignancy		2
Coagulopathy		2
History of TE		3
Femoral CVC > 24 h		2
Blood transfusions (> 4U in 24 h)		2
Surgery > 2 h		2
Vascular surgery		3
AIS chest/abdomen/head > 2		2 each
GCS < 8 for > 4 h		3
Complex lower extremities fracture		3
Pelvic fracture		4
Spinal cord injury with para- or quadriplegia		4

**Table 4:** TESS score 0–2: low risk. 3–6: moderate. 7–14: high risk

Predictor		TESS Points
Age (years)	18–29	0
	30–64	1
	≥ 65	2
ISS	1–9	0
	10–25	3
	> 25	5
Obesity (BMI > 30)		1
Ventilation		4
Lower extremities fracture		2

According to the risk scores, the indication to VTE prophylaxis should be carefully evaluated. Low risk patients may not require VTE prophylaxis, while high risk patients should receive it. However, some critical conditions such as active bleeding, coagulopathy, hemodynamic instability, solid organ injury, traumatic brain injury or spinal trauma may need a delay of VTE pharmacological prophylaxis until stabilization. In these cases, mechanical prophylaxis (intermittent pneumatic compression, elastic stockings or mobilization) should be applied instead, if possible [403–406].

The large scale Norwegian national prospective observational study including 45.000 elderly patients undergoing osteosynthesis for hip fracture compared pre-operative and post-operative start of VTE prophylaxis. Pre-operative prophylaxis did not influence mortality or risk of reoperation in patients treated with osteosynthesis. However, post-operative prophylaxis decreased the risk of intraoperative bleeding complications for operations with hip compression screw, but not with intramedullary nail or screw osteosynthesis [405].

The Cochrane systematic review by Barrera et al. recommends prophylaxis to reduce the risk of DVT in severe trauma patients (RR 0.52). Although mechanical prophylaxis is effective in reducing the risk of DVT (RR 0.55), pharmacological prophylaxis seems more effective (RR 0.48), even if it may increase the risk of bleeding (RR 2.04). Low molecular weight heparin (LMWH) is preferable over unfractioned heparin (UFH) due to higher effectiveness in preventing DVT (RR 0.68). The association of mechanical and pharmacological prophylaxis further decreases the risk of DVT (RR 0.34). Neither mechanical nor pharmacological prophylaxis seem to reduce the risk of pulmonary embolism (PE) [387]**.**

The retrospective cohort study by Campbell et al. based on a national database including 4000 elderly patients (> 60 years old), evaluated the effectiveness of factor XaI inhibitors compared to LMWH to prevent DVT after hip fracture surgery. Data did not show significant differences in DVT, bleeding or post-operative transfusions rates. However, the rate of PE was significantly higher in factor XaI inhibitors than LMWH (AR 2 vs -3.5). The authors concluded that factor XaI inhibitors may be a viable alternative to LMWH in view of patient’s preference and better compliance, despite their increased cost and lower efficacy in preventing PE [406].

The retrospective propensity score matching study of the American College of Surgeons Trauma Quality Improvement Program compared the use of UFH and LMWH in 40,000 elderly trauma patients (> 65 years old) to evaluate the risk of bleeding. LMWH was associated with a lower incidence of DVT (*P* = 0.007) and PE (*P* < 0.001), fewer bleeding complications and transfusions, *P* < 0.001, surgical procedures (*P* = 0.007), myocardial infarction (*P* < 0.0001), cardiac arrest (*P* = 0.001), severe sepsis (*P* < 0.001) and mortality (*P* < 0.001). Subanalysis by age group confirmed the lower rate of VTE (*P* = 0.003) and bleeding complications among patients ages > 75y receiving LMWH. Differences were more evident in ISS < 16. The authors conclude that LMWH is superior to UFH at preventing VTE events with fewer bleeding complication and should be therefore the drug of choice in most elderly patients with ISS > 16 [407].

The initial dose of LMWH enoxaparin for patients older than 65 years should be 30 mg every 12 h**.** In case of renal failure, UFH should is (5000 U every 8 h)**.** Heparin dose adjusted according to anti-Xa levels improved the efficacy of VTE prophylaxis without increasing the rate of bleeding complications [408–410].

## Management of the end of life in trauma setting for elderly patients

**Key Question 6.1**

**Which are the clinical features and vital signs to define “end of life” in the elderly trauma patient?**

**Statement 6.1.1**

There are no defined clinical features and vital signs to establish the elderly patient at end of life in trauma setting [QoE D very low].

**Statement 6.1.2**

Age alone is not an indication to withhold aggressive therapy [QoE C low].

**Statement 6.1.3**

To define the end of life in elderly patient in trauma setting is a very complex and delicate process. It should consider prognosis in regard to survival outside the acute care setting, the recovery of cognitive ability sufficient to perceive the benefits of treatment, the ability to resume physical activities, the patient’s advance directives, and the involvement of the surrogate decision-maker or healthcare proxy and of the family [QoE D very low].

**Recommendation 6.1**

We recommend discussing in a multidisciplinary approach the end of life in an elderly patient in the trauma setting. The decision should be considering the patient’s directives, family feelings and representatives’ desires and should be shared **[Strong recommendation based on a low-very low quality of evidence 1D].**

### Summary of evidence and discussion

Withholding and withdrawing life support of elderly traumatic patients is a common occurrence in ICU, but, unfortunately, the futility of continued care and the definition of the end of life of a patient is not always obvious. Evidence shows that long-term functional outcomes of elderly trauma patients who survive their injuries can be good [411]**.** In a large retrospective study on 38,707 patients > 65 years old, 50% of the survivors were discharged to home [412] and in another study the 83% of patients ages 75 and older, who survived for 4 years after injury were living in an independent setting [413]. Available data indicate that age alone is not an indication to withhold aggressive therapy. Moreover, neither the perceived suffering of geriatric patients nor a poor anticipated quality of life should be used as the only criterion for withdrawal of support. In fact, most surgical ICU patients who survive, indicate that they would repeat the experience again if necessary [414] and that they have an “acceptable” quality of life and would undergo treatment again [415].

When determining whether ICU interventions are futile or not, clinicians must establish the prognosis in regard to survival outside the acute care setting and recovery of cognitive ability sufficient to perceive the benefits of treatment [416]. Such prognostication, however, can be difficult. Several scoring systems have been created and validated to predict in-hospital mortality of traumatic elderly patients. The **Geriatric Trauma Outcome Score (GTOS)** includes ISS, age, performance of packed red blood cell transfusion within 24h of admission as variables. It was externally validated on 18,282 subjects between the ages of 65 years and 102 years [59–61]**.** GTOS and Trauma Injury Severity Score (TRISS) were found to perform similarly and accurately in predicting the probability of death for injured elders. GTOS has the advantage of having fewer variables to be collected, and no reliance on data collected in the emergency room (ER) or by other observers, such as physiologic data. The AUC for GTOS ability to predict mortality in injured elders is 0.844 (95% CI 0.837–0.851) [60, 61].

The **quick Elderly Mortality After Trauma (qEMAT)** includes systolic blood pressure, pulse, GCS, presence of penetrating injury, story of congestive heart failure, chronic renal failure, and cirrhosis. It was retrospectively validated on 243,270 patients > 65 years old with an AUC of 0.87 (95% CI 0.86–0.87) for prediction of in-hospital mortality. This method outperforms GTOS, TRISS and age plus ISS [417]. The **Score for Trauma Triage in the Geriatric and Middle-Aged (STTGMA score)** includes age, Glasgow Coma sale (GCS), mechanism of injury, AIS sub-scores for head and neck (AIS-NH), and pelvis and extremity body regions (AIS-EXT), Charlson Comorbidity Index. It was prospectively validated for in-hospital mortality and for death 48h from admission on 1470 patients. The AUC for the STTGMA score ability to predict death within 48h from admission was 0.943 (95% CI 0.886–0.999) [418–420]. The **modified 15 variable Trauma-Specific Frailty Index (TSFI)** was retrospectively validated on 200 patients with age > 65 years presenting to a level 1 trauma center for predicting unfavorable discharge disposition (discharge to skilled nursing facility or death). The area under the curve was 0.829 [0.774–0.884]. Geriatric trauma patients with a TSFI cut-off score of > 0.27 are more likely to have unfavorable discharge disposition [67]. Other scores were created to predict the long-term prognosis and quality of life of these patients after discharge. The **Palliative Performance Scale (PPS)** derived from assessment of 5 domains: ambulation, activity level/evidence of disease, self-care, intake, level of consciousness. PPS has been initially shown to be correlated with survival in patients with advanced cancer. Then, it was prospectively validated for the ability to predict mortality and poor outcome at discharge and after 6 months in > 54 years old trauma patients. Low PPS patients fail to improve over time compared to high PPS patients [421, 422]. The **FRAIL Questionnaire** assesses five components: Fatigue, Resistance, Ambulation, Illnesses, and Loss of weight. It was prospectively validated on 188 patients > 65 years old admitted through the emergency department with a primary injury diagnosis. The FRAIL Questionnaire predicts 1-year functional status and mortality after trauma in patients > 65 years old and is a useful tool for bedside screening [423]. These scores could be used on admission for prognostication of short and long-term outcomes, and they could help in the management of these patients. However, there is no adequate level of evidence to recommend the routine use of these scores as potential trigger for palliative care in older trauma patients.

Focusing on brain injuries, patients aged above 65 years suffering traumatic brain injury have double in-hospital mortality compared to those younger than 65, and among the survivors elderly patients have higher healthcare utilization and worse early and long-term outcomes [424–426]. Western countries’ population is progressively aging and management of severely injured patients has drastically improved in the last decades. This leads to more patients surviving initial resuscitation and inevitably to the greater need of quality end-of-life care and palliative care. In this setting, the appropriate management of traumatic brain injury among elderly has become a public health requirement. The identification of patients whose therapeutic chances are reduced and who will least benefit from aggressive treatment becomes decisive to direct therapeutic efforts and to reduce medical futility. The Eastern Association for the Surgery of Trauma (EAST) guidelines and the American College of Surgeons Trauma Quality Improvement Program (ASC-TQIP) emphasized the importance of evaluating clinical improvement in the first 72 h (20). Severe trauma brain injury was defined as Glasgow Coma Scale (GCS) score 8 (20), and failure to improve in GCS within 72 h from the start of treatment is a negative prognostic factor associated with poor functional outcome or death despite aggressive treatment [427]**.** Patients who do not show signs of improvement within 72 h should be carefully evaluated before undergoing further aggressive treatment. The first 72 h constitute the critical interval to determine the prognosis. Unfortunately, the guidelines do not specify the parameters useful to quantify a neurological improvement although the persistence of a comatose state (GCS 8) at 72 h is certainly associated with a poor prognosis. It is evident that this time interval is absolutely arbitrary but certainly it represents the minimum time to assess the chances of survival and the effectiveness of the initial interventions. Age, *per sè*, is not considered a valid reason to limit the treatments available or to lead the decision to withdraw active treatment whilst frailty is a superior predictor of poor outcome [428–431].

A retrospective study of patients older than 65 admitted a Level I Trauma Center with severe brain injury (GCS >  = 8) documented that mortality was significantly higher in patients that do not show improvement in GCS score at 72 h. However, this significant difference was not recorded in the functional status at discharge and in the 12-month survival. Improvement to treatment at 72 h was not associated with better functional status and with better long-term survival. Then, among elderly, neurological status at 72 h is a good prognostic factor for in-hospital death but is not a valid tool to predict long-term outcomes for survivors [431].

**Key Question 6.2**

**Could palliative management be useful in the management of an elderly patient at the end of life?**

**Statement 6.2.1**

During the management of an elderly severely injured patient, the early insertion in the decision-making process of palliative medicine consultation improves outcomes, reduces in-hospital mortality and length of stay and improves communication with family, avoiding unnecessary operation [QoE C low].

**Statement 6.2.2**

Improved palliative care skill training for surgeons should be necessary to be more competent in end-of-life decisions [QoE D very low-quality].

**Recommendation 6.2**

We recommend involving as soon as possible the palliative care team in managing an elderly severely injured patient at the end-of-life status **[Strong recommendation based on a low-very low quality level of evidence 1C].**

### Summary of evidence and discussion

In the last twenty years there has been an increase of the elderly population (older than 65 years) rate among hospital trauma-related admissions. These patients require almost the 25% of trauma-related health care resources due to their comorbidities and the decrease of physiologic reserve with high mortality [432–435]. The National Institutes of Health has estimated that the 5% of the most seriously ill Americans accounted for more than 50% of health care spending, with most costs occurring during the last 6 months of a patient’s life [430]. Racial and socioeconomic disparities in terms of utilization of hospice services were reported [433]. In US, Asian, African American, and Hispanic patients received less hospice care than Caucasian patients (OR 0.65, 0.60, 0.73; *P* < 0.0001). Race and ethnicity are independent predictors of a trauma patient’s transition to hospice care and significantly affect the length of stay [433]. However palliative management can be beneficial in the management of elderly injured patient. In such cases, when a patient sustains severe injuries that are unlikely to be fully recoverable, palliative care, including pain and symptom management, emotional and psychological support for the patient and his/her family, facilitating open and honest communication between the patient, their family and healthcare providers, can provide essential support and focus on the patient's comfort and quality of life, preserving the patient's dignity, comfort and enhancing their quality of life, regarding life-sustaining treatments and interventions. Palliative care teams collaboration in the setting of the end of life ensure that the patient's wishes regarding their care are known and respected, even if they become unable to communicate them later on [434, 435].

Palliative management in the context of an elderly injured patient at the end of life after trauma aims to provide holistic support, alleviate suffering, and improve the patient's overall well-being during their remaining time. It complements the efforts of the trauma team by focusing on comfort, dignity, and quality of life, while also supporting the patient's family throughout the process [434, 435].

Emergency and trauma surgeons have the responsibility to take important decisions in the end of life setting and in extreme situations; they need to be supported by the palliative care team, to respect the patient’s and family directives, share this decision and communicate it in the proper way.

End-of-life decision making is a variable process that involves prognosis, predicted functional outcomes, personal beliefs, institutional resources, societal norms, and clinician experience. An international survey showed that **t**he admitting surgeon guided most end-of-life decisions, that formal medical futility laws are rarely available, that ethical consultation services are often accessible but rarely used, and typically unhelpful. Therefore the decision depends on type of injury, different religions, decision-maker viewpoint, and institutional resources and results in significant variation after trauma [436]. Palliative care physicians are more familiar and have more training in this complex decision making [437]. Moreover trauma surgeons can identify early patients who could die because of traumatic injuries, but they have the difficulty to estimate long-term outcomes [438]**.** It is crucial to highlight that palliative care is not only for patients at the end-of-life but is an approach that try to improve patient and their family’s quality of life and outcomes in case of life-threatening illness [438–440]. Despite these considerations and the growing importance of this topic, Ball et al. reported that palliative care consultation is underused. The most frequent barriers for patients and families to consultation are the resistance by families (40.2%), the concern for patient and family feeling that doctors are "giving up" (30.4%), the miscommunication regarding prognosis or diagnosis (27.4% and 16.2% respectively)**.** Smoothing out these barriers could greatly increase treatments and outcomes in these category of patients [436].

Aziz et al. [439] carried out a systematic review to assess if geriatric trauma patients should receive post-injury care in a trauma center or not and if they should receive routine palliative care processes. They showed that for this group of patients, trauma center care was associated with improved outcomes in most studies and that the utilization of early palliative care consultations was generally associated with improved secondary outcomes, such as length of hospital stay. A study by Baimas-George et al. [432], based on a very large cohort of more than 16.000 emergency surgical patients who received palliative cares, showed that involving palliative care systems may be useful to decrease suffering, improve outcomes, and reduce non-beneficial and unwanted care. In this study 4% of patients were classified with an end-of-life disease (ELD): 3% received palliative care services, 5% were discharged to hospice, and 22% had an inpatient mortality. Authors reported that controlling for patient characteristics, utilization of palliative care services was associated with increased odds of discharge to hospice compared to inpatient mortality (OR 1.78 all patients and OR 2.04 for ELD).

In a retrospective study by Hoffman et al., 86.7% of trauma patients who underwent Withdrawal of Life-Sustaining Treatment after surgery received a palliative care consult. However, the short time between surgery and withdrawal of life-sustaining treatments suggested that increase palliative care system before surgery can help in decision-making process to avoid unnecessary surgeries [437].

Davies et al. in a monocentric retrospective study analyzed the impact of palliative care in management of femur fracture in high-risk patients [441]**.** The results confirmed that an early (24–72 h) intervention of palliative care is successful, also if mortality rates remain high. Similar conclusions were reported by Schuijt et al. [442], who found that a patient-tailored treatment associated with a decision-making multidisciplinary team can be helpful. The decision making rely on discretion to identify patients most appropriate for palliative management.

Stonko et al. reviewed data from a national trauma register including 614,496 geriatric trauma patients to assess if failure to rescue rate from any complication worsens with age and injury severity and reported that patients with complications tended to be older, female, non-white, have non-blunt mechanism, higher ISS, and hypotension on arrival. Overall mortality was highest (19%) in the oldest (≥ 86 years old) and most severely injured (ISS ≥ 25) patients and the occurrence of any complication was an independent predictor of overall mortality in geriatric patients (OR 2.3; 95% CI 2.2–2.4) [349].

Moreover sarcopenia is the strongest predictor of out-of-hospital mortality among older adults who sustained a fall (HR 4.77) [443]. Early diagnosis of sarcopenia allows the detection of trauma patients who are at high risk for adverse events. In geriatric blunt trauma patients, sarcopenia was associated with increased in-hospital mortality (OR 1.61), had a higher risk of discharge to less favourable destinations (OR 1.42) and had an increased risk of prolonged hospitalization (HR 1.21) [444].

There is a need for palliative care education for many specialists that are often still reluctant to palliative care. The utilization of palliative care is very low in this subset of patients: only 35% of patients with severe trauma brain injury receive palliative care. The available data show that palliative care is associated with less intensity of care, higher quality of life at the end of life and shorter length of stay for survivors. Integration of palliative care in the management of severe trauma brain injury patients definitively improves quality of care without reducing survival [445–448]. In conclusions, the implementation of palliative medicine consultation in the decision-making process at the hospital admission or in the first 24–72 h for geriatric trauma patients improves outcomes, reduces in-hospital mortality, length of stay and improve communication with family members [443–445, 449]**.**

## Conclusions

By the time, with the improvements of quality of life and care, elderly trauma patients (≥ 65 years old) are increasing. Trauma in elderly people has high mortality. The WSES decided to provide guidelines focused on the management of this group of co-morbid and frail patients and based on available evidence and on experts’ opinion, to improve elderly trauma patients’ care and decrease their mortality. The management of elderly trauma patients requires knowledge and understanding of ageing physiology and a multidisciplinary approach. Ageing is correlated with frailty and frailty is a risk factor for mortality. Focused triage and early activation of tailored trauma protocols for elderly trauma patients can improve their resuscitation, according to their different physiology and response to trauma stress. Early involvement of palliative care teams and shared decision making in assessing elderly injured patients can decrease futility, improve communication, outcomes and quality of life. Finally, geriatric ICUs are needed to care for elderly trauma patients using a multidisciplinary approach.

### Supplementary Information

**Additional file 1**. List of statements and recommendations.

## Data Availability

Supplemental materials are available.

## Abbreviations

CT: Computed tomography
WSES: World Society of Emergency Surgery
DOACs: Direct oral anticoagulants
VKAs: Vitamin K antagonists anticoagulants
LMWH: Low molecular weight heparin
UFH: Unfractionated heparin
GTOS: Geriatric Trauma Outcome Score
TSFI: Trauma Specific Frailty Index
NSAID: Non steroidal anti-inflammatory drugs
ISS: Injury Severity Score
TESS: Trauma embolic scoring system
VTE: Venous thromboembolism
