# Supplementary material for: The 2023 WSES guidelines on the management of trauma in elderly and frail patients
Source: World J Emerg Surg. 2024 May 31;19:18. doi: 10.1186/s13017-024-00537-8 (PMC11140935; doi:10.1186/s13017-024-00537-8)
Supplement: Supplementary file 1 — Additional file 1. List of statements and recommendations. [file 13017_2024_537_MOESM1_ESM.docx]

**DEFINITIONS**

**Key Question 1.1 :**

***Which trauma patient is defined as “old” at initial evaluation?***

**Statement 1.1.1**

The chronological age does not correspond to the biological age. Aging is correlated with para-physiological changes in organ systems with altered response to trauma, compared with younger injured patients [QoE MODERATE B].

**Statement 1.1.2**

Patients aged ≥ may require dedicated trauma care, because they may have high mortality rates after trauma [QoE LOW C].

**Statement 1.1.3**

The age of 65 is most often used referring to “old”, “elderly” or “geriatric” patients [QoE HIGH A].

**Recommendation 1.1**

We suggest early trauma protocol activation in patients aged ≥ 55 years old [Weak recommendation based on a low level of evidence 2C].

We recommend to carefully evaluate injured patients aged ≥ 55-year-old for potential high risk of mortality and to avoid under-triage [Strong recommendation based on a low level of evidence 1C].

**Key Question 1.2:**

***When is a patient considered “physiologically old” and does he/she deserve different management after (blunt or penetrating) trauma?***

**Statement 1.2.**

Frailty, hearth diseases, hepatic diseases, renal diseases, and cancer according to their stage and severity are risk factors for mortality in trauma patients [QoE low C].

**Recommendation 1.2**

We suggest an early and rapid assessment of the patient including vital signs on presentation, mechanism of injury, injury severity and frailty including comorbidities and medication history to identify vulnerable trauma patients **[Weak recommendation based on low level of evidence 2C].**

We recommend assessing frailty in all elderly trauma patients **[Strong recommendation based on a moderate level of evidence 1B].**

**PRIMARY EVALUATION/ASSESSMENT**

**Key Question 2.1:**

***Which injury (physiological and anatomical) scores are stronger predictors of outcome in evaluating elderly patients for trauma?***

**Statement 2.1.1**

Geriatric trauma patients are usually under-triaged to trauma centers due to low energy mechanisms of injury, unreliability of vital signs, and the use of medications that can obscure the physiologic response to trauma. Specific triaging scores can be used to predict outcomes in geriatric trauma patients and guide the triage decision-making process towards transfer to a Level I trauma centers and aggressive treatment (QoE moderate B).

**Recommendation 2.1**

We suggest evaluating elderly patients for trauma through the Geriatric Trauma Outcome Score (GTOS) to predict in-hospital mortality and the Trauma-Specific Frailty Index in order to identify patients at highest risk of poor outcome **[Weak Recommendation, based on Moderate Quality of Evidence, 2B]**.

**Key Question 2.2:**

***Which clinical features do better define the hemodynamic instability in geriatric trauma patients?***

**Statement 2.2.1**

Most geriatric patients have hypertension, cardiovascular disease, and impaired sensitivity to catecholamines. They can be on chronic medications such as beta-blocker therapy that can affect heart rate and blood pressure, blunting the systemic response to injury and significant blood loss with the absence of early tachycardia (QoE B moderate).

**Statement 2.2.2**

Geriatric patients should have appropriate assessment of their poly-pharmacologic profile as soon as possible after admission. They should be screened for beta-blockers, steroids, antiplatelet and anticoagulant medications. The frequent use of anticoagulant (warfarin, coumadin, dabigatran, rivaroxaban) and antiplatelet (clopidogrel, aspirin) medications in the geriatric population, puts these patients at high risk for significant bleeding events, even after minor trauma (QoE B moderate).

**Recommendation 2.2**

We recommend keeping a lower threshold for trauma protocol activation in geriatric patients, with triage set points of heart ratio 90 bpm and systolic blood pressure less than 110 mmHg **[Strong Recommendation, based on Moderate Quality of Evidence, 1B].**

**Key Question 2.3:**

***Which laboratory tests and biological markers are useful to evaluate the elderly trauma patient before resuscitation?***

**Statement 2.3.1**

Occult hypoperfusion is often under-estimated in the geriatric trauma patient. A prompt assessment of base-deficit and lactates levels should be performed to identify those patients who need resuscitation and admission to an ICU. Elevated lactate and base deficit are definitely strong predictors of mortality within 24 hours from hospital admission (QoE moderate).

**Recommendation 2.3**

We recommend performing an early blood gas (arterial or venous) for baseline base-deficit or a lactic acid assessment in geriatric trauma patients **[Strong Recommendation, based on Moderate Quality of Evidence, 1B]**.

**Key Question 2.4:**

***Which imaging studies are useful to better evaluate trauma elderly patients?***

**Statement 2.4.1**

Geriatric patients show injury patterns that differ considerably from those seen in the younger population. They are prone to serious injuries after relatively minor trauma because of overall frailty, comorbidity, and medication effects. Early diagnosis and aggressive intervention can decrease mortality and enable geriatric patients to return to independent living (QoE B moderate).

**Recommendation 2.4**

We recommend a low threshold for initial imaging with CT scan in geriatric trauma patients. The diagnostic yield of a contrast-enhanced CT outweighs the risk of contrast-induced nephropathy, especially in view of the potential, dramatic effects of under-triage **[Strong Recommendation, based on Moderate Quality of Evidence, 1B]**.

**RESUSCITATION**

**Key Question 3.1:**

***What early resuscitative protocol including intravenous fluids, blood transfusions or vasopressors should be used to manage geriatric trauma patients at primary evaluation?***

**Statement 3.1.1**

Available data do not recommend a specific early resuscitative protocol over another in geriatric trauma management. (QoE D very low)

**Statement 3.1.2**

Resuscitative protocols for elderly trauma patients aim to early identification of tissue hypoperfusion, and rapid treatment of coagulopathy, hypovolemia, and traumatic injury to improve outcomes and decrease mortality (QoE B moderate)

**Statement 3.1.3**

In the elderly trauma patient, the resuscitative strategy should be individualized and tailored according to clinical history, comorbidities, concomitant medications, clinical and laboratory findings, and treatment response. (QoE B moderate)

**Statements 3.1.4**

In elderly trauma patient, close monitoring and frequent repeated measurements of vital signs trend and gas analysis are likely to be more useful than any individual measurement to guide the resuscitative strategy (QoE C low).

**Recommendation 3.1**:

We recommend that every trauma center provides meticulous triage criteria to recognize the need to early activate resuscitative protocols for elderly patients. These triaging criteria should include physical examination, vital signs, blood gas analysis, and medical history, emphasizing clinical conditions and drug history that may guide resuscitative therapies, early coagulative support, and the need to correct coagulopathies, and minimise fluids **[Strong recommendation based on moderate quality of evidence 1B].**

We recommend rapid recognition and correction of coagulation disorders related to trauma or chronic medication intake in elderly patients**. [Strong recommendation based on moderate quality of evidence 1B]**

We recommend performing serial base deficit assessment and lactate levels as markers of occult hypoperfusion in addition to close monitoring of vital parameters trend (heart rate, blood pressure, respiratory rate, urinary output), and mental status in elderly patients in a dedicated intensive geriatric care unit **[Strong recommendation based on moderate-low level quality of evidence 1B]**.

We suggest considering carefully to administer inotropic agents in selected non-responding elderly patients to target resuscitation **[Weak recommendation based on low level of evidence 2C].**

**Key Question 3.2:**

***Which are the resuscitation endpoints in elderly trauma patients?***

**Statement 3.2.1:**

In the elderly trauma patient, normotension and the absence of tachycardia and tachypnea do not

Rule out tissue hypoperfusion. (QoE A-B strong-moderate)

**Statement 3.2.2:**

There is no evidence that one type of invasive hemodynamic monitoring is more efficient than another in elderly trauma management; the indication for hemodynamic monitoring should be evaluated according to the patient's clinical features and the team's expertise. (QoE B-C moderate-low)

**Statement 3.2.3:**

The adoption of Point-of-Care Ultrasound (POCUS) in the resuscitation of the elderly severely injured patient may be an effective tool in monitoring the hemodynamic status of the patient as it provides information on blood volume and cardiac function in a rapid, cost-effective manner, without the side effects of invasive monitoring systems. (QoE B-C moderate-low)

**Recommendation 3.2:**

We recommend evaluating the indication for invasive versus non-invasive hemodynamic monitoring on a case-by-case basis in injured elderly patients. Hypoperfusion should be ruled out by serial base deficit assessments and lactate concentration **[Strong recommendation based on moderate-low level of evidence 1B].**

We suggest the implementation of POCUS in monitoring the cardiac function and blood volume in elderly injured patient, if skills are present. Invasive hemodynamic monitoring should be reserved in selected cases, to critically ill elderly trauma patients who have hypotension, significant injuries (as defined by an Abbreviated Injury Score >3 or a Trauma Score <15), or uncertain cardiovascular and/or fluid status **[Weak recommendation based on moderate and low level of evidence 2B].**

**Key Question 3.3**

***Which vasopressors are indicated in comorbid elderly injured patients?***

**Statement 3.3.1**

The use of a vasopressors before volume replacement may be deleterious in all trauma patients (QoE A strong).

**Statement 3.3.2**

The use of vasopressors is indicated in trauma patients who do not respond to early fluids in the context of damage control resuscitation and permissive hypotension (QoE B moderate).

**Statement 3.3.3**

In trauma patients not responders to early resuscitation with hypotension refractory to volume filling, and with hypotension of neurogenic and septic origin, the vasopressor of choice is norepinephrine (QoE A strong).

**Statement 3.3.4**

In an elderly trauma patients, it is appropriate to consider the administration of an inotrope in case of non-response or in case of hypotension due to cardiac dysfunction (QoE C low).

**Statement 3.3.5**

Dobutamine may be helpful in selected elderly trauma patients presenting with shock-related to heart failure, bradycardia from cervicothoracic myelic injury, and cardiac contusion (QoE B-C moderate-low).

**Recommendation 3.3**

We recommend against the routine use of vasopressors in elderly injured patients presenting with hypotension caused by hemorrhage **[Strong recommendation based on high-moderate level of evidence 1A].**

We recommend identifying the cause of hypoperfusion and assessing preexisting conditions and pharmacologic history before choosing a vasopressor in managing trauma in an elderly patient **[Strong recommendation based on a high-low quality level of evidence 1A].**

We suggest using norepinephrine in elderly patients suffering from neurogenic shock. The dose to be used must be the lowest to guarantee tissue perfusion. The possible onset of cardiac arrhythmia and possible hypotensive effects should be monitored **[Weak recommendation based on a moderate-low quality level of evidence 2B].**

**Key Question 3.4**

***Vasopressors treatments versus permissive hypotension in geriatric trauma patients: which are the clinical parameters and laboratory tests to consider for choice?***

**Statement 3.4.1**

The administration of vasopressors versus permissive hypotension debate during the early resuscitative stage of elderly trauma patients remains unresolved. Management should be individualized according to the mechanism of trauma, the patient's acute and chronic conditions, and frailty (QoE B-C moderate-low)

**Recommendation 3.4**

We recommend to carefully evaluate to implement permissive hypotension in managing selected elderly trauma patients. Tissue perfusion has to be constantly monitored by base excess level, arterial lactate dosage, urine output, and when possible, neurologic assessment. **[Strong recommendation based on a high-low quality level of evidence 1A].**

**Key question 3.5:**

***How intraoperative hypotension status is correlated with delirium in geriatric patients?***

**Statement 3.5.1:**

There is evidence correlating the occurrence of postoperative delirium and perioperative hemodynamic changes (QoE C low)

**Recommendation 3.5**

We suggest assessing, as early as possible, the risk factors for the onset of delirium because it is related to unfavourable outcomes in trauma geriatric patients. [**Weak recommendation based on a moderate-low quality level of evidence 2B]**

**MANAGEMENT OF ORAL ANTICOAGULANTS**

**Key question 4.1:**

***Which blood tests are useful to evaluate geriatric patients on anticoagulant drugs in trauma setting?***

**Statement 4.1.1:**

When assessing the risk of bleeding in the emergency setting, the type of anticoagulant ingested, time of ingestion, age, renal and hepatic function must be evaluated (QoE A high).

**Statement 4.1.2:**

Prothrombin Time and INR are a reliable methods to assess clinically relevant exposure to oral vitamin K antagonists anticoagulants (QoE A high).

**Statement 4.1.3:**

Due to the low sensitivity and specificity of the Prothrombin Time (PT) and Activated Partial Thromboplastin Time (aPTT) to direct oral anticoagulants (DOACs), they are not reliable to assess DOACs activity, especially apixaban and endoxaban [QoE A high].

**Statement 4.1.4:**

A normal Thromboplastin Time (TT) suggests that there is no clinical relevant Dabigatran activity(QoE B moderate).

**Statement 4.1.5:**

Although viscoelastic testing including TEG or ROTEM has been advocated to guide the identification of trauma coagulopathy, it’s role in elderly patients on anticoagulant therapy is not established (QoE B-C moderate-low).

**Statement 4.1.6:**

More reliable and faster qualitative and quantitative tests of coagulation such as calibrated drug-specific anti-Xa levels should be strongly considered in the workup of geriatric trauma patients (QoE B-C moderate-low).

**Statement 4.1.7**

Anti-Xa assay is the gold standard for monitoring Low Molecular Weight Heparin (LMWH) therapy (QoE A high).

**Statement 4.1.8**

Quantitative assays, such as ecarin clotting time (ECT), dilute thrombin time (dTT), and anti-Xa inhibitor, which can accuracy determine DOACs plasma concentrations, are not readily available in all hospitals. They can be utilized, if the results don’t require urgency, as second-line tests (QoE B-C moderate-low).

**Recommendations 4.1**

We recommend performing routinely the common coagulation assays in elderly patients including the Activated Partial Thromboplastin Time (aPTT), Thromboplastin Time (TT), Prothrombin Time (PT), INR, and anti-Xa levels to assess early anticoagulants exposure in the trauma setting. There is not enough evidence to support the routinely use of TEG or ROTEM in elderly trauma patients. Further studies are necessary to determine their role. **[Strong recommendation based on a moderate level quality of evidence 1B]**

**Key question 4.2:**

***Which reversal protocol is indicated in patients being treated with vitamin K antagonists?***

**Statement 4.2.1**

Oral vitamin K antagonists reversal protocol is indicated in elderly patients presenting with hemorrhagic shock not responding to supportive measures or needing for an urgent surgery or invasive procedure (QoE A high).

**Statement 4.2.2**

Anticoagulated patients with oral vitamin K antagonists (VKAs) presenting with head trauma but without radiographic evidence of Intracranial hemorrage (ICH) or other bleedings should not receive preventive reversal (QoE B-C moderate-low).

**Statement 4.2.3**

If an emergency surgical exploration is indicated and it can be delayed for 6–12 hrs, in trauma elderly patient with a history of VKAs treatment, the INR can be corrected by administrating intravenous vitamin K, in selected cases (QoE A-B high-moderate).

**Statement 4.2.4**

For surgery that requires reversal of oral vitamin K antagonists, and which cannot be delayed for vitamin K to have time to take effect, the INR can be corrected by giving prothrombin complex concentrate (PCC) and intravenous vitamin K. PCC should not be used to enable elective or non-urgent surgery (QoE C low).

**Statement 4.2.5**

Oral vitamin K antagonists (VKAs) reversal agents should be managed according to INR level (QoE B moderate).

**Statement 4.2.6**

PCC, preferably intravenous (4F-PCCs), is strongly recommended for prompt oral vitamin K antagonistsVKAs reversal. If those are not available Fresh Frozen Plasma (FFP), III factor Prothrombin Complex (3PCC), or recombinant FVIIa could be used (QoE B moderate).

**Statement 4.2.7**

Vitamin K administration alone is not recommended as a reversal agent in patients with life-threatening bleeding, but as an adjunct treatment in these patients (QoE A-B high-moderate).

**Statement 4.2.8**

The use of Recombinant activated factor VII (rFVIIa) as VKAs reversal agent increases the risk of thromboembolic events, especially in elderly patients (QoE B moderate).

**Recommendation 4.2:**

We recommend administrating a reversal agent in elderly trauma patients anticoagulated with oral vitamin K antagonists who present with bleeding, not responding to supportive measures, major life-threatening bleeding, bleeding located in critical organs (central nervous system, abdominal, thoracic), or needing urgent surgical or invasive procedures **[Strong recommendation based on a moderate level quality of evidence 1B].**

We recommend to use the reversal protocol including intravenous factor prothrombin complex concentrates (4F-PCCs) and 5 mg intravenous vitamin K in case of life-threatening bleeding and/or urgent surgical procedures. Further doses should be administered if needed to achieve INR <1.5 **[Strong recommendation based on a high level quality of evidence 1A].**

We recommend giving Fresh frozen plasma (FFP) as oral vitamin K antagonists (VKAs) agent reversal only if no other treatment is available **[Strong recommendation based on a moderate quality level of evidence 1B].**

We do not recommend the use of recombinant activated coagulation factor VII (rFVIIa) as first-line VKA reversal agent **[Strong recommendation based on a low level of quality evidence 1C].**

**Key Question 4.3:**

***Which reversal protocol is indicated in patients in treatment with direct oral anticoagulants (DOACs)?***

**Statement 4.3.1**

In deciding if it is necessary to proceed with active reversal of direct oral anticoagulants (DOACs) in trauma setting, it is crucial to assess DOAC plasma concentration, in fact the administration of a reversal agent is useful only when the anticoagulant drug is active in patient's plasma in measurable quantities but only few centres have the DOAC mesurement available (QoE A-B high-moderate).

**Statement 4.3.2**

In general clinical practice, it is recommended to consider anticoagulant DOAC reversal for patients with serious bleeding and a DOAC level >50 ng/mL, and for patients requiring an invasive procedure with high bleeding risk and a DOAC level >30 ng/mL (QoE A high).

**Statement 4.3.3**

The main DOACs reversal agents are the idarucizumab (Praxbind*®*) for reversal of dabigatran and the andexanet alfa for reversal of apixaban and rivaroxaban (QoE A high).

**Statement 4.3.4**

Andexanet alfa is not indicated for DOACs reversal in patients requiring urgent surgery (QoE C low).

**Statement 4.3.5**

If a DOAC-treated patients requires an invasive procedure, the active reversal is indicated only if the procedure cannot be safely performed while the patient is anticoagulated, cannot be delayed, and there is demonstration or reasonable expectation that the patient has clinically relevant plasma DOAC levels (QoE B-C moderate-low).

**Recommendation 4.3**

We recommend an early assessment of laboratory coagulation tests and direct measurements of DOAC levels, if quantitative tests are available, in elderly trauma patients receiving or suspected of having received a DOAC before deciding for reversal due to the thromboembolic risk **[Strong recommendation based on a moderate level quality of evidence 1B].**

We suggest the administration of DOACS reversal agents only in critically ill patients with dosable plasma DOAC levels and presenting with hemorrhagic shock not responding to resuscitation, when level of DOACS can be assessed **[Weak recommendation based on a moderate-low quality of evidence 2B].**

If the trauma patient with uncontrolled life-threatening bleeding, was treated with dabigatran (anti-FIIa activity), the suggested reversal protocol is to administer idarucizumab 5 g IV. If idarucizumab is not available, 50 units/kg IV of activated prothrombin complex concentrates (APCC) may be administrated **[Weak recommendation based on a moderate-low quality level of evidence 2B].**

In patients with rivaroxaban-associated or apixaban-associated (FX inhibitors) life-threatening and uncontrolled bleeding, the suggested reversal protocol is the administration of andexanet alfa as an intravenous bolus of 400 mg over 15 min followed by a continuous infusion of 480 mg over 2 h (low dose) or 800 mg over 30 min followed by a continuous infusion of 960 mg over 2 h (high dose), according to the last dose of DOAC and the size of the dose. If andexanet alfa is not available, 2000 units of four-factor prothrombin complex concentrates (PCC) may be administrated **[Weak recommendation based on a moderate-low quality level of evidence 2B].**

**ANTIBIOTICS, ANALGESIA AND ANTI-THROMBOTIC PROPHYLAXIS**

**KEY QUESTION 5.1:**

***When is it indicated to administer antibiotics in elderly trauma patients?***

**Statement 5.1.1**

Antibiotic prophylaxis including a single pre-operative narrowest spectrum antibiotic dose covering aerobic and anaerobic bacteria, is commonly administered after penetrating abdominal trauma in all patients before surgical exploration (QoE C low).

**Statement 5.1.2**

If an exploratory laparoscopy/laparotomy is required, in case of peritoneal cavity contamination due to a perforated hollow viscus, antibiotics are administered out of prophylaxis and in high risk patients including immunocompromised patients or patients with American Society of Anesthesiologists (ASA) score > 3, or obesity (QoE B-C moderate-low).

**Statement 5.1.3**

If present, the level of peritoneal contamination and the presence of signs of sepsis and shock are the main factors to guide the administration and duration of an antimicrobial treatment in trauma patients (QoE C low).

**Statement 5.1.4**

There is no evidence confirming the benefit of a long course of antibiotic prophylaxis (> 24 hours) compared to a short course (≤ 24 hours) on abdominal surgical site infection, mortality, or intra-abdominal infection, in the absence of risk factors (obesity, immunosuppression, high ASA score) for post-operative septic complications (QoE B moderate).

**Statement 5.1.5**

The antibiotic of choice should be active against the common bacteria causing surgical site infections in peritonitis, such as *Escherichia coli* or other *Enterobacteriales* or *Clostridiales* (QoE B moderate).

**Statement 5.1.6**

Antibiotic prophylaxis in patients with thoracostomy and penetrating thoracic trauma can prevent infectious complications and protect against empyema and pneumonia (QoE A high).

**Statement 5.1.7**

In blunt chest trauma, antibiotics showed no protective effect against empyema or pneumonia.

(QoE C low).

**Statement 5.1.8**

The use of antibiotic prophylaxis in blunt chest trauma when inserting a chest drain is required, and mostly in chest penetrating trauma to reduce the risk of empyema and pneumonia. (QoE A high).

**Statement 5.1.9**

The use of antibiotics for preventing infection in open limb fractures is recommended (QoE A high).

**Statement 5.1.10**

In soft tissue penetrating injuries, broad spectrum, empirical, intravenous antibiotic therapy should be commenced once deep samples have been obtained, and then tailored once organisms and sensitivities are known. A short course, single agent regimens using cephalosporins in order to prevent adverse outcomes in soft tissue injuries associated with bony injury (open fractures) is recommended. Hand fractures do not require routine prophylaxis. (QoE B moderate).

**Statement 5.1.11**

A specific antibiotic or preferred dosing when employing local antibiotics in the management of fracture related infections cannot be recommended. (QoE C low).

**Statement 5.1.12**

The use of antibiotic prophylaxis as a protective factor for SSI after open reduction and internal fixation of ankle and closed extremity fractures is recommended (QoE A high).

**Statement 5.1.13**

In severely burned patients, the role of an adequate source control including the surgical removal of contaminated material and areas of necrosis and protection of the exposed lesion is crucial in decreasing infectious complications. Antibiotic prophylaxis could protect against septic complications in the high risk patients (QoE C low).

**Recommendation 5.1**

We recommend antibiotic prophylaxis in penetrating (abdominal, thoracic) trauma, in severely burned and in open fractures in elderly patients to decrease septic complications **[Strong recommendation based on a high-moderate quality level of evidence 1A].**

We recommend early empiric antibiotic therapy in patients presenting with signs of sepsis and septic shock and high risk patients (obesity, immunocompromised, high ASA score) in penetrating abdominal trauma, which should be active against common bacteria causing surgical site infections in peritonitis, such as *Escherichia coli* or other *Enterobacteriales* or *Clostridiales* **[Strong recommendation based on a moderate quality level of evidence 1B].**

We recommend against the administration of antibiotics in blunt trauma in absence of signs of sepsis and septic shock **[Strong recommendation based on a moderate-low quality level of evidence 1B].**

**Key Question 5.2:**

***How to control pain in elderly patients admitted for trauma?***

**Statement 5.2.1**

Pain assessment is crucial in obtaining an effective pain control in elderly trauma patients (QoE B-C moderate-low).

**Statement 5.2.2**

Opioids administration should be avoided in elderly patients in the trauma setting to reduce side effects (QoE B-C moderate-low).

**Statement 5.2.3**

Multimodal analgesic approach or “balanced analgesia” including regional and peripheral nerve blocks and neuroaxial analgesia should be implemented in elderly patients pain control, in the trauma setting (QoE B moderate).

**Statement 5.2.4**

Regular intravenous administration of acetaminophen is effective and safe in elderly trauma patients (QoE B-C moderate-low).

**Statement 5.2.5**

Opioids administration for post-traumatic pain in elderly patient should consider a progressive dose reduction because of high risk of morphine accumulation and subsequent over-sedation, respiratory depression and delirium (QoE A high).

**Statement 5.2.6**

Non-pharmacological approaches play an important role in improving trauma pain, including immobilizing limbs and applying dressings or ice packs in conjunction with drug therapy (QoE C low).

**Recommendation 5.2**

We recommend a regular administration of intravenous acetaminophen every 6 hours as first line treatment in managing acute trauma pain in the elderly in a multimodal analgesic approach **[Strong recommendation based on high quality level of evidence 1A].**

We suggest considering to add NSAIDs in elderly patients presenting with severe pain, taking into account potential adverse events and pharmacological interactions **[Weak recommendation based on a moderate quality level of evidence 2B]**.

We recommend the implementation of Multi-Modal-Analgesia approach (MMA) in trauma setting for elderly injured patients including acetaminophen, gabapentinoids, NSAIDs, lidocaine patches, and tramadol and opioids only for breakthrough pain for the shortest period of administration at the lowest effective dose **[Strong recommendation based on a moderate quality level of evidence 1B].**

We recommend peripheral nerve blocks placement in elderly patients with acute hip fractures at the time of presentation to reduce preoperative and postoperative opioid use for analgesia **[Strong recommendation based on a high quality level of evidence 1A].**

We suggest the adoption of epidural analgesia and regional anaesthesia to control severe pain in acute hip fractures in selected elderly patients **[Weak recommendation based on a moderate quality level of evidence 2B].**

In elderly patients with ribs fractures, we recommend the association of systemic analgesic treatment with thoracic epidural and paravertebral blocks to offer an adequate pain control with limited contraindications and improvement in respiratory function, reducing opioid consumption, infections and delirium, if skills are available **[Strong recommendation based on a high quality level of evidence 1A]**.

We recommend to routinely consider the use of epidural or spinal analgesia for management of postoperative pain in elderly patients who undergo major thoracic and abdominal procedures for trauma, if skills are available **[Strong recommendation based on a high-quality level of evidence 1A]**.

We recommend carefully evaluating the use of neuraxial and plexus blocks for patients receiving anticoagulants to avoid bleeding and complications **[Strong recommendation based on a high-quality level of evidence 1A]**.

We suggest the implementation of non-pharmacological measures such as immobilizing limbs and applying dressings or ice packs in conjunction with drug therapy*,* in control acute pain in elderly patients in the trauma setting **[Weak recommendation based on a very low level of evidence 2D].**

**Key Question 5.3:**

***When and how is indicated to administer thrombo-prophylaxis in elderly trauma patients?***

**Statement 5.3.1**

The use of scoring systems to stratify the risk of venous thromboembolism (VTE) of elderly trauma patients is recommended (QoE C low).

**Statement 5.3.2**

Venous thromboembolism (VTE) pharmacological prophylaxis can be avoided in low risk elderly trauma patients (QoE C low).

**Statement 5.3.3**

Venous thromboembolism (VTE) pharmacological prophylaxis is recommended in moderate-high risk elderly trauma patients, if not controindicated (QoE C low).

**Statement 5.3.4**

Mechanical prophylaxis is recommended when pharmacological venous thromboembolism (VTE) prophylaxis is contraindicated (QoE C low).

**Statement 5.3.5**

Venous thromboembolism (VTE) pharmacological prophylaxis should be initiated as soon as possible in moderate-high risk patients and should be delayed of 24 h in case of Central nervous system injuries, active bleeding, coagulopathy, hemodynamic instability or solid organ injury (QoE C low).

**Statement 5.3.6**

Venous thromboembolism (VTE) pharmacological prophylaxis should be held in traumatic brain injury until computed tomography scan shows no progression (QoE C low).

**Statement 5.3.7**

Venous thromboembolism (VTE) pharmacological prophylaxis does not increase the rate of spinal hematoma in spinal injury (QoE C low).

**Statement 5.3.8**

Low Molecular Weight Heparin (LMWH) is recommended over un-fractionated heparin (UFH) to prevent deep vein thrombosis (DVT) (QoE C low).

**Statement 5.3.9**

The recommended dose of LMWH is 30 mg every 12 hours. Dose adjustment according to anti-Xa levels and weight is warranted. In case of renal failure 5000 U of UFH every 8 hours is recommended in elderly trauma patients (QoE C low).

**Statement 5.3.10**

Direct oral anticoagulants (DOACs) or aspirin may be considered as an alternative to heparin in view of better patient’s compliance after clinical stabilisation (QoE C low).

**Recommendation 5.3**

We recommend administering venous thromboembolism prophylaxis with LMWH or UFH as soon as possible in high and moderate risk elderly patients in the trauma setting according to the renal function, weight of the patient and bleeding risk **[Strong recommendation based on a low quality level of evidence 1C].**

If pharmacological prophylaxis of venous thromboembolism is contraindicated, we recommend mechanical prophylaxis **[Strong recommendation based on a low quality level of evidence 1C]**.

**MANAGEMENT OF THE END OF LIFE IN IN TRAUMA SETTING FOR ELDERLY PATIENTS**

**Key Question 6.1:**

**Which are the clinical features and vital signs to define “end of life” in the elderly trauma patient?**

**Statement 6.1.1**

There are no defined clinical features and vital signs to establish the elderly patient at end of life in trauma setting [QoE D very low].

**Statement 6.1.2**

Age alone is not an indication to withhold aggressive therapy [QoE C low].

**Statement 6.1.3**

To define the end of life in elderly patient in trauma setting is a very complex and delicate process. It should consider prognosis in regard to survival outside the acute care setting, the recovery of cognitive ability sufficient to perceive the benefits of treatment, the ability to resume physical activities, the patient’s advance directives, and the involvement of the surrogate decision-maker or healthcare proxy and of the family [QoE D very low].

**Recommendation 6.1**

We recommend discussing in a multidisciplinary approach the end of life in an elderly patient in the trauma setting. The decision should be considering the patient’s directives, family feelings and representatives desires and should be shared **[Strong recommendation based on a low-very low quality of evidence 1D].**

**Key Question 6.2:**

**Could palliative management be useful in the management of an elderly patient at the end of life?**

**Statement 6.2.1**

During the management of an elderly severely injured patient, the early insertion in the decision-making process of palliative medicine consultation improves outcomes, reduces in-hospital mortality and length of stay and improves communication with family, avoiding unnecessary operation. [QoE C low].

**Statement 6.2.2**

Improved palliative care skill training for surgeons should be necessary to be more competent in end-of-life decisions [QoE D very low-quality].

**Recommendation 6.2**

We recommend involving as soon as possible the palliative care team in managing an elderly severely injuried patient at the end-of-life status  **[Strong recommendation based on a low-very low quality level of evidence 1C].**
